# Oral Abstracts

**DOI:** 10.1002/jmrs.664

**Published:** 2023-04-12

## Friday 28 April, 11:00 AM–12:30 PM

## Patient Centred Care in RT

## Person‐centred care: collaborative approach in medical radiations to include lived experiences of health care into curriculum

### Kylie Auld,^1^ John McInerney,^1^ Imalda Devaparanam,^1^ Leah Grinsted,^2^ Daniel Sapkaroski^3^

#### ^1^Monash University, Clayton, Australia ^2^The Royal Melbourne Hospital, Parkville, Australia ^3^Peter MacCallum Cancer Centre, Melbourne, Australia

Contemporary models of healthcare delivery recognise that active patient involvement is now a fundamental principle of safe care. Despite this, healthcare professionals often focus on the technical and medicalised aspects of patient care, failing to provide a holistic model of care.^1^ It has been demonstrated that wide disparity can exist between perceptions of service users and those delivering services on what constitutes high quality person‐centred care.^2^

Applying an interprofessional collaborative approach improves healthcare workers' awareness of each other's professions and skills.^3^ Interprofessional education is vital across health professions to enhance patient outcomes. Promotion of interprofessional education should therefore begin at the tertiary level.

This presentation outlines an interprofessional approach to incorporating patients' voices into the curriculum. Students are given the opportunity to learn from people's lived experiences across diagnostic and therapeutic settings. The perspective of the consumer is presented using vignettes collaboratively designed by professionals across healthcare streams.

Twelve examples of person‐lived stories were obtained from students' clinical practice reflections. Four were then selected based on clinical relevance with input from a clinical educator, a research radiation therapist, academics and a ‘patient’ in the form of a research assistant who collaborated on the project.

With a growing awareness of the importance of person‐centred care, empathy and compassion are core elements for healthcare professionals. This project will further prepare graduates to deliver safe person‐centred care across the healthcare spectrum, providing the opportunity to develop skills, behaviours and attitudes to deliver such care.

**References**

1. Busari JO, Moll FM, Duits AJ. Understanding the impact of interprofessional collaboration on the quality of care: a case report from a small‐scale resource limited health care environment. J Multidiscip Healthc 2017;10:227.
2. Hyde E, Hardy M. Patient centred care in diagnostic radiography (Part 1): perceptions of service users and service deliverers. Radiography 2021;27:8–13.
3. Keller KB, Eggenberger TL, Belkowitz J, Sarsekeyeva M, Zito AR. Implementing successful interprofessional communication opportunities in health care education: a qualitative analysis. Int J Med Educ 2013;4:253.

## Immersive virtual reality coaching and mask‐related anxiety in patients receiving radiotherapy for head and neck cancer

### Daniel Sapkaroski^1^

#### ^1^Peter MacCallum Cancer Centre, Melbourne, Australia

**Background:** Recent findings assessing cancer patients undergoing imaging procedures indicate that 26% present with anxiety, 52% present with sub‐clinical claustrophobia and 27% present with moderate to severe claustrophobia.^1^ Given the burden of reported mask anxiety, management of mask anxiety is key to ensuring treatment acceptance, compliance and tolerability.^2^ The advent of stereoscopic virtual reality may offer an alternative solution in such scenarios. Phase II of this study aimed to better understand mask‐related anxiety before and after the use of an immersive virtual reality patient coaching intervention in head and neck cancer patients who require a thermoplastic mask.

**Methods:** Patients utilised the CETSOL virtual reality clinic patient experience prior to their CT simulation (see Figure). State anxiety scores were measured pre‐ and post‐intervention using a short State Trait Anxiety Inventory questionnaire.^3^ Results were converted to pro‐rated scores with a lower and higher anxiety cut‐off score of 41 and 55, respectively. Descriptive statistics and joint frequency distributions for the discrete variables were derived with 95% confidence intervals calculated using Ward's method. Pearson's chi‐squared test of independence was used to assess the association between each set of variables.

**Results:** Our results will compare the virtual reality intervention group mask anxiety scores to our phase 1 no virtual reality intervention cohort.

**Conclusion:** Early anecdotal evidence indicates favourable outcomes in patient literacy and compliance.


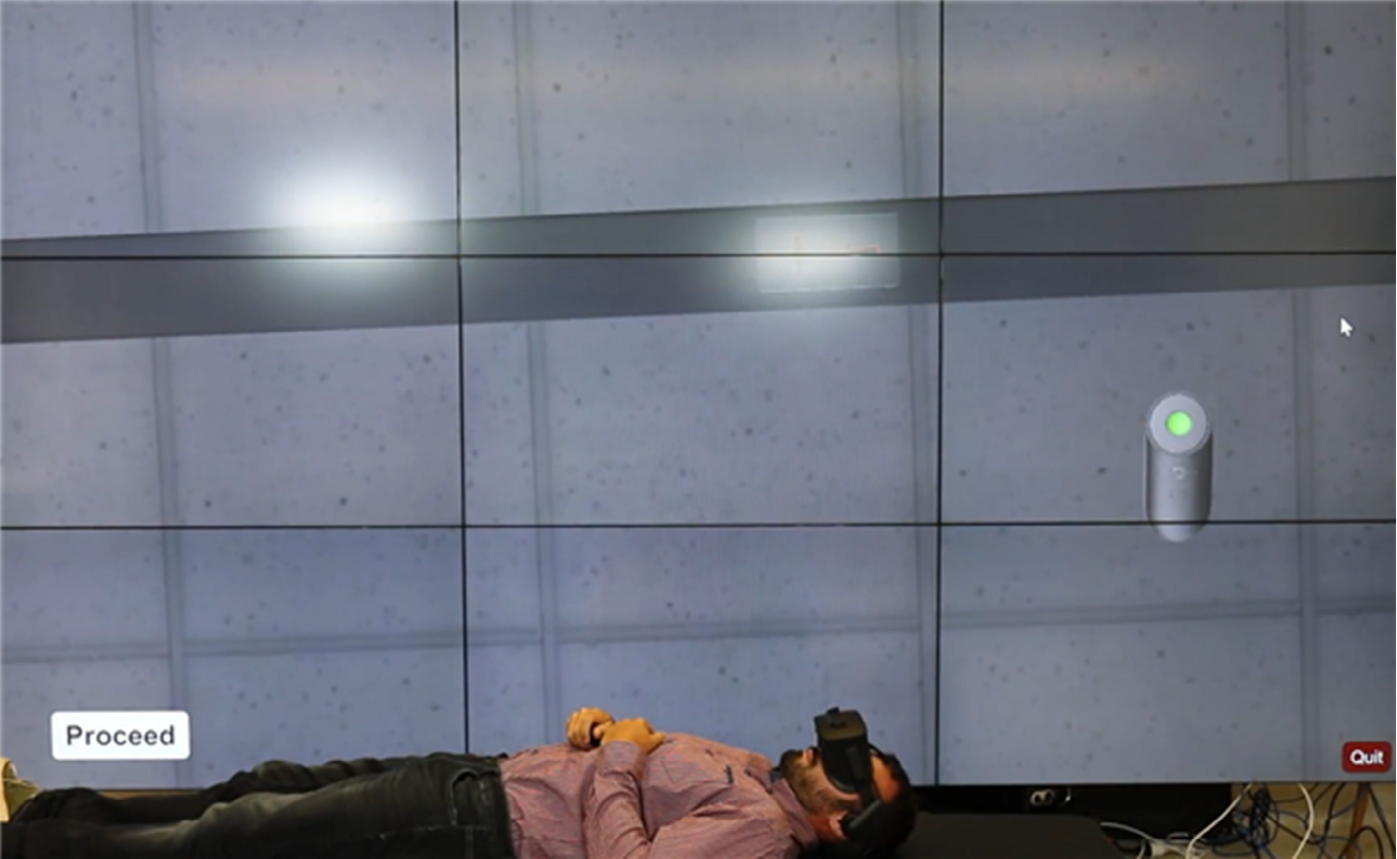


**References**

1. Pelland ME, et al. PO‐0608: Depression, anxiety and claustrophobia in patients undergoing radiotherapy for head and neck cancer. Radiother Oncol 2017;123:S317–S8.
2. Christopher KM, Osazuwa‐Peters N, Dougherty R, et al. Impact of treatment modality on quality of life of head and neck cancer patients: findings from an academic medical institution. Am J Otolaryngology 2017;38:168–73.
3. Marteau TM, Bekker H. The development of a six‐item short‐form of the state scale of the Spielberger State–Trait Anxiety Inventory (STAI). Br J Clin Psychol 1992;31:301–6.

## Pilot evaluation of a patient reported experience survey tool specific to radiation therapy procedures

### Jemma Walsh,^1,2^ Catriona (Cathy) Hargrave,^2,4^ Julie Burbery,^2^ Gregory Rattray,^1^ Lisa Nissen^2,3^

#### ^1^Royal Brisbane and Women's Hospital, Herston, Australia ^2^Queensland University of Technology, Brisbane, Australia ^3^University of Queensland, Brisbane, Australia ^4^Princess Alexandra Hospital, Brisbane, Australia

**Objectives:** Radiation therapy is becoming increasingly more complex with technological advancements often demanding more active patient involvement. Providing clear communication of actions required and getting patient feedback regarding planning and treatment experiences is a priority.^1,2^ This pilot study aimed to establish face validity of a developed patient reported experience survey tool.

**Methods:** Institutional ethics was obtained to recruit left‐sided breast cancer patients treated in breath‐hold to pilot the survey and participate in audio‐recorded cognitive interviews. Interviews were conducted using a hybrid of scripted think aloud and probing formats. Correlation of expected and participant responses were then used to confirm face validity, with additional thematic analysis using the framework method.

**Results:** Response saturation was observed at 15 participants with 10 completing the survey and interviews at all timepoints. Face validity analysis showed minor adjustments were required to the survey, often related to interpretation of symptom terms such as fatigue/tiredness for 75% of participants. Preliminary thematic analysis revealed knowledge of own cancer diagnosis, trust in medical professionals and impact of treatment staff interactions, were themes frequently observed in the lived experience of this patient group.

**Discussion/Conclusion:** Cognitive interviews successfully established face validity of the patient reported experience survey, with only minor adjustments required prior to testing of content validity in a randomised control trial and adaption for different patient cohorts. Preliminary thematic analysis shows interesting insights into cancer patients' interpretation of their treatment experience, providing an example of what can be achieved by engaging with consumers to shape future patient care.

**References**

1. Golden‐Kreutz DM, Thornton LM, Gregorio SWD, et al. Traumatic stress, perceived global stress, and life events: prospectively predicting quality of life in breast cancer patients. Health Psychology 2005;24:288–96.
2. Jimenez‐Jimenez E, Mateos P, Ortiz I, et al. Do patients feel well informed in a radiation oncology service? J Cancer Educ 2018;33:346–51.

## Championing change for paediatric radiation therapy patients: co‐producing a digital quality of life assessment platform

### Mikaela Doig,^1,2^ Andrew Cunningham,^1^ Victoria Bedford,^3^ Hien Le,^4,5^ Matthew O'Connor,^6^ Eva Bezak,^1^ Nayana Parange,^1^ Amanda Hutchinson,^1^ Peter Gorayski,^2,4,5^ Michala Short^1^

#### ^1^University of South Australia, Adelaide, Australia ^2^Icon Cancer Centre, Adelaide, Australia ^3^Cancer Voices South Australia, Adelaide, Australia ^4^Royal Adelaide Hospital, Adelaide, Australia ^5^Australian Bragg Centre for Proton Therapy and Research, Adelaide, Australia ^6^Women's and Children's Hospital, Adelaide, Australia

**Objectives:** Health‐related quality of life (HRQoL) assessment using patient‐reported outcomes has been shown to enhance toxicity detection, reduce hospitalisation rates and improve patient‐clinician communication in paediatric oncology.^1−3^ However, HRQoL assessment is not routinely performed in paediatric radiation therapy clinical practice.^4^ This study aimed to co‐produce a novel digital solution to collect HRQoL outcomes from children receiving radiation therapy and to identify barriers and enablers for clinical implementation in South Australia.

**Methods:** User‐centred co‐design and consolidated framework for implementation research methodologies were followed for this qualitative study.^5–8^ Exploratory semi‐structured interviews were conducted with multi‐disciplinary clinicians from the two hospitals providing care to paediatric radiation therapy patients. Interviews were transcribed verbatim and descriptively coded. Content analysis was performed and themes were categorised using the consolidated framework for implementation research.

**Results:** Thematic saturation was reached after nine interviews with multi‐disciplinary radiation oncology (n = 6) and paediatric oncology (n = 3) professionals. Participants identified digital, clinical and child‐friendly features to inform software development. There was strong support for HRQoL assessment generating new clinical information, guiding patient management, being within scope of practice and complementing existing workflows. Clinical time pressures, transient staffing and parent emotional resources were identified as potential barriers.

**Conclusion:** A digital HRQoL platform was successfully co‐designed for use in routine paediatric radiation therapy care. Barriers and enablers for implementation of HRQoL assessment in paediatric radiation therapy were identified. Study findings will support clinical implementation of HRQoL assessment and directly contribute to enhancing patient care, while providing insight for radiation therapy clinicians seeking to promote change using implementation science.

**References**

1. Leahy AB, Feudtner C, Basch E. Symptom monitoring in paediatric oncology using patient‐reported outcomes: why, how, and where next. Patient‐Centered Outcomes Research 2018;11(2):147–53.
2. Engelen V, van Zwieten M, Koopman H, et al. The influence of patient‐reported outcomes on the discussion of psychosocial issues in children with cancer. Pediatr Blood Cancer 2012;59(1):161–6.
3. Meryk A, Kropshofer G, Hetzer B, et al. Use of daily patient‐reported outcome measurements in paediatric cancer care. JAMA Network Open 2022;5(7):e2223701.
4. Doig M, Bezak E, Parange N, et al. Can we compare the health‐related quality of life of childhood cancer survivors following photon and proton radiation therapy? A systematic review. Cancers 2022;14(16):3937.
5. Slattery P, Saeri AK, Bragge P. Research co‐design in health: a rapid overview of reviews. Health Res Policy Syst 2020;18(1):17.
6. Thabrew H, Fleming T, Hetrick S, Merry S. Co‐design of eHealth interventions with children and young people. Front Psychiatr 2018;9(481).
7. Farao J, Malila B, Conrad N, et al. A user‐centred design framework for mHealth. PLOS ONE 2020;15(8):e0237910.
8. Damschroder L, Aron D, Keith R, et al. Fostering implementation of health services research findings into practice: a consolidated framework for advancing implementation science. Implementation Science 2009;4(1).

## Friday 28 April, 11:00 AM–12:30 PM

## Education in RT: Advances and Opportunities

## Development of a professional certification for radiation therapists undertaking MRI

### Min Ku,^1^ Tanya Morgan^1^

#### ^1^Australian Society for Medical Imaging and Radiation Therapy, Melbourne, Australia

The first MRI linear (MR‐linac) accelerator was installed in Australia in 2019.^1^ With the progression of technology and the ability to facilitate online MR‐guided adaptive radiotherapy, the need for further education for radiation therapists in MRI was critical. Furthermore, radiation therapists undertaking MRI are required to meet the regulatory authority's professional capabilities for medical radiation practice (Domain 1: Key capabilities 8 and 9).^2^

In June 2020, the Queensland University of Technology commenced an online short course in MRI for radiation therapists. A collaboration between Queensland University of Technology and the Australian Society for Medical Imaging and Radiation Therapy (ASMIRT) was created to develop a novel certification to enhance the MRI knowledge and skills of radiation therapists.

ASMIRT committee and reference group members, comprising MRI experts and educationalists, were tasked with the development of a certification syllabus that would reflect the professional skillset of a radiation therapist performing MRI. The collaboration determined a consensus of syllabus content, and the subsequent development of the certification examination questions was undertaken in partnership with the Queensland University of Technology.

A pilot examination was earmarked for November 2022. Organisations that currently undertake MRI simulation and MR‐linac treatments were approached to advise of the pilot, and to seek volunteers to participate. Participants comprised radiation therapist practitioners of varying clinical experience.

The development of this novel certification will provide practitioners and employers with direction for study and educational programs. It presents a benchmark of industry‐standard skill, and formal professional recognition of ability for the MRI radiation therapist.

**References**

1. Tan H. The past and future of adaptive radiation therapy. The Medical Republic. 2021. Available at https://medicalrepublic.com.au/the-past-and-future-of-adaptive-radiation-therapy/58734.
2. Medical Radiation Practice Board of Australia. 2020. Professional capabilities for medical radiation practice. Available at https://www.medicalradiationpracticeboard.gov.au/Registration-Standards/Professional-Capabilities.aspx.

## Exploring advanced practice radiation therapy as an alternative model of care

### Rebecca Height,^1^ Kristie Matthews,^1,2^ Daniel Sapkaroski,^1,2^ Nilgun Touma^1^

#### ^1^Peter MacCallum Cancer Centre, Melbourne, Australia ^2^Monash University, Melbourne, Australia

**Objective:** The personal and professional pressures of the COVID‐19 pandemic, rapid advances in technology, and the introduction of more complex techniques have required our service to consider alternative models of care, enhancing the patient experience and quality of care. The objective of this project was to identify gaps and bottlenecks in care at a large tertiary cancer hospital that may be addressed through the implementation of advance practice radiation therapy roles.

**Method:** During 2022, a comprehensive needs assessment was completed that included: (i) a literature review, (ii) a two‐part mixed methods study of practitioner stakeholders' perception of service gaps and opportunities, (iii) a patient experience survey, (iv) process mapping, and (v) a data mining exercise.

**Results:** The collective data identified key gaps and bottlenecks in care provision in planning (e.g. target delineation, plan approval), treatment (e.g. radiation oncologist‐led image guided radiation therapy) and patient care coordination in complex tumour streams. Resultantly, five advanced practice radiation therapy roles were developed to address these service needs in specialist areas of paediatric/adolescent young adults, palliation (n = 2), breast (n = 2), lung and image guided radiation therapy/adaptive radiation therapy. The focus of each advanced practice radiation therapy role varied according to the needs of the specific tumour stream, but are commonly responsible for enhancing service delivery, clinical leadership and service innovation.

**Discussion:** Advanced practice radiation therapy provides the opportunity to enhance care provision, as well as a pathway towards advanced clinical leadership roles for radiation therapists. When supported by a robust needs assessment, the development of advanced practice radiation therapy can be aligned to service need, which is likely to lead to a more sustainable outcome.

## A simulated clinic to build interprofessional and authentic learning opportunities for shaping future practice

### Eileen Giles,^1^ Katherine Guerrero^1^

#### ^1^University of South Australia, Adelaide, Australia

**Background:** Simulation‐based learning provides an opportunity to increase students' clinical readiness prior to workplace placement.^1^ Cross‐disciplinary learning allows facilitation of interprofessional competency building and improved student appreciation of other health professions.^2^ With this in mind, an authentic learning scenario was planned incorporating two disciplines: nuclear medicine and radiation therapy. This presentation outlines the approach to facilitating an interdisciplinary mock simulated clinic, where experiences were mapped to common professional capabilities.^3^

**Content:** The clinic aim was to replicate physical elements of a nuclear medicine or radiation therapy department. This resemblance to the real‐life scenario is critical to learning.^4^ All students were allocated roles of both the healthcare professional and patient. The patient was encouraged to ask questions to the clinician about the procedure to better understand the other modality. Patient presentations were varied and included limited mobility, communication challenges and varying levels of cooperation. Short appointment times with unexpected events built in, encouraged development of quick recall, teamwork and problem solving.

A student de‐brief using real‐time polling enabled incorporation of student opinions into future mock clinics. They indicated a preference for more information prior to the clinic to better prepare themselves. Although this feeling of unpreparedness was intended to mimic placement, in future clinics expectations of students will be addressed.

**Conclusion:** The mock clinic was a valuable experience for staff and students. A goal to embed authentic assessment through the mock clinic exercise will be explored with future iterations.

**References**

1. Hazell L, Lawrence H, Friedrich‐Nel H. Simulation based learning to facilitate clinical readiness in diagnostic radiography: a meta‐synthesis. Radiography 2020;26:e238–45.
2. Medical Radiation Practice Board of Australia. Professional capabilities for medical radiation practice. 2020. Available at https://www.medicalradiationpracticeboard.gov.au/Registration-Standards/Professional-Capabilities.aspx.
3. Gillan C, Giuliani M, Wong O, et al. Team‐based clinical simulation in radiation medicine: value to attitudes and perceptions of interprofessional collaboration. J Radiother Pract 2015;14(2):117–25.
4. Norman G, Dore K, Grierson L. The minimal relationship between simulation fidelity and transfer of learning. Med Educ 2012;46(7):636–47.

## Radiation therapy students' perceptions of peer group supervision: a pilot study

### Gay Dungey^1^

#### ^1^University of Otago, Wellington, New Zealand

**Objectives:** Research indicates radiation therapy students are at risk of burnout.^1^ Peer group supervision (PGS) is a tool used to help reduce stress, increase reflective practice and help manage professional issues.^2^ This pilot study aimed to investigate the third‐year New Zealand radiation therapy students' perceptions of participating in PGS.

**Methods:** In 2019, all 27 third‐year radiation therapy students were introduced to PGS. At the end of the year, the students were invited to fill in a 14‐item Clinical Supervision Evaluation Questionnaire (CSEQ), answer an open‐ended question and provide demographic data. The CSEQ asks participants to indicate the extent to which they agree with 14 statements related to purpose, process and impact of PGS. The open‐ended question asked if there were anything else they would like to say about participating in PGS as a student. The study utilised both qualitative and quantitative methods.

**Results:** Of the 27 students invited, 22 responded to the questionnaire. The data showed that the students perceived PGS to assist with stress management. They valued having scheduled time out to reflect on practice and appreciated the safety and trust established in the groups.

**Conclusion:** Overall, the radiation therapy students responded positively to PGS. The students felt safe talking about clinical issues in their groups, and they perceived PGS to positively affect their stress management, resulting in new clinical insights and increased self‐awareness. Further research is required to examine the long‐term effects of PGS on patient care and if PGS can help reduce burnout for student radiation therapists.

**References**

1. Probst H, Boylan M, Nelson P, Martin R. Early career resilience: Interdisciplinary insights to support professional education of radiation therapists. J Med Imaging Radiat Sci 2014;45:390–8.
2. Dungey G, Neser H, Sim D. New Zealand radiation therapists' perceptions of peer group supervision as a tool to reduce burnout symptoms in the clinical setting. J Med Radiat Sci 2020;67:225–32.

## Planning experience of new graduate radiation therapists in New Zealand

### Kate Chadwick,^1^ Peter Larsen,^1^ Gay Dungey^1^

#### ^1^University of Otago, Wellington, New Zealand

**Objectives:** This research assessed the preparedness of new graduate radiation therapists (NGRTs) for the clinical practice of planning.

**Methods:** A senior planner from each New Zealand department and NGRTs who completed their degree in 2020 were surveyed after approximately six months of practice. Both were asked about NGRTs preparedness for practice and for feedback on the Bachelor of Radiation Therapy planning curriculum. NGRTs were asked about body sites planned, number of plans completed and planning techniques used. Senior planners were asked about their expectations of NGRTs in planning.

**Results:** NGRTs frequently planned using 3D conformal radiation therapy (3DCRT) or virtual simulation. Commonly planned body sites were those with palliative intent, radical breast and sites more frequently planned using 3DCRT. The departmental sign‐off process sometimes prevented them from generating volumetric modulated arc therapy (VMAT) plans. They suggested more VMAT teaching could be included in the Bachelor of Radiation Therapy. Senior planners expected NGRTs to be able to plan using 3DCRT and VMAT/IMRT. They suggested more clinical workflow teaching in the Bachelor of Radiation Therapy planning curriculum. The majority of NGRTs and senior planners felt the Bachelor of Radiation Therapy prepared NGRTs for clinical practice.

**Conclusion:** The undergraduate degree is preparing NGRTs for clinical practice in planning. 3DCRT and virtual simulation planning techniques remain a core role of NGRTs and a large proportion of clinical workload. NGRTs utilised their VMAT/IMRT planning skills less often during this period of practice, despite being expected to possess these skills. This is a challenge for the undergraduate curriculum and departments as the clinical use of VMAT/IMRT continues to increase.

## Online adaptive radiation therapy in‐house credentialing program for the future of the radiation therapist profession

### Meegan Shepherd,^1,2^ Alexandra Turk,^1^ Leigh Ambrose,^1^ Sarah Bergamin,^1^ Brian Porter,^1^ John Atyeo^1,2^

#### ^1^Northern Sydney Cancer Centre, St Leonards, Australia ^2^Monash University, Clayton, Australia

**Background:** Online adaptive radiotherapy (oART) is enhancing in global application, offering increased accuracy with minimal patient intervention compared to conventional techniques.^1,2^ Implemented at our centre in 2020, Varian Ethos™ oART provides a personalised daily treatment plan, however it requires significant clinical resources. As an early adopter of oART, locally and globally, workflow tasks, responsibilities and radiation therapy training are less well defined.^3^ We report on the development and refinement of a training and credentialing program for future radiation therapist (RT)‐led oART.

**Methods:** Training comprised of online theoretical modules guided by Ethos Super‐Users, observation, emulations and clinical training for RT roles in adaptive planning and treatment. Content was decided by the multi‐disciplinary team with RT proficiency for adaptive roles assessed across a range of cancer subtypes via multimodal methods and inter‐professional craft groups.

**Results:** 13 RTs have been credentialed for at least one of the new adaptive RT roles, with eight RTs fully credentialed and six adaptive support RTs in training. The Adaptive Support (RT2) theoretical module was redesigned to free up time for Ethos Super‐Users.

**Discussion:** Education and training are central components in radiation therapy to ensure quality, equity and safety.^4^ Implementation challenges of oART workflows can be supported by comprehensive training and credentialing with multi‐disciplinary team support. Utilisation of sound educational training methods can demonstrate effective upskilling of RTs in oART. The oART RT credentialing program serves as evidence of competence, and leverages expertise towards advanced practice for the future role of an advanced adapter in RT‐led oART.

**References**

1. Sibolt P, Andersson LM, Calmels L, et al. Clinical implementation of artificial intelligence‐driven cone‐beam computed tomography‐guided online adaptive radiotherapy in the pelvic region. Phys Imaging Radiat Oncol 2020;17:1–7.
2. Byrne M, Archibald‐Heeren B, Hu Y, et al. Varian ethos online adaptive radiotherapy for prostate cancer: early results of contouring accuracy, treatment plan quality, and treatment time. J Appl Clin Med Phys 2022;(1):e13479.
3. Rashid M, Reinlo S, Shelley C, et al. Developing an in‐house adaptive radiotherapy training package for therapeutic radiographers. European Society for Radiotherapy and Oncology. Madrid, Spain. 2021. Royal Surrey County Hospital, Guildford, United Kingdom.
4. Coffey M, Naseer A, Leech M. Exploring radiation therapist education and training. Tech Innov Patient Support Radiat Oncol 2022;24:59–62.

## Friday 28 April, 11:00 AM–12:30 PM

## MRI – MI/NM

## Shaping the future: where are we in the MRI safety landscape?

### Lisa Mittendorff,^2^ Adrienne Young,^2^ Arier Lee,^2^ Jenny Sim^1^

#### ^1^Monash University, Melbourne, Australia ^2^University of Auckland, Auckland, New Zealand

**Introduction:** The MRI radiographer is at the forefront of MRI safety decision‐making and has the primary responsibility to provide high quality, efficient and safe patient care in the MRI environment. With the rapid advancement of MRI technology and with new safety issues emerging, where are we in the MRI safety landscape? This study aimed to provide a snapshot of the preparedness of MRI radiographers in New Zealand and Australia to practise confidently and safely.

**Method:** An online questionnaire, consisting of a series of MRI safety knowledge questions, was distributed in 2018 via social media and relevant professional bodies. The aim of these questions was to establish participants' MRI safety knowledge and clinical decision‐making competency, while simultaneously assessing their confidence levels.

**Results:** Of the 246 completed surveys received, 61% (n = 149) were from Australia, 36% (n = 89) from New Zealand, and 3% (n = 8) from other countries. Findings indicated that current MRI education is preparing MRI radiographers in New Zealand and Australia to practise safely. However, while these radiographers were confident in their MRI safety decision‐making, accuracy levels within some groups need addressing.

**Conclusion:** To develop a consistent level of safe practice for the current workplace and into the future, it is proposed that a minimum level of MRI‐specific education is defined and mandated to practise. Continuing professional development focussing on MRI safety must be encouraged and, if audited as part of registration, could also be mandated. Implementation of a supporting regulatory framework similar to New Zealand is highly recommended for consideration in other countries.

## Longer term effects of distress in MRI and a void in post‐procedure care

### Johnathan Hewis,^1^ Rachel Rossiter,^2^ Marguerite Bramble^2^

#### ^1^Charles Sturt University, Port Macquarie, Australia ^2^Charles Sturt University, Orange, Australia

**Background:** Despite significant improvements in MRI, many individuals experience distress.^1,2^ The MRI experience can result in delayed sequelae that may induce long‐term claustrophobia.^3,4^ Prior research examining this phenomenon has predominantly been underpinned by positivistic scientific methodologies.^4–8^ A deep and holistic qualitative understanding of the lived experience of distress in MRI is currently lacking, particularly in the Australian context.^1,8,9^

**Aim:** To investigate the lived experience of adults who have experienced distress during a clinical MRI examination and give a voice to their perspectives.

**Methods:** Hermeneutic phenomenology is the philosophical framework and qualitative research methodology informing the study design.^10–12^ Participants were recruited from regional New South Wales who had experienced distress during MRI scan within the past six months. Data collection was obtained through semi‐structured interviews conducted via Zoom videoconference. Charles Sturt University HREC approved, No. H19498.

**Results:** Eight participants were interviewed, providing a deep and rich insight into their lived experience of distress. Several themes of meaning emerged during data analysis to include isolation, proprioception and spatiality, distress as an existential threat, self‐soothing strategies, dehumanisation and reduced agency. This oral presentation will specifically explore the interwoven themes of ‘a void in post‐procedure care’ and ‘longer term effects’ after experiencing distress in MRI.

**Conclusions:** Participants regularly described a void in their post‐procedure care where their emotional and psychological needs were not met. Several participants experienced longer term effects including claustrophobic like tendencies and avoidance of confined spaces.

**References**

1. Munn Z, Moola S, Lisy K, et al. Claustrophobia in magnetic resonance imaging: a systematic review and meta‐analysis. Radiography 2015;21(1):59–63.
2. Hudson DM, Heales C, Vine SJ. Radiographer perspectives on current occurrence and management of claustrophobia in MRI. Radiography 2022;28(1):154–61.
3. Klonoff EA, Janata JW, Kaufman B. The use of systematic desensitisation to overcome resistance to magnetic resonance imaging (MRI) scanning. J Behav Ther Exp Psychiatr 1986;17:189–92.
4. Fishbain D, Goldberg M, Labbe E, et al. Long‐term claustrophobia following magnetic resonance imaging. Am J Psychiatr 1988;145:1038–9.
5. Munn Z, Jordan Z. The patient experience of high technology medical imaging: a systematic review of the qualitative evidence. Radiography 2011;17(4):323–31.
6. Mubarak F, Bain K, Anwar SSM. Claustrophobia during magnetic resonance imaging (MRI): cohort of 8 years. Int Neuropsychiatr Dis J 2015;3(4):106–11.
7. van Minde D, Klaming L, Weda H. Pinpointing moments of high anxiety during an MRI examination. Int J Behaviour Med 2014;21:487–95.
8. Hewis J. Do MRI patients tweet? Thematic analysis of patient tweets about their MRI experience. J Med Imaging Radiat Sci 2015;46:396–402.
9. Hofmann B, Svenaeus F. How medical technologies shape the experience of illness. Life Sci Soc Policy 2018;14(3):2–11.
10. Creswell J. Qualitative inquiry and research design: choosing among five approaches. 4th edn. London: Sage Publications Inc; 2014.
11. van Manen M. But is it phenomenology? Qual Health Res 2017;27(6):775–9.
12. van Manen M. Researching lived experience: human science for an action sensitive pedagogy. 2nd edition. New York: Routledge, 2016.

## Simple quantitative planimetric measurement of nigrosome‐1 for the diagnosis of Parkinson's disease in clinical settings

### Minh (Shayne) Chau,^1,2,3^ Marc Agzarian,^3,4^ Robert A Wilcox,^1,3,4^ Andrew Dwyer,^1,3,4^ Eva Bezak,^1^ Gabrielle Todd^5^

#### ^1^University of South Australia, Adelaide, Australia ^2^University of Canberra, Bruce, Australia ^3^Flinders Medical Centre, Bedford Park, Australia ^4^Flinders University, Bedford Park, Australia ^5^South Australian Health and Medical Research Institute, Adelaide, Australia

**Background:** There is currently no definitive diagnostic test for Parkinson's disease. A hypointense appearance of nigrosome‐1 on MRI has been proposed as a biomarker for Parkinson's disease. Current clinical practice involves a subjectively rating the appearance of nigrosome‐1 on susceptibility‐weighted images.^1^ The aim of the current study was to develop and test a simple method for quantifying nigrosome‐1 in clinical settings. We hypothesised that area of nigrosome‐1 hyperintense signal exhibits higher inter‐rater reliability than subjective rating of nigrosome‐1 appearance.

**Methods:** Two experienced neuroradiologists measured area of hyperintense signal in nigrosome‐1 (quantitative method) and rated nigrosome‐1 appearance as present (normal), present but attenuated (abnormal), or absent (abnormal; subjective method) on the right and left side in two groups of patients: 21 with Parkinson's disease (likely abnormal nigrosome‐1; aged 69.6 ± 8.6 years) and 21 with essential tremor (ET; likely normal nigrosome‐1; aged 55.5 ± 20.9 years). Neuroradiologists were blinded to patient group, clinical notes and each other's measurements.

**Results:** Pooled area of hyperintense signal in nigrosome‐1 (across‐sides; 84 data points) was significantly smaller in the Parkinson's disease group (median = 2.1 mm^2^, range = 0–15.8 mm^2^) than in the ET group (median = 8.3 mm^2^, range = 0–15.7 mm^2^; P < 0.001). The two methods had high to very high inter‐rater reliability (subjective: weighted kappa = 0.640, P < 0.001; quantitative: W = 0.733, P = 0.004) and yielded comparable measures of diagnostic accuracy (area under the curve; subjective = 0.881, quantitative = 0.891).

**Conclusion:** Use of this simple quantitative method may improve confidence in longitudinal clinical reporting particularly when nigrosome‐1 is attenuated and for neuroradiologists who are new to nigrosome‐1 MRI reporting.

**Reference**

1. Kim EY, Sung YH, Lee J. Nigrosome 1 imaging: technical considerations and clinical applications. Br J Radiol 2019;92:20180842.

## Systematic review of published literature on MRI skin burns to provide recommendations for MRI practice

### Cassandra Baker,^1,4^ Barbara Nugent,^2,3,4^ Christina Malamateniou^2,5^

#### ^1^Qscan Radiology, Brisbane, Australia ^2^MRI Safety Matters, United Kingdom ^3^Safety, Skills and Improvement Research Collaborative at NHS Education for Scotland, Scotland ^4^City, University of London, London, United Kingdom ^5^HESAV, Switzerland

**Introduction:** MRI is a rapidly evolving modality, generally considered safe due to lack of ionising radiation.^1^ While MRI technology and techniques are improving, many of the safety concerns remain the same as when first established. Patient thermal injuries are the most frequently reported adverse event, accounting for 59% of MRI incidents to the Food and Drug Administration.^2^ Surveys indicate many incidents remain unreported.^3^ Patient thermal injuries are preventable and various methods for their mitigation have been published. However, recommendations can be variable, fragmented and confusing. The aim of this systematic review was to synthesise the evidence base on MRI safety and skin injuries and offer comprehensive recommendations for radiographers to prevent skin thermal injuries.

**Methods:** Six journal databases were searched for sources published January 2010 to September 2022, presenting information on MRI safety and thermal injuries. Of 23,408 articles returned, after careful screening and based on the eligibility criteria, only 36 articles and an additional 19 grey literature sources were included (n = 55).

**Results:** Included studies were examined using thematic analysis to determine if holistic recommendations can be provided to assist radiographers in preventing thermal burns. This resulted in the three simplified recommendations supported by extensive background literature (see Figure). By implementing the above recommendations, it is estimated that 97% of skin burns could be prevented.^3^

**Conclusion:** With thermal injuries continuing to impact MRI safety, strategies to prevent skin burns and heating are essential. Assessing individual risks, rather than blanket policies, will help prevent skin thermal injuries occurring, improving patient care.


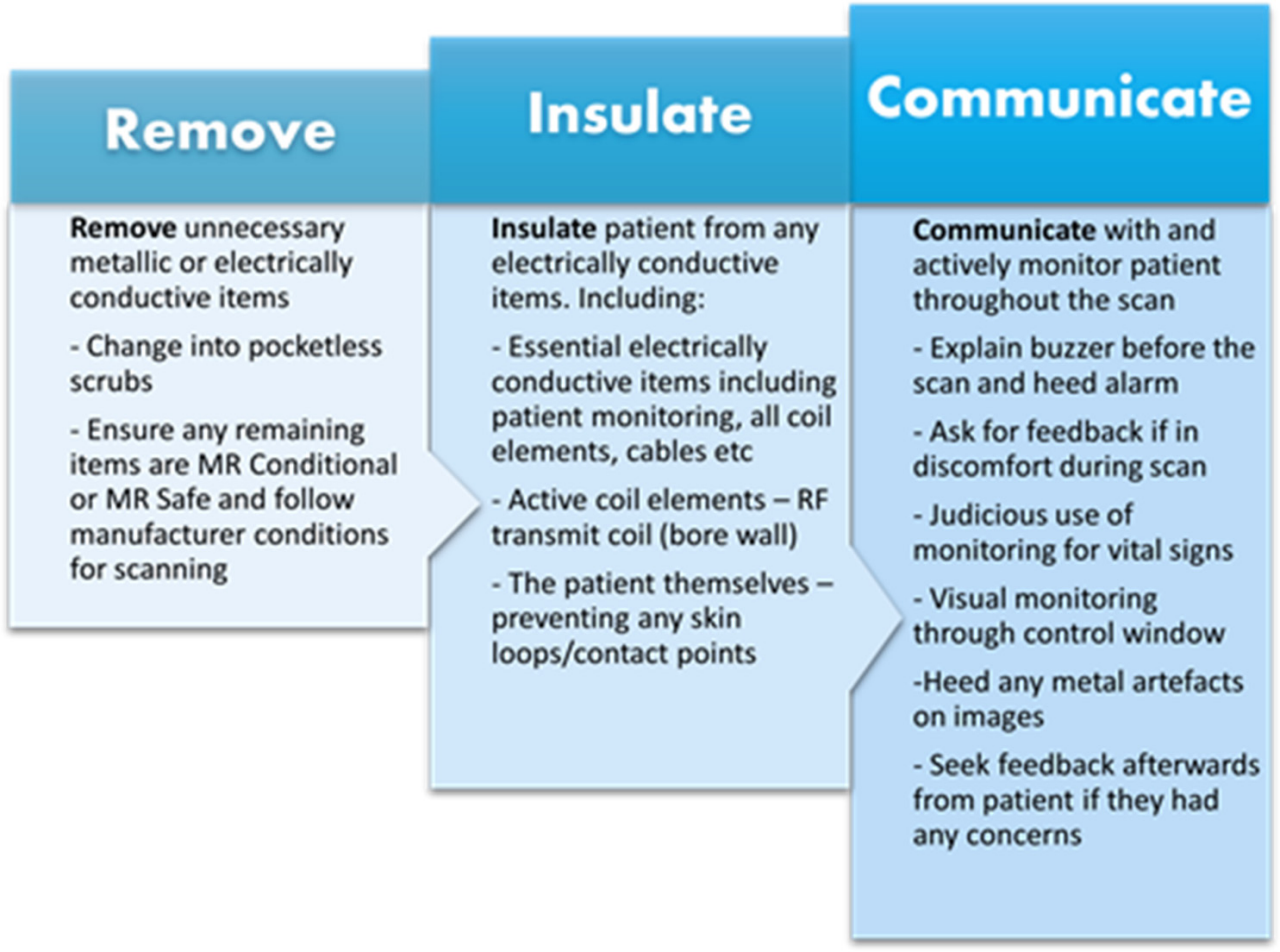


**References**

1. Delfino JG, Krainak DM, Flesher SA, Miller DL. MRI‐related FDA adverse event reports: a 10‐yr review. Med Phys 2019;46(12):5562–71.
2. Kihlberg J, Hansson B, Hall A, Tisell A, Lundberg P. Magnetic resonance imaging incidents are severely underreported: a finding in a multicentre interview survey. Eur Radiol 2021;32(1):477–88.
3. Gilk T. 12 RSNA‐MRI accidents and adverse events. 2012. Available at https://www.youtube.com/watch?v=c-iMRYXhlzg.

## Friday 28 April, 11:00 AM–12:30 PM

## Advancing Planar Radiography – MI

## Can medical radiation practitioners rely on an inherited evidence base?

### Beverly Snaith^1,2^

#### ^1^University of Bradford, Bradford, United Kingdom ^2^Mid Yorkshire Hospitals NHS Trust, Wakefield, United Kingdom

In every diagnostic imaging department and university there are a plethora or textbooks describing the standard general radiography image acquisition techniques and these have largely remained unchanged for over a century. Yet in the same timeframe the evolution of different imaging modalities has provided us greater insight into the human body, not just in a static 2D state but also in a functional sense. Alongside this the profession has developed, and is expected to utilise, evidence‐based practice to optimise person‐centred care. So, based on our increased educational standards and research underpinning it, is it time we critically examined the techniques that we perform every day to ensure they are really evidence‐based and can be applied to every patient presentation? Variations in body habitus and disease processes together with clinician demands require adaptive practices yet our knowledge base may not be keeping up with demands.

This presentation will consider whether historical acquisition techniques can be relied on or whether the profession needs to take bold steps and develop international collaborations to consider (and own) our fundamental practices.

## Check before you send – catching errors before they impact patient care

### Mathew Knox^1^

#### ^1^Sunshine Coast University Hospital, Birtinya, Australia

**Background:** Every year, the Sunshine Coast Hospital & Health Service Medical Imaging Department records hundreds of imaging errors that require fixing after they have gone to the picture archiving and communication system (PACS). These errors can include use of incorrect side markers, images sent under incorrect folders, imaging of incorrect anatomy or, at worst, images of the incorrect patient. The Check Before You Send (CBYS) initiative is implementing a change in attitude and workflow to reduce this number. The author seeks to achieve a higher level of patient safety by increasing imaging accuracy and efficiency, while following established diagnostic imaging accreditation scheme standards.

**Methods:** Control data was collected from a six‐month period of PACS quality control tasks and PACS call‐ins. Staff were educated on the CBYS initiative via PowerPoint presentation and introduced to (a) CBYS posters and modality stickers for all sites in the Sunshine Coast Hospital & Health Service to remind everyone of the importance of checking their work, and (b) a Microsoft Forms questionnaire radiographers/sonographers must submit for all imaging errors to prompt the PACS department to amend the error. The same quality control task and PACS call‐in data will be collected at three and six‐month intervals and compared to the control data. The Microsoft Forms data will be collected to determine how many of the quality control tasks generated are not due to user error.

**Results:** The initiative is ongoing and is due to conclude in March 2023. The author hypothesises that the changes will result in an overall reduction in imaging errors.

## Revisiting the evidence around radiographic positioning: an examination of pelvis centring

### Beverly Snaith,^1,2^ Philip Cosson,^3^ Fiona Mellor,^4^ Andrew England^5^

#### ^1^University of Bradford, Bradford, United Kingdom ^2^Mid Yorkshire Hospitals NHS Trust, Wakefield, United Kingdom ^3^Teeside University, Middlesbrough, United Kingdom ^4^University of Exeter, Exeter, United Kingdom ^5^University College Cork, Cork, Ireland

**Objectives:** Radiography positioning is not underpinned by evidence. With increased awareness of disease, body habitus and postural variation it is incumbent to question our historical foundation. When considering the pelvis radiograph Metzger stated, ‘this region requires most careful study… it is imperative that the … work must be of a superior character’.^1^ This has specific contemporary implications when considering the role of the radiograph in surgical decision making.^2^

Pelvis radiography practice varies across the United Kingdom with resultant disparity in quality.^3,4^ Historical textbooks described various techniques including field centring with anatomical inclusion criteria,^5,6^ although radiographers also use a centring point midline between the anterior superior iliac spine and symphysis pubis.^3^ This presentation will use a prospective cluster randomised research study to consider how projectional radiography can be challenged or embraced.

**Methods:** A multi‐centre prospective trial (STOPPAGE ISRCTN 77100098) compared a control and an interventional site which implemented a new standardised technique based on a femoral landmark. Patient recruitment (≥ 18 years of age, n = 289) enabled review of anatomical inclusion, repeat rate, radiation dose and structural measures, along with radiographer acceptability of the new technique.

**Results:** Analysis is currently underway, and the research design and early findings will be shared.

**Discussion/Conclusion:** Results will shape future practice by showing whether historical methods are suitable for today's patients, or whether further evidence is required to optimise current and future imaging. This is especially pertinent with emerging practice regarding weightbearing imaging.

**References**

1. Metzger JA. Principles and practice of X‐ray technic for diagnosis. Mosby: 1923; p59.
2. Holzer LA, Scholler G, Wagner S, et al. The accuracy of digital templating in uncemented total hip arthroplasty. Hip Arthroplasty 2019;139:263–8.
3. Snaith B, Field L, Lewis EF, Flintham K. Variation in pelvic radiography practice: why can we not standardise image acquisition techniques? Radiography 2019;25:374–7.
4. Parker S, Nagra NS, Kulkarni K, et al. Inadequate pelvic radiographs: implications of not getting it right the first time. Ann R Coll Surg Engl 2017;99:534–9.
5. McNeill C. Roentgen technique. Charles Thomas; 1939.
6. Sante LR. Manual of roentgenological technique. Edward Brothers; 1943.

## Radiographer decision‐making when accepting or rejecting X‐rays: an exploration of non‐technical parameters

### Katrina O'Keefe^1,2^

#### ^1^Queensland Health, Brisbane, Australia ^2^Queensland University of Technology, Brisbane, Australia

**Objectives:** Published reject analysis papers document the technical reasons for rejecting X‐ray images (such as positioning errors), but there is little information surrounding other reasons for rejecting images, or for accepting poor quality images. This presentation reports on a project exploring what factors affect radiographers' decisions to accept or reject. Radiologist opinion on the radiographers' decisions will also be presented.

**Methods:** Ethical clearance was obtained through the relevant participating metropolitan hospital. Radiographers (n = 24) submitted patient cases (n = 115) along with information about their experience, mood and gender, as well as patient age and presentation. Image reject data were also collected. Radiologist opinion on the accepted images was obtained and compared with the decisions of the radiographers.

**Results:** The reported reject rate was 9.24%. When radiographer mood is negative, they are five times more likely to reject an image within a study. No other parameters such as radiographer experience or gender, or patient age or presentation showed significant findings. Radiologist correlation with the opinions of the radiographers was poor.

**Discussion/Conclusion:** Radiographer decision‐making is a complex issue to investigate. This exploratory research showed that radiographer mood significantly affected reject rates, which is outside of the technical factors included in traditional reject analysis publications. The opinion of radiographers showed poor correlation with the opinion of the radiologist, which requires further thought and investigation.

## Re‐creating the compensatory filter: honouring the past by maintaining the design into the future

### Adam Steward^1^

#### ^1^Western Health, Footscray, Australia

The compensatory filter is used in digital imaging to even the density of an imaging field that includes non‐homogeneous tissue types or thicknesses. Anecdotally, the use of the compensatory filter has declined with the advent of digital radiography, where the image can be windowed to allow visualisation in different aspects of the image. This has presented a problem in so far as the compensatory filter is still valuable to the provision of a high‐quality image and that the availability of compensatory filters is now limited.

This presentation details how effective the use of the compensatory filter can still be in digital radiography. It also describes the creation of a compensatory filter for use in a department, the construction of which derives from original compensatory filter design.

## The role of radiographic imaging in disaster victim identification

### Edel Doyle^1,2^

#### ^1^Lumus Imaging, Melbourne, Australia ^2^International Association of Forensic Radiographers, ANZ Branch, Australia

Radiographic imaging plays an important role in disaster victim identification. The modalities used will be determined by the location and availability of resources, taking the needs of living casualties into consideration too.

This presentation will provide an overview of the contribution of dental X‐rays, fluoroscopy, radiography and CT to the disaster victim identification process, while applying forensic principles to avoid errors and maintain continuity of evidence.

## From digital to material: how radiology is shaping the future of medical 3D printing

### Alex Hollingsworth,^1^ Xanthe Keneally^1^

#### ^1^Princess Alexandra Hospital, Brisbane, Australia

You fall from a height, stumbled down the stairs or maybe you had a bit too much to drink the night before, you do not remember. All you know right now is the doctor is telling you that your ankle is injured, and traditional surgeries will not offer many options. You have heard about 3D printing. Can this technology offer a solution?

Medical imaging plays an essential role in producing the models used for 3D printing within the medical field. The vision of many health researchers and professionals is to utilise this rapidly evolving technology to help improve the lives of their patients, provide clinicians with visualisation tools and educate their students and colleagues. Using CT and MRI datasets, digital models can be designed and printed to replicate a missing or damaged bone, produce a patient matched cutting guide for a difficult tumour removal or manufacture a surgical tool matched to the patient's individual anatomy.

We will examine the history and how 3D printing technologies differ between the consumer and medical worlds, the applications for 3D printing within the medical space, where radiographers currently fit into the process and how we can contribute to the implementation of this technology. We will also investigate its limitations and briefly touch on the future of 3D printing in the medical field to see how it has already been implemented.

## Friday 28 April, 11:00 AM–12:30 PM

## Mixed – NM

## Addressing health inequity and improving access to prostate‐specific membrane antigen imaging in rural New Zealand through PSMA SPECT

### Prudence Lamerton,^1^ Rachelle Steyn^1^

#### ^1^Te Whatu Ora, Hawkes Bay Hospital, Stortford Lodge, Hastings, New Zealand

**Introduction**: To date, no publicly funded PET/CT imaging for New Zealand patients exist. This has led to significant inequalities in PET/CT access, particularly for patients living in rural regions and our Indigenous people.

**Background**: Hawkes Bay serves a population of 182,700 people (75% New Zealand European, 32.6% Maori/Pacific).^1^ In New Zealand, 3900 men are diagnosed with prostate cancer per year of which 420 are of Maori/Pacific ethnicity.^2^ Prostate‐specific membrane antigen (PSMA) PET/CT became available in New Zealand in 2017. During the six years after introduction only 97 PSMA PET/CT scans have been performed. Of even greater concern is that 86% of these were performed on New Zealand European men and only 12% were performed on Maori/Pacific men.

In May 2021, PSMA single photon emission computed tomography (SPECT) CT was introduced as an alternative to patients who could not access PSMA PET/CT. During the 1.5 years after introduction, 70 PSMA SPECT CT scans have been performed with an ethnicity breakdown of New Zealand European (82%) and Maori/Pacific (15%).

**Conclusion**: The introduction of PSMA SPECT CT in May 2021 has led to a threefold increase in access to PSMA imaging in our region, in turn providing a more equitable health service for the public health sector in Hawkes Bay.

**References**

1. Stat NZ. Hawkes Bay region. 2018. Available at https://www.stats.govt.nz/tools/2018‐census‐place‐summaries/hawkes‐bay‐region.
2. Cancer Control Agency. 10 most common cancers. Available at https://teaho.govt.nz/cancer‐numbers/common‐cancers.

## Minimising patient radiation exposure through individually tailoring patients' administered activity: a systematic review

### Mitchell Burton,^1^ Christopher Skilton,^1,2^ David Lyall^1^

#### ^1^The University of Newcastle, Callaghan, Australia ^2^Hunter New England Imaging (Nuclear Medicine & PET), Tamworth, Australia

**Objectives:** Patients risk from radiation exposure is directly related to the administered activity of a radiopharmaceutical and individual anthropomorphic parameters. Individually tailoring administered activity is one method to minimise this risk and maintain diagnostic image quality. The aim of this study was to identify ways to individually tailor administered activity to patients as a way minimise the radiation exposure while maintaining image quality.

**Methods:** Five literature databases were searched for reports published after 2000, in English. Key search terms included “nuclear medicine”, “SPECT”, “PET”, “radiopharmaceutical”, “radiation exposure”, and “image quality”. Studies performed in humans from peer‐reviewed journals were screened and selected. Reports then underwent quality assessment using the Joanna Briggs Institute checklist tools. Relevant data were extracted from included reports. Narrative synthesis of the results was conducted due to the heterogenous nature of the articles.

**Results:** 31 peer‐reviewed papers were included. Most studies were deemed to have some level of publication bias. For general nuclear medicine procedures only myocardial perfusion reports were identified whereas a range of positron emission tomography studies were identified. To individually tailor the administered activity most reports employed a weight‐based scale. Other methods included a body mass index‐based scale, a reference patient weight‐based formula and a weight‐based quadratic formula.

**Conclusion:** Anthropomorphic parameters are suitable to tailor the administered activity to reduce radiation exposure while achieving high quality general nuclear medicine procedures. Future research into the combination of anthropomorphic parameters to tailor the administered activity is required.

## Dose optimisation in bone scintigraphy

### Steven Kentish,^1^ Christopher Skilton,^1,2^ David Lyall^1^

#### ^1^The University of Newcastle, Callaghan, Australia ^2^Hunter New England Imaging (Nuclear Medicine & PET), Tamworth, Australia

**Objectives:** The aim of this study was to determine a methodology by which patient radiation doses can be minimised while maintaining image quality in bone scintigraphy studies.

**Methods:** In this retrospective audit‐based study, images and data for 55 patients were retrieved and resampled to simulate a series of reduced dose whole‐body bone scans, coming to a total of 171 images. Images were scored against a rubric, with a subjective 10‐point scale on whether the image was deemed of satisfactory diagnostic quality.

**Results:** The mean dose in current practice was found to be 817 MBq, providing a dose per weight for the average patient of 10.74 MBq.kg‐1. All 171 images were reviewed, with scores, ratings and satisfaction acquired for all. Image score and image rating were found to have linear but imprecise relationships with both dose and dose per weight. Image satisfaction was demonstrated to have a close relationship with both dose and dose per weight. Using a satisfaction driven weight‐based model with current data, patients less than 70 kg could receive lower doses while maintaining current image quality.

**Conclusion:** This study demonstrates a reproducible, applicable methodology by which a given centre can use a linear, weight‐based dosing regimen up until the threshold at which this produces a dose equal to current practice. This study recommends performing regular internal audits in order to more vigorously apply and maintain doses as low as reasonably achievable through regular readjustment of dosimetry practice.

## Towards a future ready nuclear medicine service: the effect of latest guidelines on nuclear medicine set‐up

### Jin Tang Tay,^1^ Tee Meng Tan,^1^ Yi Wei Tan,^1^ Yi Hong Chng^1^

#### ^1^Woodlands Health, Singapore

To safeguard patient safety, the Ministry of Health Singapore released a set of standards for the provision of nuclear medicine services in 2019.^1^ Alongside the Radiation Protection Act 2007,^2^ the new guideline called for a change in the nuclear medicine set‐up for newer hospitals. This study investigates the effect the guidelines had on the set‐up of nuclear facilities in Woodlands Health, serving as a reference for future hospital planning.

The new standard was compared to existing planning provisions and further evaluated through site visits to established nuclear medicine facilities and engagement with professional team members. Two significant discrepancies were observed.

First, a ‘hot‐toilet’, used exclusively by ‘hot’ patients, is now required to be linked to a decay sewerage management system for the storage of radioactive biological waste until safe for discharge into the public sewage system. To achieve this for Woodlands Health, a contractor specialising in the building of such facilities was consulted.

Second, an exercise was initiated to re‐design the nuclear medicine facility layout to fulfil the need for a segregated lead‐lined waiting area. Theoretical calculation on the expected radiation exposure based on workload was performed to account for the amount of lead‐lining required to protect staff working in the area.

With the enforcement of guidelines by authorities in recent years, both older and new hospitals will eventually have to make changes to safeguard public and staff safety. This study records the adjustments made during the set‐up of the Woodlands Health nuclear facilities and will be a good reference for other hospitals subjected to similar requirements.

**References**

1. Ministry of Health Singapore. Standards for the provision of nuclear medicine, imaging, therapy and assay services. Available at https://www.moh.gov.sg/docs/librariesprovider5/licensing-terms-and-conditions/standards-for-the-provision-of-nuclear-medicine-services_28052019_final.pdf.
2. Radiation Protection Act 2007. Available at https://sso.agc.gov.sg/Act/RPA2007.

## Friday 28 April, 1:30 PM–3:00 PM

## Improving Patient Experience – RT

## Patient‐centred care, emotional intelligence and clinical placement: a scoping review

### Debra Lee,^1^ Tracy Burrows,^1^ Daphne James,^1^ Ross Wilkinson,^1^ Yolanda Surjan^1^

#### The University of Newcastle, Callaghan, Australia

**Overview:** Historically, higher education in health care has focussed on delivering technical and clinical content as the driver for student success. More recently, successful healthcare learning has been linked to the student's ability to demonstrate empathetic behaviour towards patients, maintain high‐level communication skills and regulate their emotional reactions.^1^ These skills form the foundations of emotional intelligence and have been linked to increased patient satisfaction levels.^2^

**Aim:** A scoping review was conducted to evaluate emotional intelligence measurement tools and interventions used in undergraduate medicine, nursing and allied health cohorts.

**Methods:** Studies were identified from PsychInfo, Medline/PubMed, CINAHL and Embase databases, published from 1990 to 2021; 221 papers were selected for inclusion in this review. Data were evaluated to determine the outcomes of emotional intelligence evaluation and intervention for undergraduate students.

**Results:** Initial review of the literature identified 32 different emotional intelligence assessments in medical, nursing and health education. Many studies additionally reported on student academic and clinical performance, stress, empathy and mental health status as related factors to emotional intelligence.

**Conclusion:** Maintaining compassion and empathy while providing high standards of clinical care are hallmarks of an emotionally intelligent health practitioner. From an academic perspective, research indicates that educators should foster not only high‐level technical learning in students, but also promote non‐cognitive clinical practice characteristics to ensure a patient‐centred focus of care. The assessment of technical and clinical competence in medical radiation science students is essential. Still, the evaluation of their ability to provide patient‐focussed care in an empathetic and prudent manner is often understated.

**References**

1. Mann KV, Ruedy J, Millar N, Andreou P. Achievement of non‐cognitive goals of undergraduate medical education: perceptions of medical students, residents, faculty and other health professionals. Med Educ 2005;39(1):40–8.
2. Jangland E, Gunningberg L, Carlsso M. Patients' and relatives' complaints about encounters and communication in health care: evidence for quality improvement. Patient Educ Couns 2009;75(2):199–204.

## A national survey of New Zealand cancer patients' preferences for radiation treatment information

### Alannah Flockton,^1,2^ Aidan Leong^1,2^

#### ^1^University of Otago, Wellington, New Zealand ^2^Bowen Icon Cancer Centre, Wellington, New Zealand

**Objectives:** Evidence shows cancer patients receiving radiation treatment have a diverse range of information needs.^1–3^ However, there is a lack of data specific to the information needs of New Zealand patients. This cross‐sectional survey captured cancer patients' preferences for radiation treatment information. Specific preferences were assessed regarding the scope of information needs and the satisfaction with which those needs were currently being met. Results will provide the largest multi‐centre dataset of patient information preferences in New Zealand and aims to shape future initiatives for radiation treatment education.

**Methods:** A one‐page, paper‐based survey was offered to all eligible patients undergoing radiation treatment at six of 10 departments across New Zealand. The survey captured patient demographics and information preferences/satisfaction using Likert scales and one free‐text question.

**Results:** All departments administered the survey during the same week in November 2022. Data analysis occurred during December 2022 and results will be presented. Descriptive statistics will be calculated for all questions and correlations with demographics evaluated. Free‐text responses will be analysed using directed content analysis to identify common themes.

**Discussion/Conclusion:** This survey will provide researchers and clinical departments with valuable data which current New Zealand radiation patient education practices can be evaluated against, as well as shape future initiatives. Furthermore, trends in patient information preferences maybe identified based on demographic and treatment‐related characteristics. A nationwide evaluation of patient preferences is timely in the context of COVID‐19 with increased utilisation of remote platforms for engagement and with the establishment of a new single New Zealand health service.

**References**

1. Zeguers M, de Haes HC, Zandbelt LC, et al. The information needs of new radiotherapy patients: how to measure? Do they want to know everything? And if not, why? Int J Radiat Oncol Biol Phys 2012;82(1):418–24.
2. Halkett GK, Kristjanson LJ, Lobb E, et al. Information needs and preferences of women as they proceed through radiotherapy for breast cancer. Patient Educa Counsel 2012;86(3):396–404.
3. Douma KF, Koning CC, Zandbelt LC, de Haes HC, Smets E. Do patients' information needs decrease over the course of radiotherapy? Support Care Cancer 2012;20(9):2167–76.

## Randomised trial of person‐centred versus standard radiation therapy model of care for breast cancer patients (PERSON study)

### Michael Velec,^1,2^ Vivian Hoang,^1^ Angela Cashell,^1,2^ Kirsten Bryant,^1^ Susan Chen,^1^ Ryan Hyvarinen,^1^ Suyeon Kim,^1^ Grace Lee,^1,2^ Susanne Lofgren,^1^ Sajida Moledina,^1^ Diana Powell,^1^ Anita Vloet,^1^ Olive Wong,^1^ Amy Liu,^1^ Anna Santiago,^1^ Joseph Nachman,^1^ Jennifer Croke,^1,2^ Anthony Fyles,^1,2^ Rachel Glicksman,^1,2^ Ezra Hahn,^1,2^ Kathy Han,^1,2^ Joelle Helou,^1,2^ Fei‐Fei Liu,^1,2^ Danille Rodin,^1,2^ Anne Koch^1,2^

#### ^1^Princess Margaret Cancer Centre, Toronto, Canada ^2^University of Toronto, Toronto, Canada

**Objective:** To determine if a novel person‐centred model of care, consisting of radiation therapist‐led education and continuity of care, was more effective at reducing anxiety compared to standard care for patients with breast cancer.

**Methods:** Patients requiring locoregional breast radiotherapy were randomised (1:1) to the intervention or standard care. The intervention was a one‐on‐one, in‐person pre‐CT education session with a radiation therapist who subsequently performed CT, planning, treatment delivery and support for the same patient. Standard care had no dedicated education and was usually delivered by many different radiation therapists. The primary outcome was change in anxiety from baseline during radiotherapy. Secondary outcomes were changes in self‐efficacy, treatment concerns and health engagement levels as well as post‐treatment satisfaction. Optional semi‐structured interviews were completed to thematically analyse patients' lived experiences with radiotherapy.

**Results:** Patients (109 total) were randomised to the intervention (54) or standard care (55). Most received 40 Gy/15 fractions (94%) using a 4‐field technique (93%) in active breath hold (57%). The magnitude of anxiety improvement during treatment was similar between arms (P = 0.682). Procedural concerns improved in the intervention arm (P = 0.001). Other secondary outcomes were similar between arms. Satisfaction significantly (P < 0.05) improved with the intervention for provision of information, members of the care team and overall care. Interview analysis (20 intervention, 21 standard care) revealed the radiation therapist‐led education was broadly valued and having one consistent radiation therapist was valuable for especially vulnerable individuals.

**Conclusion:** A person‐centred radiation therapist‐led model of care improved breast cancer patients' preparation for radiotherapy and their subsequent experiences and satisfaction with care.

## Champions of change: reviewing patient pathways towards improved outcomes for pelvic cancer radiation therapy

### Jennifer Trimboli,^1^ Lisa Mctye^1^

#### ^1^Central Adelaide Local Health Network, Adelaide, Australia

**Background:** The ability of patients undertaking pelvic irradiation to successfully predict bladder fullness is difficult even when utilising a well‐informed protocolised bladder filling instruction. The use of an ultrasonic scanning device can significantly improve the accuracy of this prediction. The referral process for ultrasonic scanning a patient's bladder pre‐treatment in the radiation oncology department has increased patient pathways and the impact of this increase to treatment timelines in this cohort of patients is the subject of this review.

**Discussion:** This review focussed on reducing patient timelines within the department while improving multi‐disciplinary communication and procedures. Developing a baseline pelvic patient pathway through the department helped define limitations of the current practice and gave insight into interventions to improve patient outcomes. Recommendations were made to implement a new system allowing radiation therapy or nursing staff to triage the patients relative to bladder fullness before they present for treatment. It was also recommended that the hospital's electronic medical record system be utilised to record scanning results, to simplify communication, as the electronic medical record is universally utilised across disciplines. It was also recommended that an additional ultrasonic scanning device be acquired, and a training program developed to train radiation therapist staff in the use of the ultrasonic scanning device in order to share the clinical load of this intervention.

**Conclusion:** This radiation therapist‐led initiative supports the improvement of patient experiences by streamlining an historically unpredictable and potentially stressful step in pelvic irradiation treatment. It also champions change through multi‐disciplinary collaboration enabling improved patient outcomes.

## Development of a paediatric radiation therapy program to allay anxiety

### Xiao Wei Tan^1^

#### ^1^National Cancer Centre, Singapore

The management of children undergoing radiation therapy is challenging. The use of immobilisation devices and the need to remain still may cause anxiety in children, which may result in the need for sedation.^1,2^ We sought to optimise our service to reduce the overall psychological burden on our patients and hopefully reduce sedation requirements.

We partnered with child life therapists and art therapists from the children's hospital. The improvements set in place include pre‐treatment visits by the child life therapists in the inpatient and/or home setting, which includes playing with toy‐sized models of our treatment equipment. A pre‐treatment facility tour program has been introduced to allow an opportunity for caregivers and the child to familiarise themselves with the radiation therapists and treatment environment. A play therapy element is included, where the child is introduced to the immobilisation gadgets. To provide continuity, our educational booklets include a patient journey story that spans the children's hospital and our treatment unit in an adult hospital. We are developing a virtual reality facility tour environment that will be run on a headset, which includes characters from the children's educational booklets. A mask‐painting program is also currently under development.

Radiation therapy is an important aspect of paediatric cancer treatment, but it can cause anxiety in children, which may result in a need for sedation. We have optimised our paediatric radiotherapy program to address this and facilitate the delivery of holistic care.

**References**

1. Tyc VL, Klosky JL, Kronenberg M, de Armendi AJ, Merchant TE. Children's distress in anticipation of radiation therapy procedures. Child Health Care 2002;31(1):11–27.
2. Klosky JL, Tyc VL, Tong X, et al. Predicting paediatric distress during radiation therapy procedures: the role of medical, psychosocial, and demographic factors. Paediatrics 2007;119(5):e1159–66.

## Friday 28 April, 1:30 PM–3:00 PM

## Advancing RT Practice – RT

## Radiation therapist‐led lung stereotactic ablative body radiotherapy treatment in the absence of radiation oncologist

### Menglei Chao,^1^ Maryam Hazem,^1^ Kylie Unicomb,^1^ Shamira Cross,^2^ Roland Yeghiaian‐Alvandi^1^

#### ^1^Nepean Cancer and Wellbeing Centre, Melbourne, Australia ^2^Nepean Blue Mountains Local Health District, Penrith, Australia

**Objectives:** The aim of this study was to assess the level of variability of cone beam computed tomography (CBCT) image matches of radiation therapists (RTs) compared to radiation oncologists (ROs) for patients undergoing lung stereotactic ablative body radiotherapy (SABR) treatment.

**Methods:** Day 1 pre‐treatment CBCT of 10 lung SABR patients were retrospectively selected for offline imaging matches. The patient cohort included six free‐breathing patients and four deep inspiration breath hold patients. Two ROs, 27 RTs and five RT pairs performed offline imaging matches three times on three different days on the 10 patient datasets. Translational and rotational corrections as well as image matching time were collected from each CBCT match. Delivered day 1 match results were used as the gold standard. In total, 1019 CBCT and 6114 data points were collected for data analysis. Inter‐observer and intra‐observer variations were analysed as functions of profession, number of staff, years of SABR experience and breath hold methods.

**Results:** Excellent correlation in CBCT image matching were observed between RTs and ROs. Time spent on imaging matching was significantly lower in paired RTs compared to individual RTs. There were more variations in sup/inf direction and all rotational directions observed in free‐breathing patients compared to deep inspiration breath hold patients. Staff with different lung SABR experiences all showed excellent correlation to day 1, however slightly more matches over threshold were observed in the less than three‐year SABR experience group.

**Discussion/Conclusion:** RTs who underwent current departmental lung SABR imaging training/credentialing process can provide consistent CBCT matching results in the absence of ROs for day 1 lung SABR treatment.

## Embracing the present: credentialing considerations for radiation therapist‐led SABR IGRT

### Kenton Thompson,^1^ Glenn Trainor^1^

#### ^1^Peter MacCallum Cancer Centre, Melbourne, Australia

The ‘core scope’ of clinical practice for radiation therapists (RT) refers to those aspects of clinical practice that can reasonably be expected to be undertaken by all RTs. Over time, organisational needs and capabilities change, technologies progress and new services may be proposed. These factors underpin the need to routinely review and renew RTs' scope of clinical practice. Specific credentialing and determination of a specific scope of clinical practice is required where it cannot be reasonably assumed the RT's qualifications include the specific competency.^1^

Stereotactic ablative body radiotherapy (SABR) adoption continues to increase and is no longer reserved solely for large centres. A high degree of accuracy is required for SABR due to the high dose per fraction and proximity of critical structures. In many departments a radiation oncologist is required to be present for each fraction to assist with and confirm the match for SABR treatments. RT‐led SABR image‐guided radiation therapy (IGRT) can help reduce the time burden on the radiation oncologist and increase patient workflow efficiency.

Organisations are at different stages of implementing RT‐led SABR IGRT. In order to credential for the specific scope of RT‐led SABR IGRT, additional training, experience and ongoing proficiency all need to be considered taking into account service provision and organisation capabilities. This presentation will detail the steps undertaken by our organisation to initiate RT‐led SABR IGRT and changes to credentialing over time following review. Key recommendations and lessons learnt will be shared, such that others can learn from our experience.

**Reference**

1. Australian Commission on Safety and Quality in Health Care. Credentialing health practitioners and defining their scope of clinical practice: a guide for managers and practitioners. Sydney: ACSQHC, 2015.

## Perceptions and understanding of advanced practice radiation therapy in Victoria: defining our professional future

### Rebecca Height,^1^ Kristie Matthews,^1,2^ Daniel Sapkaroski,^1,2^ Nilgun Touma^1^

#### ^1^Peter MacCallum Cancer Centre, Melbourne, Australia ^2^Monash University, Melbourne, Australia

**Objective:** To explore the understanding and perceptions of radiation oncology health professionals (oncologists, therapists, physicists, engineers and nurses) with respect to advanced practice radiation therapy roles, in order to define the challenges and benefits of introducing this model of care at a large tertiary cancer hospital.

**Methods:** A two‐part mixed methods study design was employed in early 2022. The quantitative arm used a published validated survey to collect data from all radiation medicine disciplines across the service. This was followed by qualitative focus group data collection from the same cohort. Analysis utilised descriptive statistics and thematic analysis.

**Results:** 100 complete responses were received for the online survey. Eight focus groups and two interviews were conducted with 51 participants. Data indicated broad multi‐disciplinary support for advanced practice radiation therapy (89%), and 87% believed that patients would benefit from the establishment of advanced practice radiation therapy. Concerns were identified regarding professional boundaries, understanding of the roles, and educational requirements to fulfil the role.

**Discussion:** Advanced practice radiation therapy roles are well established internationally and have been shown to improve the quality of, and access to, care. However, implementation locally is inconsistent. Local multi‐disciplinary support for this alternative model of care is strong, yet there continues to be profession‐specific concerns which may inhibit broader implementation. Engaging the various professions to explore these concerns while showing sustainable improvements to care will strengthen the outcomes of these roles.

## Implementation of a radiation therapist‐led referral program to Canteen

### Maiko Crispin,^1^ Claire King,^1^ Pandora Patterson,^2^ Fiona McDonald,^2^ Xiomara Skrabal,^2^ Thilo Schuler^1,3^

#### ^1^Northern Sydney Cancer Centre, Royal North Shore Hospital, St Leonards, Australia ^2^Canteen Australia, Newtown, Australia ^3^Australian Institute of Health Innovation, Macquarie University, North Ryde, Australia

**Objectives:** A parental cancer diagnosis has psychosocial impacts on the entire family. In 2020, a radiation therapist‐led program was introduced to identify and refer patients who are parents of young persons to Canteen support services. Electronic patient‐reported outcome (ePRO) surveys screened for referral candidates and radiation therapists approached them to provide Canteen information and offer referral. We report the monthly referral rates pre‐ and post‐program introduction and results from a patient experience survey.

**Methods:** An audit comparing monthly referral numbers in the year prior to program implementation and since its commencement was performed. We surveyed all ePRO‐identified referral candidates (parents of 0 to 25‐year‐olds receiving radiotherapy). We report responses to the following 5‐point Likert scale questions regarding the program: (i) acceptability, (ii) usefulness and (iii) satisfaction.

**Results:** Between July 2020 and October 2022, 50 patients and 93 young people were referred. Compared to 2019, the monthly referral rate in this post‐implementation period was substantially increased from 0.7 to 1.9 referrals per month. The experience survey completion rate was 31% (48/153) and the responses showed 92% and 8% found the pathway acceptable or somewhat acceptable, respectively. There were no neutral or non‐acceptable responses; 93% found it very useful or useful, and 90% were very satisfied or satisfied with the verbal information they received from radiation therapists.

**Discussion/Conclusion:** A radiation therapist‐led referral pathway has increased patient access to support services and is highly valued by patients.

## Shaping the future of stereotactic body radiation therapy: radiation therapist‐led image guided radiation therapy credentialing program for SBRT lung

### Kelsie Henry,^1^ Beth Effeney,^1^ Cathy Hargrave,^1^ Jillian Becker,^1^ Andrew Pullar^1^

#### ^1^Princess Alexandra Hospital, Brisbane, Australia

As high geometric precision is required for stereotactic body radiation therapy (SBRT) delivery, pre‐treatment image guided radiation therapy (IGRT) is necessary to ensure accurate targeting of the treatment fields. Since the introduction of SBRT into the clinical setting, the radiation oncologist has standardly been required to attend every treatment fraction and approve the online IGRT match before treatment can be delivered. Following the guidelines established by the Faculty of Radiation Oncology,^1^ a departmental IGRT training and credentialing program was developed for radiation therapists. The aim of the program was to enable radiation therapists to autonomously perform the online IGRT match for SBRT lung cases, removing the requirement for radiation oncologist attendance at every treatment. A multi‐disciplinary team was formed to design and implement the program, with the key goal of providing radiation therapists with the knowledge to make safe and appropriate image matching decisions for a range of SBRT lung scenarios. The robust training program includes written resources, extensive offline training and testing cases that simulate the online environment. All training and testing cases are compared to a radiation oncologist gold standard match and represent broad decision‐making principles. A research project is currently being conducted to assess the effectiveness of the credentialing program, including its adaptation to radiation oncology departments with differing vendor equipment and technologies. This presentation will discuss the design of the program, challenges, modifications, initial findings of the research project and future directions.

**Reference**

1. Foote M, Bailey M, Smith L, et al. Guidelines for safe practice of stereotactic body (ablative) radiation therapy. J Med Imaging Radiat Oncol 2015;59(5):646–53.

## Friday 28 April, 1:30 PM–3:00 PM

## Image Interpretation in Practice – MI

## Risk‐benefit analysis of a multi‐disciplinary radiographer comment model of care in New South Wales emergency departments

### Ingrid Klobasa,^1,2^ Gary Denham,^3^ Marilyn Baird,^1^ Joshua Best,^4^ Allie Tonks,^5^ Caitlin Tu,^5^ James Abood,^6^ Christopher Jones^7^

#### ^1^Monash University, Melbourne, Australia ^2^NSW Agency for Clinical Innovation, Sydney, Australia ^3^Manning District Hospital, Taree, Australia ^4^Wyong Hospital, Wyong, Australia ^5^Sydney Adventist Hospital, Wahroonga, Australia ^6^Bathurst Hospital, Bathurst, Australia ^7^Broken Hill Hospital, Broken Hill, Australia

**Objectives:** Timely communication of clinically significant medical imaging findings to patient referrers has become a focus of patient safety. Verbal communication is time‐limited, open to misinterpretation and lacks transparency.^1^ The Radiographer Comment and Flag project was developed by the Agency for Clinical Innovation to communicate clinically significant findings to emergency department referrers in real‐time. Five hospitals in New South Wales piloted the project during a 12‐month period for risk–benefit analysis.

**Methods:** Radiographer comments were compared with the radiology report for true positive, false positive or indeterminate classifications. All radiographer errors were reviewed by two auditors and two independent radiologists and risk ratings assigned. Reporting turnaround times and clinically significant cases were collected to identify any potential harm or improved patient care.

**Results:** All Radiographer Comment and Flag cases (n = 1019) were audited for positive predictive values. The pooled average positive predictive values across pilot sites were 0.96 (0.946–0.971, 95% CI). Radiographer error rates ranged from 2.1 to 5.0% (3.4% average). Radiology reports were incidentally found to have a 3.8% discrepancy/error rate. Reporting turnaround times ranged from three minutes to several weeks. Average report turnaround times were 10 and 15 hours at two sites, respectively. Direct benefits were noted for 179 clinically significant cases (17.3%). No significant patient risks were identified.

**Discussion/Conclusion:** The radiographer comment error rate (3.4%) compared well with international reporting error rates (3–5%).^2^ The implementation of the Radiographer Comment and Flag with a multi‐disciplinary healthcare team has shown many patient benefits at ‘point of care’. These benefits outweigh any potential patient risks and allows for earlier medical intervention.

**References**

1. NSW Government. Garling Enquiry. 2008. Final Report of the Special Commission of Inquiry: Acute Care in NSW Public Hospitals, Sydney.
2. Brady AP. Error and discrepancy in radiology: inevitable or avoidable? Insights Imaging 2017;8(1):171–82.

## Championing change: how radiographers are impacting the reduction in diagnostic errors in the emergency department

### Abbie Petts,^1^ Michael Neep^2^

#### ^1^Gold Coast University Hospital, Southport, Australia ^2^Logan Hospital, Meadowbrook, Australia

**Objectives:** This is the first study to compare and combine the radiographic interpretation performance of emergency referrers and radiographers in an emergency department.

**Methods:** A total of 838 radiographic examinations were analysed from 1 August to 24 August 2020. The range of examinations reviewed included both paediatric and adult presentations and included the appendicular and axial skeleton. The emergency referrer and radiographer interpretations for each examination were compared to the radiologist's report. Sensitivity, specificity and accuracy were calculated.^1^ Cohen's Kappa statistic was used to assess the level of agreement between radiographers and referrers with radiologists.^2^

**Results:** The radiographer interpretation demonstrated a mean sensitivity, specificity and accuracy of 80%, 98% and 92%, respectively. The emergency referrer interpretation demonstrated a mean sensitivity, specificity and accuracy of 82%, 95% and 89%, respectively. The highest level of agreement was between the radiographer interpretation and radiologist report (Cohen's Kappa Score = 0.81 ‘almost perfect’) When the radiographer and emergency clinician's interpretations were combined, it yielded a mean sensitivity, specificity and accuracy of 90%, 93% and 92%, respectively.

**Conclusion:** This study demonstrated that with the addition of a radiographer's X‐ray interpretation, an emergency referrer's interpretation can be more accurate than the emergency referrer's interpretation in isolation. This highlights the value of championing change and implementing a system that documents a radiographer's X‐ray interpretation that can complement an emergency referrer's interpretation when a radiologist report is unavailable.

**References**

1. Brealey S, Scally AJ, Thomas NB. Methodological standards in radiographer plain film reading performance studies. Br J Radiol 2002;75(890):107–13.
2. Cohen J. A coefficient of agreement for nominal scales. Educ Psychol Meas 1960;20(1):37–46.

## Shaping the future through education: identification of urgent findings

### Kristal Lee,^1^ Kriscia Tapia,^1^ Mo'ayyad Suleiman,^1^ Catherine Jones,^1,2,3^ Patrick Brennan,^1^ Ernest Ekpo^1^

#### ^1^The University of Sydney, Sydney, Australia ^2^Monash University, Melbourne, Australia ^3^I‐MED Radiology, Brisbane, Australia

The Medical Radiation Practice Board of Australia professional capabilities require radiography, nuclear medicine and radiation therapy graduates to be able to practise independently in CT.^1^ This involves not only the safe and effective operation of the CT system, but also the ability to assess images for diagnostic quality and evaluate them for any urgent or unexpected findings.^1^

In order to provide safe and effective patient care on graduation, medical radiation students need to be educated in abnormality detection of significant and time‐sensitive findings. However, according to the literature, there is a lack of accessible education interventions in image interpretation of urgent findings for medical radiation practitioners^2^ and no validated educational interventions for students.

To address this deficiency, an e‐learning resource using a novel education platform has been developed. Learning modules each containing 10 CT chest examinations have been created with a mix of both normal and abnormal findings. For each examination, students are asked to identify if there are any abnormalities present, rate their confidence in their decision and, where an abnormality is present, assign a level of urgency. Using the test‐enhanced learning strategy, after each module is completed, students are provided with immediate, individual feedback on their sensitivity and specificity scores as well as access to the pre‐assigned truths.

Details of the resource creation will be presented, along with plans for its evaluation and how its implementation will help shape the future of our profession.

**References**

1. Medical Radiation Practice Board of Australia. Professional capabilities for medical radiation practice. 2020. Available at https://www.medicalradiationpracticeboard.gov.au/Registration-Standards/Professional-Capabilities.aspx.
2. Murphy A, Ekpo E, Steffens T, Neep MJ. Radiographic image interpretation by Australian radiographers: a systematic review. J Med Radiat Sci 2019;66(4):269–83.

## A multi‐disciplinary approach to implementing a radiographer preliminary image evaluation system at a tertiary hospital

### Xanthe Keneally^1^

#### ^1^Princess Alexandra Hospital, Brisbane, Australia

The Medical Radiation Practice Board of Australia indicates that radiographers must take timely action on identifying urgent or unexpected findings to ensure appropriate patient management.^1^ This must be documented to support further discussions regarding patient care, in line with the National Safety and Quality Health Service Standards.^2^ It is therefore important that departments have guidelines supporting this process.

There are several barriers to successful radiographer preliminary image evaluation (PIE) system implementation, including inconsistency of guideline use and presence, lack of time and radiologists' resistance,^3^ the latter evidenced in the Royal Australian and New Zealand College of Radiologists' 2018 Position Statement.^4^

There is evidence of Australian departments implementing a range of PIE systems, but not of a multi‐disciplinary approach.^5^ Implementation science reports many interventions to ensure effective guideline implementation, including stakeholder involvement, education of the intents and benefits, and having a quality improvement, performance measuring system.^6^ Healthcare teams are seen as complex adaptive systems, posing further challenges when implementing change.^7^

With this in mind, in 2020, radiographers at the Princess Alexandra Hospital in Brisbane worked alongside consultant radiologists, the clinical education team and team leaders to design and implement a new work unit guideline. The guideline outlines recommended practice for the documentation of significant or unexpected findings identified on X‐ray by radiographers and was implemented to ensure radiographers were complying with the Medical Radiation Practice Board of Australia following removal of their list of medically urgent findings radiographers were expected to identify, as well as anecdotally poor compliance to a previous version of this departmental guideline.

**References**

1. Medical Radiation Practice Board of Australia. Communicating safely – if urgent or unexpected findings are identified. 2019. Available at https://www.medicalradiationpracticeboard.gov.au/Registration-Standards/Professional-Capabilities.aspx.
2. Australian Commission on Safety and Quality in Health Care. Communicating for safety standard. 2022. Available at https://www.safetyandquality.gov.au/standards/nsqhs-standards/communicating-safety-standard.
3. Neep MJ, Steffens TS, Owen R, McPhail SM. Radiographer commenting of trauma radiographs: a survey of the benefits, barriers and enablers to participation in an Australian healthcare setting. J Med Imaging Radiat Oncol 2014;58:431–8.
4. The Royal Australian and New Zealand College of Radiologists. Image Interpretation by radiographers – not the right solution. 2018. Available at https://www.ranzcr.com/college/document-library/image-interpretation-by-radiographers-not-the-right-solution-position-statement.
5. Murphy A, Ekpo E, Steffens T, Neep MJ. Radiographic image interpretation by Australian radiographers: a systematic review. J Med Radiat Sci 2019;66(4):269–83.
6. Peters S, Sukumar K, Blanchard S, et al. Trends in guideline implementation: an updated scoping review. Implement Sci 2022;17(1):50.
7. Pype P, Mertens F, Helewaut F, Krystallidou D. Healthcare teams as complex adaptive systems: understanding team behaviour through team members' perception of interpersonal interaction. BMC Health Serv Res 2018;18:570.

## The effect of clinical information on radiology reporting: a systematic review

### Chelsea Castillo,^1^ Tom Steffens,^1^ Lawrence Sim,^3^ Liam Caffery^2^

#### ^1^Princess Alexandra Hospital, Brisbane, Australia ^2^University of Queensland, Brisbane, Australia ^3^Queensland Health, Brisbane, Australia

**Objectives:** It is common practice for radiologists to use clinical information to assist in the interpretation process.^1^ This systematic review aimed to investigate the effect of clinical information communicated to the reporting radiologist, on the resultant report.

**Methods:** A study protocol was published in the PROSPERO register (CRD42019138509).^2^ Peer‐reviewed studies that investigated a relationship between clinical information and the resultant report were included. Multiple electronic databases were searched, and reference lists from included studies interrogated. Studies were screened for eligibility by two reviewers, with consensus reached by discussion. The Joanna Briggs Institute Critical Appraisal Checklist for Quasi‐Experimental studies was used to assess the quality and risk of bias of each study.^3^

**Results:** Following removal of duplicates, 824 abstracts were screened, 21 studies met the inclusion criteria. A range of imaging modalities was represented, including plain X‐ray (n = 12), CT (n = 5) and MRI (n = 1), with three including two modalities. Study populations ranged from seven to 733. Significance testing used varied greatly and included t‐test, receiver operator characteristic curve, and McNemar's test. The heterogeneity of study designs, outcomes measured and significance testing used precluded meta‐analysis. A narrative synthesis was undertaken to explore the evidence for effect on outcomes.

**Conclusion:** Clinical information communicated to radiologists has an effect on the accuracy, confidence, timeliness and clinical relevance of the resultant report. The range of interventions described suggests clinical information is valued in the reporting process. Further investigation is needed to discover the most reliable method of communicating relevant clinical information to reporting radiologists.

**References**

1. Royal Australian and New Zealand College of Radiologists. Radiodiagnosis Training Program Curriculum. Sydney: The Royal Australian and New Zealand College of Radiologists; 2014.
2. Castillo C, Steffens T, Caffery L, Sim L. The effect of clinical information on radiology reporting: a systematic review. PROSPERO 2019 CRD42019138509 Available at https://www.crd.york.ac.uk/prospero/display_record.php?ID=CRD42019138509.
3. The Joanna Briggs Institute. The Joanna Briggs Institute critical appraisal tools for use in JBI systematic reviews. 2017. Checklist for quasi‐experimental studies (non‐randomised experimental studies). Available at http://joannabriggs.org/research/critical-appraisal-tools.html.

## Analysing false‐positive errors when Australian radiographers use preliminary image evaluation

### Jermayne Takapautolo,^1^ Michael Neep,^1^ Debbie Starkey^1^

#### ^1^Logan Hospital, Meadowbrook, Australia

**Objectives:** Diagnostic errors in the emergency department can have major implications on patient outcomes.^1^ Preliminary image evaluation (PIE) is a brief comment written by a radiographer describing an acute or traumatic pathology on a radiograph. It can be used to complement a referrers radiographic interpretation in the absence of the radiologist report.^2^ Currently no studies exist that involve the analysis of false‐positive errors in PIE. The purpose of this study was to investigate the anatomical regions that cause the most false‐positive errors.

**Methods:** A longitudinal retrospective clinical audit was conducted to determine the diagnostic accuracy of radiographer PIEs over a five‐year period from January 2016 to December 2020.

**Results:** A sample size of 11,090 radiographic examinations were included in the analysis. An overall PIE accuracy of 88.6% and a service accuracy of 94.6%. Foot, ankle and chest regions caused the most false‐positive errors, while ankle, shoulder and elbow caused the most unsure errors. 73.3% of the unsure cases were negative for any pathology when compared to the radiologist report. The paediatric population accounted for 21.3% of false‐positive cases and 33.6% of unsure cases.

**Discussion/Conclusion:** Findings in this study should be used to improve education specific to radiographer image interpretation. Enhancing radiographer image interpretation skills can assist in improving referrer diagnostic accuracy, thus improving patient outcomes.

**References**

1. Spiczka A, Waibel L, Garcia E, et al. Diagnostic accuracy & pathology revised reports: evidence‐based guideline development. Am J Clin Pathol 2020;154(4):124.
2. Neep MJ. The delivery of image interpretation education for radiographers. Queensland University of Technology. [Internet]. 2019 February 5 [cited 2021 July 17]. Available at https://eprints.qut.edu.au/123708/1/Michael_Neep_Thesis.pdf.

## Friday 28 April, 1:30 PM–3:00 PM

## Education – MI

## Entering the professional workplace: ‘it's a shared experience’

### Julie Zhang^1,2^

#### ^1^Princess Alexandra Hospital, Brisbane, Australia ^2^University of Canberra, Bruce, Australia

**Objectives:** Graduate radiographers entering the professional healthcare setting need to orientate and familiarise themselves in a rapidly changing work environment to deliver a high standard of diagnostic imaging services. This study undertook to understand the perspectives of graduate radiographers on their work readiness on entering their new qualified positions.

**Methods:** Ethics clearance was obtained from the university (HREC 2020–4751). A qualitative phenomenological approach was used to collect data from 14 purposively sampled graduate radiographers through individual in‐depth semi‐structured telephone interviews. The rich data were transcribed verbatim and then thematically analysed.

**Results:** New graduate radiographers had varied but shared experiences. Their past influenced their present‐day practice, with many recognising increasing skills and confidence as time went on.

**Discussion:** Participants' work readiness was largely facilitated by clinical work‐integrated learning placements during their university training. These placements exposed radiography students to the realities of the job, which helped accustom them to the differing workflows, protocols, social structures and systems that exist in public and private healthcare settings.^1–3^ Participants' support systems and coping strategies are varied, as unique personal life experiences and individual attitudes and behaviours influence a graduate's capabilities and readiness to work professionally.^4^

**Conclusion:** As new graduates become familiar with their new role and responsibilities, the challenges they face are met with progressive confidence, skills and expertise.

**References**

1. Lundgren S, Lundén M, Andersson BT. Radiography – how do students understand the concept of radiography? Radiography 2014;21(2):e68–73.
2. Bates A, Bates M, Bates L. Preparing students for the professional workplace: who has responsibility for what? Asia‐Pacific Journal of Cooperative Education 2007;8(2):121–9.
3. Hager P, Holland S. Introduction. In: Hager P, Holland S. Graduate attributes, learning and employability. 1st edn. Dordrecht: Springer; 2006.
4. Dames S. The interplay of developmental factors that impact congruence and the ability to thrive among new graduate nurses: a qualitative study of the interplay as students transition to professional practice. Nurse Educ Pract 2019;36:47–53.

## Embracing complexity – lessons learned from delivery of large‐scale bespoke training across diverse professional groups

### Karen Thomas,^1,2^ Daniel Ho^1,2^

#### ^1^Health Support Services – WA, Perth, Australia ^2^Fiona Stanley Hospital, Murdoch, Australia

**Background:** Western Australia's Medical Imaging Replacement Program is replacing its existing AGFA picture archiving and communications system and radiology information system with the Enterprise Medical Imaging Platform (EMIP). The EMIP is a consortium of more than 15 applications. The staff in scope for EMIP applications training included hundreds of medical imaging staff and hospital clinicians who interacted with medical imaging services, across nine metropolitan hospitals. A six‐person training team, consisting of medical imaging clerks and technologists, facilitated the design, development and delivery of training activities and resources, ensuring staff were adequately prepared for the significant change into the future.

**Discussion:** Given the large and diverse group, training methods had to account for variation of training needs across the professions, departments and hospitals, as well as individual learning styles. Training was delivered through a hybrid model of learning, with a combination of e‐learning, online reference materials and face‐to‐face instruction. This approach catered to variations in training needs and adapted to the constant challenges faced, such as staff engagement, workload pressures and COVID‐19. The hybrid model facilitated training participation and commitment, enabling management and staff to confidently embrace the new EMIP solution.

**Conclusion:** Employing an adaptable hybrid training model was pivotal in the efficiency of the EMIP training program. The positive training outcomes highlighted the importance of using a mixture of training artefacts to address the challenges of complex training requirements, as well as activities to cater to different learning styles.

## Undergraduate medical imaging students' experiences and perspectives on professionalism: a qualitative longitudinal study

### Jayanti Gyawali,^1^ Jaime Lee,^2^ Michelle Allen,^3^ Chandra Rekha Makanjee^4^

#### ^1^Qscan Radiology, Canberra, Australia ^2^Austin Health, Victoria, Australia ^3^Logan Hospital, Meadowbrook, Australia ^4^University of Canberra, Bruce, Australia

**Introduction:** Professionalism emphasises humanistic aspects among other attitudes, behaviour, virtues and characteristics.^1^ Professional conduct means conducting yourself responsibly, respectfully, diligently, courteously, and with competence, skill and maturity in alignment with the prescribed professional standards and capabilities as prescribed by the regulatory body.^2^ This highlights the importance of integrating these concepts as early as undertaking their course at the university level.^3^

**Objective:** This study explored undergraduate medical imaging students' experiences, perspectives and understandings of professionalism in medical imaging.

**Methods:** A qualitative longitudinal study was conducted between August 2020 to November 2021 using a convenient sampling technique of 40 participants that enabled acquiring rich insights into their meaning‐making, knowledge and understanding of professionalism. Seven focus group interviews pre‐ and then post‐placement were conducted followed by inductive data analysis and interpretation to thematic saturation.

**Results/Discussion:** Students acquired their knowledge through theory units and undertaking clinical practice that shaped their meaning‐making and understanding of professionalism and the possible implications it could have in practice. Examples shared among others were qualified professionals accustomed to routinised emphasis on task completion with speed. However, the novel students were vocal among others, being honest about making mistakes rather than just focussing on punctuality and physical appearance including the softer skills in terms of being empathetic and compassionate and being culturally safe and sustaining safety culture.

**Conclusion:** This study provided insights into undergraduate medical imaging students' awareness, knowledge and, to some extent, a holistic understanding of professionalism from a practice perspective.

**References**

1. Desai MK, Kapadia JD. Medical professionalism and ethics. J Pharmacol Pharmacother 2022;13(2):113–8.
2. Medical Radiation Practice Board of Australia. Code of conduct. 2021. Available at https://www.medicalradiationpracticeboard.gov.au/Registration-Standards/Code-of-conduct.aspx.
3. Poorchangizi B, Borhani F, Abbaszadeh A, et al. The importance of professional values from nursing students' perspective. BMC Nurs 2019;18:26.

## Embracing change: shaping our student radiographers by embedding critical thinking into the medical imaging curriculum

### Tracey Pieterse^1^

#### ^1^University of Auckland, Auckland, New Zealand

**Background:** In the clinical environment, the ability to think critically is imperative for radiography students to work efficiently as part of a team in an era of rapidly advancing technology.^1^ Students' ability to demonstrate critical thinking skills was studied at a comprehensive university in South Africa in order develop a more explicit curriculum to facilitate this competency.

**Objectives:** To present results of the study as well as guidelines to embed critical thinking skills into the medical imaging curriculum.

**Methods:** The study employed an exploratory descriptive, mixed methods design. Third year radiography students were purposefully selected to participate in this study. The participants' responses to vignettes (in the form of clinical scenarios) were analysed using a Likert scale and action verbs developed for evaluating evidence of critical thinking skills in a radiography context and providing quantitative data. Field notes were made while analysing responses to each vignette, providing qualitative data.

**Results/Discussion:** The findings indicated that most participants in this study demonstrated a minimal ability to think critically in a vignette. This implies that there is a need for curriculum adjustment to embed critical thinking skills in the curriculum. It is well known that critical thinking skills can be taught to students using a range of methods.^2,3^

**Conclusion:** Curriculum development and delivery must be adjusted to embed critical thinking for this skill to be incorporated into the program outcomes.

**References**

1. McInerney J, Baird M. Developing critical practitioners: a review of teaching methods in the bachelor of radiography and medical imaging. Radiography 2016;22(1):e40–53.
2. Weidman B, Salisbury H. Critical thinking in health sciences and how it pertains to sonography education: a review of the literature. J Diagn Med Sonogr 2020;36(3):244–50.
3. Kahlke R, Eva K. Constructing critical thinking in health professional education. Perspect Med Educ 2018;7(3):156–65.

## The self‐reported knowledge, skills and attitudes of Australian diagnostic radiographers towards evidence‐based practice

### Laura Di Michele,^1^ Amani Bell,^1^ Kate Thomson,^1^ Warren Reed^1^

#### ^1^The University of Sydney, Sydney, Australia

The knowledge to practice gap is well documented in diagnostic radiography.^1,2^ Several older Australian and international studies have found radiographers have fewer positive attitudes towards evidence‐based practice than their allied health, nursing and medical counterparts.^3–7^ A recent Australian study found that radiographers did not routinely engage with empirical evidence and were more likely to rely on clinical experience when approaching clinical situations.^8^ However, little literature exists that explores Australian radiographers' knowledge, skills and attitudes when it comes to evidence‐based practice.

This proposed study aims to better understand the current state of evidence‐based practice among Australian radiographers. The Evidence‐Based Practice Questionnaire will be used, which is a reliable and validated tool that assesses healthcare professionals' knowledge, skills and attitudes towards evidence‐based practice and has been used previously in radiography populations.^2^ The survey has three main sections, one that focusses on current practice, one on attitudes and the final section requires participants to self‐rate their knowledge and skills. The survey was distributed in November/December 2022 and the results will be analysed in early 2023. The findings will be discussed during this presentation and will constitute a baseline to progress evidence‐based practice among Australian radiographers.

**References**

1. Ahonen SM, Liikanen E. Radiographers' preconditions for evidence‐based radiography. Radiography 2010;16(3):217–22.
2. Upton D, Upton P. Knowledge and use of evidence‐based practice by allied health and health science professionals in the United Kingdom. J Allied Health 2006;35(3):127–33.
3. Moran K, Davis CA. Pan‐Canadian survey of medical radiation technologist's views towards evidence‐based practice, research, barriers, and enablers. J Med Imaging Radiat Sci 2020;51(1):29–39.
4. Ooi CC, Lee SHE, Soh BP. A survey on the research awareness and readiness among radiographers in Singapore General Hospital (SGH). Radiography 2012;18(4):264–9.
5. Ramazan F, Aarts S, Widdowfield M. Exploring the implementation of evidence‐based optimisation strategies: a qualitative study of the experience of diagnostic radiographers. Radiography 2022;28(3):804–10.
6. Ugwu AC, Udo BE, Ifenatuora JM, Felix EO. Evidence based medical imaging practice in Nigeria: a paradigm or a placebo? Eur J Radiograph 2009;1(4):169–72.
7. Upton P, Scurlock‐Evans L, Stephens D, Upton D. The adoption and implementation of evidence‐based practice (EBP) among allied health professions. Int J Ther Rehabil 2012;19(9):497–503.
8. Rawle M, Pighills A, Mendez D, Dobeli K. Radiographic technique modification and evidence‐based practice: a qualitative study. J Med Radiat Sci 2022; Aug 25. https://doi.org/10.1002/jmrs.616.

## Is our integrity slipping? Implications of academic dishonesty in medical radiation science practice

### Caroline Nabasenja,^1^ Kym Barry,^1^ Clare Singh^1^

#### ^1^Charles Sturt University, Wagga Wagga, Australia

Maintaining the integrity of assessments is an important role of higher education institutions. This is because assessments provide evidence of learner achievement and competency in qualifications conferred by higher education institutions. Due to the COVID‐19 pandemic, higher education institutions rapidly transitioned to remote and online assessments which were marred with potential for increased incidence of academic dishonesty and an explosion in creative ways to do so. Absence of physical invigilation, anxiety among students, easy access to the learning content online, reduced resilience with online learning and academic inexperience in designing in assessments fit for the online environment increased opportunities for cheating, collusion and plagiarism in assessments.^1^

In medical radiation science, academic dishonesty may increase the incidence of professional dishonesty, which has dire consequences for patients, regulatory bodies and higher education institutions. Patient care and safety may be compromised leading to medico‐legal issues, registration of practitioners that do not meet the standards of the profession, loss of reputation for higher education institutions and a culture where academic dishonesty is normalised which then impacts healthcare delivery.^2^

There is a growing need for all stakeholders to be aware of the prevalence of academic dishonesty, measures to mitigate them and potential impact on professional practice.^3^

The aim of the presentation will be to increase awareness of academic dishonesty, discuss the implications on all stakeholders within the medical radiation science profession and steps to mitigate it.

**References**

1. Holden OL, Norris ME, Kuhlmeier VA. Academic integrity in online assessment: a research review. Front Educ 2021;6.
2. McClung EL, Gaberson KB. Academic dishonesty among nursing students: a contemporary view. Nurse Educator 2021;46(2):111–5.
3. Bretag T, Mahmud S, Wallace M, et al. Core elements of exemplary academic integrity policy in Australian higher education. International Journal for Educational Integrity 2011;7(2).

## Friday 28 April, 1:30 PM–3:00 PM

## Advances in Imaging – NM

## PET dose extravasation

### Stephanie Robbie^1,2^

#### ^1^Qld Xray, Toowoomba, Australia ^2^Charles Sturt University, Port Macquarie, Australia

**Objectives:** The aims of this project were to analyse pilot data that had been collected using a quality assurance device (LARA) to establish extravasation rates in positron emission tomography (PET) in the Australian context and understanding the potential impact this may have on the integrity of standard uptake value measurements and patient outcomes.

**Methods:** A single site with a single PET/CT scanner was used to characterise injections using an autoinjector with standardised apparatus, flush volume and infusion rate using 18F–FDG, 68Ga–PSMA and 68Ga–DOTATATE; more reflective of Australian PET facilities. 296 patients with topical application of LARA sensors were retrospectively analysed.

**Results:** Only 1.1% of studies showed evidence of partial dose extravasation. In total, 9.1% were identified to have an injection anomaly (including venous retention). No statistically significant differences were noted across the radiopharmaceuticals for demographic data. While not demonstrating a statistically significant correlation, there were more extravasated doses associated with female patients (P = 0.334), right side (P = 0.372) and hand injections (P = 0.539). Extravasation was independent of dose administered (P = 0.495), the radiopharmaceutical (P = 0.887), who injected the dose (P = 0.343), height (P = 0.438), weight (P = 0.607) or age (P = 0.716). Extravasation was associated with higher glucose levels (P < 0.001), higher t–half (P = 0.019) and higher aUCR10, tc50, aUCR1 and c1 (all P < 0.001).

**Discussion/Conclusion:** Calculation of the standard uptake value and image quality in PET hinges on accurate dose delivery. Extravasation or partial extravasation of the radiopharmaceutical dose can undermine standard uptake value and image quality and contribute to unnecessary imaging.

## Predictors of survival in the PET/CT era

### Paulina Cegla^1^

#### ^1^Greater Poland Cancer Centre, Poznan, Poland

**Aims:** Rapidly growing cancer incidence and mortality worldwide demand novel approaches to enhance precision of diagnostic tools to deliver earlier detection and improved staging.^1,2^ The main objective of this study is to evaluate the predictive value of semi–quantitative PET/CT parameters in various types of cancer.

**Methods:** Retrospectively, several semi–quantitative parameters: metabolic tumour volume (MTV), total lesion glycolysis (TLG), standardised uptake values (SUVs) for primary tumour as well as for metastatic lesions were assessed and correlated with overall survival in cancer patients. In parallel to conventional statistical analysis, there is an increasing role of using neural network analysis.

**Results:** Based on this single centre analysis TLGtotal, SUVmaxtotal and SUVmeantotal were the key features in predicting survival in non–small cell lung cancer. In head and neck squamous cell carcinoma primary tumour, SUVmax, TotalSUV, MTV, TLG and liver parameters: TLRmax and TLRmean had significant influence on overall survival in these group of patients, while in cervical cancer PET–derived parameters: primary tumour TotalSUV, TLG and MTV and whole–tumour parameters: SUVtotal and MTVtotal had substantial impact on overall survival in these group. In non–small cell lung cancer patients, the artificial neural network provided concordant findings in relations to conventional statistical analysis.

**Conclusion:** In the era of new imaging modalities, predictors derived from functional PET/CT imaging might lead to important improvement in assessment of survival in cancer patients. Moreover, neural network analysis might improve PET–derived parameters and increase the role in prediction of overall survival.

**References**

1. Cegla P, Currie G, Wróblewska JP, et al. Influence of semiquantitative [18F]FDG PET and hematological parameters on survival in HNSCC patients using neural network analysis. Pharmaceuticals 2022;15(2):224.
2. Cegla P, Burchardt E, Roszak A, et al. Influence of biological parameters assessed in [18F]FDG PET/CT on overall survival in cervical cancer patients. Clin Nucl Med 2019;44(11):860–3.

## Friday 28 April, 3:30 PM–5:00 PM

## Diversity, equity and inclusivity in MRS

## Coming out or staying in? Navigating diverse sexual and gender identities at work

### Amanda Bolderston^1^

#### ^1^University of Alberta, Edmonton, Canada

Coming out (‘of the closet’) is the process that sexual and gender minority individuals go through as they work to accept their sexual orientation or gender identity and share that identity openly with others. Many sexual and gender minority healthcare professionals experience explicit and implicit bias at work. This may result in them carrying out significant ‘identity work’ to manage who they tell about their sexual orientation/gender identity and in what circumstances. For sexual and gender minority healthcare professionals (including radiation therapists) who feel comfortable enough to be ‘out’ at work there is evidence that they experience higher job satisfaction and are able to offer support to similarly positioned (sexual and gender minority) patients. This patient provider concordance effect has been studied more extensively in other areas^1^ (e.g. race and ethnicity) but is an emerging area in the context of sexual and gender minority patients and healthcare professionals. This presentation will draw on recent qualitative research in this area and explore what it means to be ?out of the closet’ for radiation therapists at work.^2,3^

**References**

1. Kamen CS. Lesbian, gay, bisexual, and transgender (LGBT) survivorship. Semin Oncol Nurs 2018;34(1):52–9.
2. Bolderston A. Coming out or staying in? Disclosure experiences of lesbian and gay radiation therapists in practice. Radiography 2011;27(4):1142–8.
3. Bolderston A. Coming out at work: the rules. J Med Imaging Radiat Sci 2022;53(3):325–7.

## Saturday 29 April, 9:00 AM–10:30 AM

## Varian Award Session

## Radiation therapy toxicities in head and neck cancer – clinical indicators

### Lauren Winkley,^1,2^ Christopher Oldmeadow,^3^ Ngo Tue Le,^1^ Noel Aherne,^1^ Daphne James,^2^ Yolanda Surjan^2^

#### ^1^Mid North Coast Cancer Institute, Port Macquarie, Australia ^2^The University of Newcastle, Callaghan, Australia ^3^Hunter Medical Research Institute, New Lambton Heights, Australia

**Objective:** To investigate the demographic, clinical and treatment factors that correlate with higher grade mucositis, xerostomia and dermatitis toxicity scores following head and neck radiotherapy for patients treated at the Mid North Coast and Northern NSW Cancer Institutes (2011 to 2017).

**Methods:** Following ethics approval, electronic medical records of patients who undertook a course of curative radiation therapy for squamous cell head and neck cancer were reviewed and analysed. Mucositis, xerostomia and dermatitis toxicity scores recorded at baseline, during and post‐treatment was extracted, along with demographic, clinical and treatment data.

**Results:** A total of 225 patients met the study inclusion criteria. Primary tumours of the oral cavity were found to increase the odds of experiencing higher grade acute mucositis by four times (P = 0.0008), whereas patient age, pre‐treatment ECOG status and N stage were found to increase odds of higher‐grade late mucositis. The use of (intensity modulated radiation therapy) and volumetric modulated arc therapy treatment techniques increased the odds of higher acute xerostomia scores. Late xerostomia predictors included ECOG status, degree of surgery, use of intensity‐modulated radiation therapy and primary tumour site. Bolus was found to increase the odds of late dermatitis by 2.5 times (P = 0.238).

**Conclusion:** Clinical, demographic and treatment factors have been identified that increase acute and late mucositis, xerostomia and/or dermatitis in the patient cohort. Results of this retrospective analysis may allow for more specific targeting of early intervention strategies for patients more likely to experience higher grades of toxicity.

## ASMIRT research support services – helping empower local practitioners

### Rachael Beldham‐Collins,^1,2^ Elizabeth Brown^1,3^

#### ^1^ASMIRT Research Committee, Melbourne, Australia ^2^SWRON Westmead and Blacktown Hospitals, Westmead, Australia ^3^Princess Alexandra Hospital, Brisbane, Australia

The Australian Society of Medical Imaging and Radiation Therapy (ASMIRT) has been supporting members' research endeavours for many years: hosting conferences, research workshops, offering research scholarships, dissemination of members' research surveys, participant recruitment invitation circulation and social media promotion. This support can be invaluable; however, many members may be unaware of the services available.

The ASMIRT Research Committee has been working with ASMIRT to formalise and expand the research services offered to the membership and increase their promotion. This has included advocating for statistical support that members may have previously had difficulty accessing to be made available through the Society. This statistical support could include providing ASMIRT members with advice on study design, analysis and reporting of research, quality improvement and quality assurance projects. These initiatives will benefit all members of the Society by promoting the quality of medical radiation practitioner research being conducted within Australia and provide those undertaking projects with the opportunity to increase recruitment and rigour within their projects.

This presentation will outline the current and new research support services ASMIRT offers its membership and how they can be accessed. By increasing the number of support services available and ensuring members are aware of what is on offer, we can increase research capacity and help empower medical radiation practitioner researchers across Australia to be the changing face of medical radiation practitioner research.

## Saturday 29 April, 9:00 AM–10:30 AM

## Targeted Radiation Therapy – RT

## Quantification of motion reduction using pre‐simulation assessment sessions for stereotactic ablative body radiotherapy

### Sophie Duncan^1^

#### ^1^Liverpool Cancer Therapy Centre, Liverpool, Australia

**Objectives:** Pre‐simulation assessment sessions are utilised for the respiratory motion management of patients receiving stereotactic ablative body radiotherapy (SABR). Pre‐simulation assessment sessions determine a patient's eligibility for motion management strategies prior to CT simulation, primarily expiration breath hold and abdominal compression.^1^ This study aimed to determine the effectiveness of pre‐simulation assessment sessions for eligible patients based on diaphragm motion in free breathing versus when using motion management strategies.

**Methods:** Retrospective data on diaphragm motion in FB and elected motion management strategies was collected for 71 patients. Eligible patients were treated between 2018 and 2022 using SABR for liver, kidney, pancreas, adrenal gland or lower lobe lung tumours. Patients underwent pre‐simulation assessment sessions where radiation therapists recorded the diaphragm motion seen on fluoroscopy through three cycles of free breathing versus the elected motion management strategies.

**Results:** Of the 71 patients, 28 were treated with expiration breath hold, 33 with abdominal compression, two with alternate strategies, and 10 were not suitable for motion management strategies. There was a statistically significant difference between the mean of the amplitude of the diaphragm motion when comparing free breathing and expiration breath hold (P = <0.0001) and free breathing and abdominal compression (P = 0.0133). The mean differences in diaphragm motion between free breathing and expiration breath hold/abdominal compression was 16.9 mm (± 8.3 mm) and 6.2 mm (± 6.8 mm), respectively.

**Conclusion:** Pre‐simulation assessment sessions is a useful tool that can be used to shape the future of radiotherapy by selecting the best patient specific motion management strategies for the reduction of tumour motion during SABR treatments. This study will be used to further investigate the dosimetric effects of motion management strategies on target volume reductions, normal tissue sparing, radiation exposure and radiation induced toxicities.

**Reference**

1. Galayini M, Hazem M, Wallis A, et al. OC‐109: implementing a pre‐simulation assessment session for abdominal SABR: respiratory motion management. Radiother Oncol 2019;141.

## A surface guided radiation therapy vs conventional laser‐based set‐up study for thoracic and pelvic patients

### Nick Lau,^1^ Kate Ford,^1^ Elizabeth Claridge Mackonis,^1^ Paul Aston,^1^ Robin Hill^1^

#### ^1^Chris O'Brien Lifehouse, Camperdown, Australia

Surface guided radiation therapy (SGRT) offers accurate and quick set‐up of radiotherapy patients by using real‐time optical scanning with a laser system.^1−3^ Within SGRT, the lasers are continually scanning the patient's position in real‐time instantly and accurately by comparing it to a reference surface image and has the potential to supersede existing set‐up process such as used fixed laser‐based systems. With our recent acquisition of C‐RAD's Catalyst+ HD treatment solution, the study aims to quantify the efficiency and stability gains of an SGRT soft implementation across palliative and radical thoracic and pelvic patients. This involves aligning patients conventionally with the fixed lasers in the transverse, sagittal and coronal plane for straightening, rotations and isocentre placement. When necessary, Catalyst+ HD's colour light projections are also used to actively guide and inform staff on how to position the patient. This is followed by fine‐tuning the patient position by applying automatic couch corrections detected by Catalyst+ before using kV pre‐treatment verification. All data will be stored in our secure database within the Chris O'Brien Lifehouse. The differences in patient set‐up position accuracy between the two processes will be analysed using ordinary least squares linear regression. We hypothesise the magnitude of corrections required during the pre‐treatment verification are reduced with a soft implementation. Recruitment is ongoing for patients into the study.

**References**

1. Kost S, Shah C, Xia P, Guo B. Setup time and positioning accuracy in breast radiation therapy using surface guided radiation therapy. Int J Radiat Oncol Biol Phys 2018;102(3):e481–2.
2. Bäck SÅJ, Ceberg S, Engelholm S, et al. Increased accuracy in reduced time – surface guided RT for hypofractionated prostate cancer patients. In: 2020 ESTRO proffered papers 20 Ensuring precision and accuracy in RT, 2020 Nov 28 – Dec 1; Sweden.
3. Wei W, Ioannides PJ, Sehgal V, Daroui P. Quantifying the impact of optical surface guidance in the treatment of cancers of the head and neck. J Appl Clin Med Phys 2019;21(6):73–82.

## Implementation of palliative adaptive radiation therapy using simulation‐free diagnostic imaging: a radiation therapist perspective

### Toby Lowe,^1^ Shelley Wong,^1^ Alannah Kedja,^1^ Stephanie Roderick,^1^ Brian Porter,^1^ Kylie Grimberg,^1^ John Atyeo,^1^ Leigh Ambrose,^1^ Alexandra Turk,^1^ Isabelle Fent,^1^ Jeremy Booth,^1,2^ Thomas Eade,^1,2^ Sarah Bergamin^1,2^

#### ^1^Northern Sydney Cancer Centre, Sydney, Australia ^2^Northern Clinical School, The University of Sydney, Sydney, Australia ^3^School of Physics, The University of Sydney, Sydney, Australia

**Background:** The Northern Sydney Cancer Centre has developed pathways allowing palliative radiation therapy to be delivered using diagnostic imaging scans, streamlining the clinical process through a simulation‐free pathway. Approximately 800 patients have been treated using this pathway, with approximately 30% of patients ineligible due to anatomical location or inappropriate diagnostic imaging. Implementation of adaptive radiation therapy began in June 2019 and has been adopted for treatment sites including bladder, rectum, gynaecological, and head and neck using Varian's Ethos. We present on the building of planning templates and protocols for Ethos driven palliative radiation therapy.

**Methods:** Using the Ethos planning and treatment emulator, previously treated diagnostic datasets were used to retrospectively plan and treat in the offline workspace. These were evaluated to test the online reproducibility in the treatment workspace for various anatomical sites for palliative radiation therapy.

**Results:** Since implementation we have treated 13 patients over a range of anatomical sites including bladder, rectum, abdomen and thorax. The online planning time has reduced over these 13 patients from 23 to 17 minutes, with a median of 20 minutes.

**Conclusion:** Introduction of adaptive planning on diagnostic datasets has allowed the Northern Sydney Cancer Centre to build a range of templates for multiple anatomical sites. Progression of simulation‐free adaptive radiation therapy will aim to increase the inclusion of anatomical sites that are currently being excluded, which will further streamline the palliative radiation therapy pathway.

## Evaluating the impact of contour changes on reduced arc VMAT breast dosimetry

### Wilson Cheung,^1^ Brock Lamprecht^1^

#### ^1^Princess Alexandra Hospital, Brisbane, Australia

**Objectives:** The reduced arc volumetric modulated arc therapy (VMAT) breast technique offers the ability to lower organs at risk dose while providing comparable therapeutic efficacy when compared to traditional VMAT breast techniques.^1^ However, there is a need to investigate the effect of contour changes on plan quality.^2^ The purpose of this study was to evaluate the effect of contour changes on planned dosimetry in breast cancer patients treated with the reduced arc VMAT technique.

**Methods:** 20 reduced arc VMAT plans receiving 42.5 Gy in 16 fractions were assessed; 16 left‐sided and four right‐sided breast patients. The medial and lateral halves of the breast were defined by two clip boxes with contour expansions of 0.5 cm, 0.8 cm and 1 cm generated from the external contour to replicate possible tissue swelling. All treatment beam parameters were collected. The effect of contour change variables on tumour volume coverage and organs at risk dose were evaluated.

**Results:** Medial breast expansions of 0.8 cm and 1 cm demonstrated the greatest effect on 95% tumour volume coverage with 65% and 30% of plans, respectively, achieving this (mean = 95.78, IQR = 94.59–97.12, mean = 93.56, IQR = 91.8–96.04) None of the contour expansions showed any clinically significant impact on the organs at risk dose.

**Discussion/Conclusion:** The results of this study suggest that when tissue swelling exceeds 0.5 cm within the treatment area there may be an impact on planning target volume coverage. The extent of this impact may be dependent on several beam and patient related factors. Further investigation is required to determine when replanning intervention is required.

**References**

1. Yu P, Wu C, Nien H, et al. Tangent‐based volumetric modulated arc therapy for advanced left breast cancer. Radiat Oncol 2018;13:236.
2. Lamprecht B, Muscat E, Harding A, et al. Comparison of whole breast dosimetry techniques – from 3DCRT to VMAT and the impact on heart and surrounding tissues. J Med Radiat Sci 2022;69(1):98–107.

## Monitoring lung motion: an evaluation of markerless tumour tracking software for lung SBRT

### Maiko Crispin,^1^ Danielle Chrystall,^1^ Maegan Gargett,^1,2^ Abdella Ahmed,^1^ Nick Hindley,^3^ Jonathan Hindmarsh,^3^ Marco Mueller,^3^ Paul Keall,^3^ Dasantha Jayamaynne,^1^ Sarah Bergamin,^1^ Jeremy Booth^1,4^

#### ^1^Northern Sydney Cancer Centre, Royal North Shore Hospital, St Leonards, Australia ^2^School of Health Sciences, The University of Sydney, Sydney, Australia ^3^ACRF Image X Institute, The University of Sydney, Sydney, Australia ^4^School of Physics, The University of Sydney, Sydney, Australia

**Objectives:** 4D‐CT is essential when delivering ablative doses to lung tumours,^1^ however previous data has shown that 4D‐CT can under‐represent lung motion.^2^ An observational lung stereotactic ablative body radiotherapy trial, VALKIM, is underway for validation of a novel markerless image guidance technology. We report preliminary feasibility of implementing markerless tumour tracking (MTT) software^3^ as a non‐invasive alternative to marker‐based tracking for motion management.

**Methods:** The first two patients enrolled in the VALKIM trial had fiducial markers implanted at the treatment site, to act as a ground truth for evaluating the accuracy of MTT. Patients receive current standard of care, with the addition of kilovoltage X‐ray images acquired continuously during treatment delivery to enable MTT. The accuracy (mean difference) and precision (1 standard deviation) between markerless and marker‐based tracking was assessed.

**Results:** Results for patient 1 show that MTT was accurate within 5 mm and precise with 4 mm for all fractions in all directions, compared to the marker centroid. Results for patient 2 show that MTT was accurate within 3 mm for all fractions in all directions compared to the marker centroid. Precision was 3 mm in the left–right and anterior–posterior directions, however exceeded 8 mm in the superior–inferior direction for 7/8 fractions.

**Discussion:** A non‐invasive motion monitoring method for lung stereotactic ablative body radiotherapy patients has been demonstrated on a standardly equipped linear accelerator. Preliminary results on the accuracy of MTT are promising; further patient recruitment will allow stratification of ideal candidates and facilitate further development of the software.


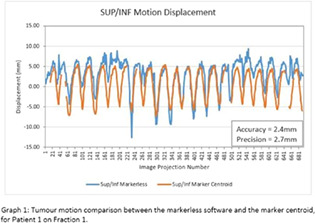


**References**

1. Keall PJ, Mageras GS, Balter JM, et al. The management of respiratory motion in radiation oncology. Report of AAPM Task Group 76. Med Phys 2006;33(10):3874–900.
2. Steiner E, Shieh CC, Caillet V, et al. Both four‐dimensional computed tomography and four‐dimensional cone beam computed tomography under‐predict lung target motion during radiotherapy. Radiother Oncol 2019;135:65–73.
3. Mueller M, Zolfaghari R, Briggs A, et al. The first prospective implementation of markerless lung target tracking in an experimental quality assurance procedure on a standard linear accelerator. Phys Med Biol 2020;65(2):025008.

## Intrafraction motion in prostate SBRT: can CBCT soft tissue matching be used without fiducial markers?

### Linda Bell,^1^ Alannah Kejda,^1^ Thomas Eade,^1,2^ George Hruby,^1,2^ Andrew Kneebone^1,2^

#### ^1^Northern Sydney Cancer Centre, St Leonards, Australia ^2^The University of Sydney, St Leonards, Australia

**Objectives:** A systematic review supports stereotactic body radiotherapy (SBRT) for prostate cancer as an appropriate treatment.^1^ SBRT commonly uses fiducial markers for intrafraction motion detection however not all patients are eligible for marker insertion. This study aimed to compare mid‐treatment cone beam computed tomography (MT‐CBCT) to triggered imaging for intrafraction motion correction in prostate SBRT.

**Methods:** Intrafraction imaging of 165 patients treated on the SBRT arm of the OPTIMAL trial (NCT03386045) were reviewed.^2^ The planning target volume margin was 5 mm and 3 mm posteriorly. 79 patients with fiducial markers underwent triggered imaging, acquired at 12 second intervals, and 86 patients without fiducial markers received MT‐CBCT between treatment arcs. The suitability of MT‐CBCT was compared to triggered imaging by recording magnitude and timing of intrafraction couch shifts.

**Results:** The median couch shift was less than 1 mm in each direction, regardless of imaging modality. No shifts were recorded in 63.4% of triggered imaging fractions, compared to 16.9% with MT‐CBCT. Most triggered shifts occurred during arc 1, while most MT‐CBCT shifts occurred between arc 1 and 2. The percentage of shifts exceeding the planning target volume for triggered and MT‐CBCT methods were 10.0% and 12.7% in anterior–posterior, 2.9% and 1.8% in superior–inferior, and 0.5% and 0.9% in the left–right direction, respectively. Analysis of couch shifts per two‐minute time intervals is shown in Figure 1.

**Conclusion:** Minimal clinically significant shifts were detected prior to four minutes, suggesting that MT‐CBCT may capture intrafraction motion if acquired at a minimum interval of four minutes after pre‐treatment imaging.


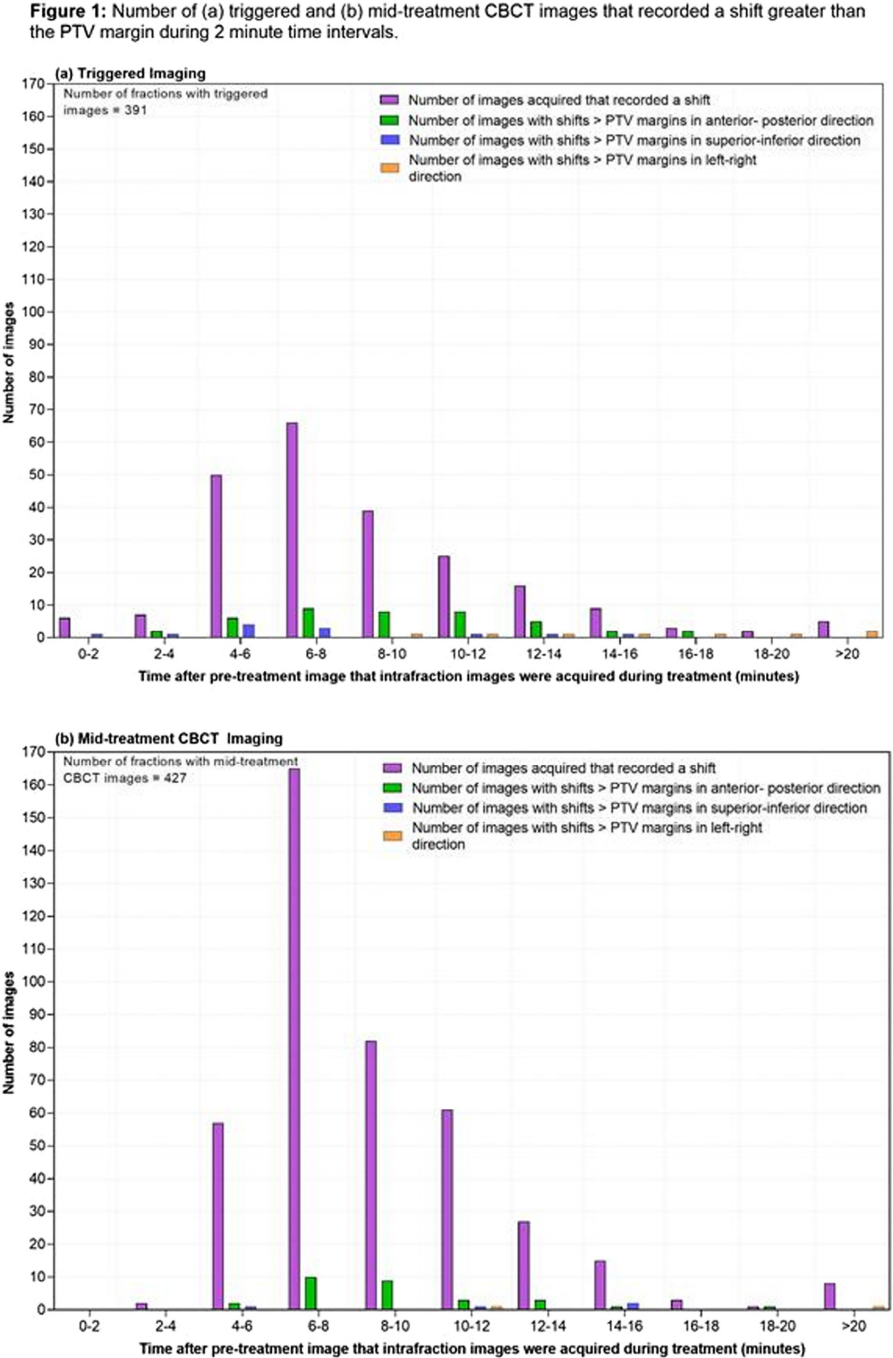


**References**

1. Jackson WC, Silva J, Hartman HE, et al. Stereotactic body radiation therapy for localised prostate cancer: a systematic review and meta‐analysis of over 6000 patients treated on prospective studies. Int J Radiat Oncol Biol Phys 2019;104(4):778–89.
2. U.S. National Library of Medicine ClinicalTrails.gov. The Optimal Prostate Study, 2017. Available at https://clinicaltrials.gov/ct2/show/NCT03386045.

## Saturday 29 April, 9:00 AM–10:30 AM

## Enabling the CT Radiographer – MI

## Novel metal artefact reduction of large implants in CT: a systematic review and network meta‐analysis

### Katya Amadita,^1^ Frances Gray,^1^ Erin Gee, Yobelli Jimenez^1^

#### ^1^The University of Sydney, Sydney, Australia

**Objectives:** When patients with metallic joint prostheses undergo CT imaging, artefacts are produced that may obscure clinical data. Clinically, metal artefact reduction (MAR) methods, aimed to address this problem, can vary in image quality preservation. The aims of this study were to review novel MAR methods targeting large metal artefacts in fan‐beam CT, and to assess their comparative effectiveness artefact reduction while preserving image quality.

**Methods:** This review was conducted within PRISMA guidelines, with an electronic search of five databases. Journal articles published in the English language between 2016 and 2021 were eligible. Data extraction and quality assessment was undertaken independently by two reviewers using pre‐established tools. A pairwise and network meta‐analysis was conducted using results from large metals and all‐sized metals, respectively, due to data availability. Results ineligible for meta‐analysis was synthesised narratively.

**Results:** The 26 included studies explored 34 novel MAR methods: proposing 17 algorithmic and nine machine learning methods, with eight novel comparators and a pooled sample size of 6415. The pairwise meta‐analysis found machine learning methods outperformed baseline reconstructions. The network meta‐analysis found DeStreakNet was the most effective in noise reduction (SMD 1.99, 95% CI –2.25 to 6.22) and image quality (SMD 2.61, 95% CI 0.27 to 4.96). Computation time varied greatly.

**Conclusion:** Despite the low‐level evidence, machine learning MAR methods show promise as feasible tools to reduce metal artefacts while preserving image quality in CT. Further research exploring the diagnostic accuracy and computational complexity of novel MAR methods is recommended before clinical implementation.

## Use of radiographer commenting in CT to improve patient safety

### Allie Tonks,^1^ Caitlin Tu^1^

#### ^1^Sydney Adventist Hospital, Sydney, Australia

The practice of radiographer commenting to alert clinical staff of significant findings on both X‐ray and CT images at the time of acquisition aligns with legal minimum practice standards articulated by the Medical Radiation Practice Board of Australia.^1^ It also addresses professional standards including the National Safety and Quality Health Service Standards and the Diagnostic Imaging Accreditation Scheme Standards.^2,3^ While several clinical sites have radiographer commenting systems in place for general X‐ray examinations, the use of commenting in CT is still emerging. The present study was conducted at a hospital using commenting to alert referrers to significant findings in X‐ray, CT and MRI studies. In the first nine months of data collection, 38 comments were made on CT examinations, representing 18% of all comments. Positive predictive value for CT comments was 0.97. On average, comments were available within five minutes of study completion, and an average of two days and two hours prior to comprehensive, gold‐standard radiologist reports. The most alerted exam types were CT brain, CT pulmonary angiogram and CT abdomen.

This presentation will explore the experience of CT commenting, including case examples and their influence on patient management. Overall, radiographer comments are being utilised to alert clinically urgent conditions demonstrated on CT, which enables referrers to prioritise care, follow‐up with radiologists and expedite treatment to improve patient safety.

**References**

1. Medical Radiation Practice Board of Australia. Professional capabilities for medical radiation practitioners. Available at https://www.medicalradiationpracticeboard.gov.au/Registration-Standards/Professional-Capabilities.aspx.
2. Australian Commission on Safety and Quality in Healthcare. Communicating for safety standard. 2022. Available at https://www.safetyandquality.gov.au/standards/nsqhs-standards/communicating-safety-standard.
3. Australian Commission on Safety and Quality in Healthcare. Diagnostic imaging accreditation scheme standards. Australian Commission on Safety and Quality in Healthcare; 2016. Available at https://www.safetyandquality.gov.au/standards/diagnostic-imaging/diagnostic-imaging-accreditation-scheme-standards.

## Image quality assessment of low‐dose whole‐body CT skeletal surveys for non‐accidental injury

### Edel Doyle^1,2^

#### ^1^Lumus Imaging, Melbourne, Australia ^2^Monash University, Melbourne, Australia

**Background:** With advances in low‐dose CT technology, it has been proposed that a CT skeletal survey could replace a skeletal survey X‐ray series in the investigation of non‐accidental injury.^1^

**Objective:** The aim was to perform a phantom study using whole body CT skeletal survey to establish the threshold below which image quality became undiagnostic.

**Methods:** A paediatric non‐accidental injury phantom was scanned using two different CT scanners at a range of dose levels. These datasets were screened to eliminate any undiagnostic scans as the fracture sites were known. The diagnostic data sets were then reviewed by paediatric radiologists using specific criteria in the DetectEDx software. The effective radiation doses for the diagnostic CT scans were calculated using NCI Dose software. The radiation risks were then estimated using the BEIR VII report.

**Results/Conclusion:** Results and Conclusion to be presented at the ASMIRT 2023 Conference.

**Reference**

1. Lawson M, Tully J, Ditchfield M, Kuganesan A, Badawy MK. Using computed tomography skeletal surveys to evaluate for occult bony injury in suspected non‐accidental injury cases – a preliminary experience. J Med Imaging Radiat Oncol 2022;66(1):41–8.

## Radiographers' knowledge, attitudes and practice of infection prevention and control in the CT suite

### Dania Abu Awwad,^1^ Suzanne Hill,^1^ Sarah Lewis,^1^ Yobelli Jimenez^1^

#### ^1^The University of Sydney, Camperdown, Australia

**Introduction:** Healthcare‐associated infection risk is multi‐faceted in the CT environment where technology, high throughput and varied procedures are common. The purpose of this study was to discover a baseline of knowledge, attitudes and practice of infection prevention and control standard precautions of radiographers working in CT.

**Methods:** A survey was developed, and distributed via email, with 62 CT radiographers (as of mid‐November) completing the survey, 47% with more than 10 years CT experience and 48% from public hospitals. Knowledge, attitudes and practice questions were developed from the Australian Guidelines for the Prevention and Control of Infection in Healthcare^1^ and included knowledge questions (correct/incorrect) and attitudes and practice questions (5‐point Likert scale). Correlations between knowledge, attitudes and practice were compared using Spearman's correlation.

**Results:** Preliminary findings revealed that of the 11 knowledge benchmark questions, participants scored between 55% and 100% correctly, with an average score of 82% (9/11 questions). Approximately 57% of radiographer participants had IPC training in the past year. There was a strong positive correlation between attitudes and practice (rho = 0.54), with a moderate positive correlation between knowledge and attitudes (rho = 0.48). While 100% of participants agreed that CT equipment and patients are sources of infections, 25% did not always applied standard precautions.

**Discussion:** Data collection is ongoing and final survey numbers are expected to grow. Preliminary results indicate there is a strong correlation between the infection prevention and control attitudes of CT radiographers and positive workplace practice, and knowledge and attitudes. While overall infection prevention and control knowledge was strong, it was only moderately correlated with sound practices.

**Reference**

1. National Health and Medical Research Council. Australian guidelines for the prevention and control of infection in healthcare. Canberra: NHMRC; 2019.

## IMPACT requests: shaping the future of CT requesting

### Chelsea Castillo,^1^ Tom Steffens,^1^ Georgia Livesay,^1^ Lawrence Sim,^3^ Liam Caffery^2^

#### ^1^Princess Alexandra Hospital, Brisbane, Australia ^2^University of Queensland, Brisbane, Australia ^3^Queensland Health, Brisbane, Australia

**Objectives:** Inadequate clinical information in medical imaging requests negatively affects the clinical relevance of imaging performed and the quality of resultant radiology reports. Currently, there are no published Australian guidelines on what constitutes adequate clinical information in CT requests. This study aimed to determine specific items of clinical information radiologists require in CT requests for acute chest, abdomen and blunt trauma examinations, to support optimal reporting.

**Methods:** A panel of 24 CT‐reporting consultant radiologists participated in this e‐Delphi consensus study. Panellists undertook multiple online survey rounds of open‐ended, dichotomous and Likert scale questions, receiving feedback following each. Round 1 responses formulated lists for each CT examination. Round 2 set a threshold of 80% agreement after dichotomous scoring. Round 3 accepted items which averaged four or more on a 5‐point Likert scale. Round 4 required panellists to rank items within the aggregated, accepted lists, based on panellists' perceived level of usefulness.

**Results:** The large numbers of round 1 items (chest: 101, abdomen: 76, blunt trauma: 80) were rationalised and grouped into categories to facilitate efficiency during subsequent rounds; 23 chest, 24 abdomen and 17 blunt trauma items met the 80% agreement threshold in round 2. Items below threshold were included in round 3; numbering 44, 19 and 23 for chest, abdomen and blunt trauma, respectively. Through the e‐Delphi process, we formulated clinical information three criteria standards.

**Conclusion:** The developed standards will guide Australian referrers in providing adequate clinical information in CT requests, to support optimal reporting, diagnosis and treatment.

## Theoretical investigation of the upslope of contrast curve as a predictor of optimal contrast enhancement

### Kelly Chu^1^

#### ^1^The University of Sydney, Sydney, Australia

**Objectives:** Current bolus tracking techniques are affected by interpatient variations that may give rise to sub‐optimal contrast enhancement. This study proposes a new approach by using the upslope of the contrast curve as a predictor of CT scan delay. This study aims to investigate whether the upslope can theoretically and accurately predict the CT trigger time for optimal contrast enhancement.

**Methods:** A retrospective analysis of 62 CT brain perfusion scans were performed using the proposed technique. Raw patient data were fitted using a gamma variate function to simulate clinically relevant perfusion curves,^1^ enabling the control of the stochastic noise, sampling frequency and injection rate. Two upslope‐prediction approaches, the golden ratio and the 80/20 rule, were developed and evaluated using 10 cases with different sampling frequencies. Each sampling frequency was assessed using three statistically independent noisy realisations. The performance of the proposed protocol, in terms of prediction accuracy and prediction gain, was tested for generalisability using another 17 unseen cases.

**Results:** The proposed prediction technique using the golden ratio approach has higher prediction accuracy and lower prediction gain than the 80/20 rule approach. There is a substantial reduction in peak enhancement variability from 38.9% to 13.9% within the 17 test cases. The prediction gain of the proposed protocol is approximately 4.9 s (SD = 1.7) with a prediction error of 41.8 HU (SD = 10.0).

**Conclusions:** The novel slope‐based prediction technique can potentially reduce contrast enhancement variability under defined conditions. Future work will include the evaluation of the proposed technique against other existing predicting methods.

**Reference**

1. Madsen MT. A simplified formulation of the gamma variate function. Phys Med Biol 1992;37(7):1597–600.

## Saturday 29 April, 9:00 AM–10:30 AM

## The Breast – MI

## “When will they invent something better” A review of dedicated cone beam breast computed tomography

### Kelly Spuur^1^

#### ^1^Charles Sturt University, Wagga Wagga, Australia

From its beginnings in 1913, mammography, like all radiography modalities, has undergone significant technological advancement. The gold standard for breast cancer diagnosis, mammography has transitioned from film screen acquisition to high‐fidelity full‐field digital mammography and digital breast tomosynthesis. Dedicated population‐based screening programs using mammography have reduced morbidity and mortality from breast cancer worldwide.^1^ Despite its success, mammography has inherent issues, including the need for manual handling of the breast for imaging, breast compression, which can cause pain and is a known barrier for women to undergo imaging, and the fact that neither conventional 2D nor 3D breast imaging is able to image the entire breast.^2^ These issues have driven the need for the development of new technology to answer the most commonly asked question by women: “When will they invent something better?”

This presentation provides an overview of the future of breast imaging using dedicated 3D cone beam breast computed tomography (CBBCT) (see Fig. 1). Commercially available and Food and Drug Administration approved, CBBCT requires no radiographer manipulation of the breast, uses no compression and, most importantly, can image all the breast. Radiation dose is comparable to conventional mammography. The spatial resolution enables the identification of 2 mm lesions and 200‐μm calcification.

Importantly, for women whose cultural or religious beliefs do not allow touching of the breast by others, the adoption of CBBCT will enable more inclusive imaging regimens. For all women, the removal of the need for compression can only mean more compassionate imaging and strong demand for this new technology.


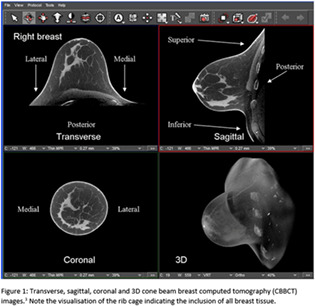


**References**

1. Molassiotis A, Tyrovolas S, Giné‐Vázquez I, et al. Organised breast cancer screening not only reduces mortality from breast cancer but also significantly decreases disability‐adjusted life years: analysis of the Global Burden of Disease Study and screening programme availability in 130 countries. ESMO Open 2021;6(3):100111.
2. Whelehan P, Evans A, Wells M, MacGillivray S. The effect of mammography pain on repeat participation in breast cancer screening: a systematic review. Breast 2013;22(4):389–94.

## Mammography: exploring the imaging of women with cardiac implantable electronic devices in Australia

### Joanne Fitzgerald,^1^ Kelly Spuur,^1^ Clare Singh,^1^ Christopher Hayre^1,2^

#### ^1^Charles Sturt University, Wagga Wagga, Australia ^2^University of Exeter, Exeter, United Kingdom

**Objectives:** The number of women in Australia with pacemakers and other cardiac implanted electronic devices (CIEDs) is increasing, as is the number of women presenting for mammography with these devices.^1,2^ This study explored radiographers' device knowledge, perspectives and approaches to mammographic imaging of women with CIEDs.

**Methods:** Australian Society of Medical Imaging and Radiation Therapy members were invited to complete an online survey. Analysis was undertaken using SPSS™, NVivo and the chi‐squared test. Ethics approval was granted through the Charles Sturt University Human Research Ethics Committee.

**Results:** There were 220 valid responses; most felt confident identifying pacemakers (95%, n = 191/201) and implantable cardioverter defibrillators (86.1%, n = 174/202). Only 5.9% (n = 13) of consent forms specifically asked if the patient had a CIED; 20.1% (n = 44/219) reported a site protocol; 46.5% (n = 20/43) stated protocol use depended on patient presentation; 64.3% (n = 27/42) of screening respondents reported ‘always’ feeling confident of views required compared to 25.5%, (n = 14/55) diagnostic, (P < 0.001). Most radiographers were afraid of damaging devices during compression (85.1%, n = 167/196); 28.8% (n = 15/52) of the diagnostic workforce were ‘always’ afraid; 49.2% (n = 97/197) felt they lacked information to respond confidently to patient concerns and questions. Open‐ended responses reflected the importance of professional judgement.

**Discussion/Conclusion:** This research has documented uncertainty and inconsistency in practice among radiographers and between BreastScreen and diagnostic settings. Results highlight the urgent need for dedicated radiographer education and the development of guidelines and policies to support best practice imaging of women with a CIED.

**References**

1. Australian Institute of Health and Welfare. Cardiovascular disease in women Cat. no. CDK 15. Canberra: Australian Government; 2021. Available at https://www.aihw.gov.au/getmedia/25915222-41b7-4697-b877-8a8d7daaa836/aihw-cdk-15.pdf.aspx?inline = true.
2. Mond HG, Crozier I. The Australian and New Zealand cardiac implantable electronic device survey: calendar year 2017. Heart Lung Circ 2019;28(4):560–6.

## Breast cancer screening of BRCA gene patients at a tertiary Australian hospital: an audit

### Nicole Hambleton,^1^ Ahn Nguyen,^1^ Karen Doheny,^1^ Karen Dobeli^1^

#### ^1^Royal Brisbane and Women's Hospital, Herston, Australia

**Objectives:** The aim of this retrospective audit was to compare the screening pathway for BRCA patients at the Royal Brisbane and Women's Hospital to international BRCA screening guidelines.

**Methods:** Institutional ethics board approval was obtained. Breast examinations performed for BRCA screening over a four‐year prior were identified. The number and type of examinations per patient were calculated and the modality by which a breast cancer diagnosis was made was recorded. Patient records were reviewed to obtain information regarding commencement/cessation of screening.

**Results:** 144 patients were included, 1064 screening exams were performed. Ultrasound was most frequently used (n = 462), followed by MRI (n = 345) and mammography (n = 257). Patients had an average of 1.7 examinations per year (range = 0.8–2.0). Four breast cancers were identified on MRI, two on ultrasound and one on mammography. MRI was contraindicated in the two patients whose breast cancers were diagnosed via ultrasound. 118 patients were having ongoing screening. Screening had ceased for 26 patients due to breast cancer diagnosis (n = 7) or bilateral prophylactic mastectomy (n = 19).

**Discussion/Conclusion:** Ultrasound is the predominant screening modality for BRCA patients at our institution. This is contrary to most international guidelines, which recommend annual MRI from an early age, annual mammography later in life and limited use of ultrasound due to its comparatively low sensitivity. Our study supports the use of MRI as the primary screening modality. Investigation into reasons for the heavy reliance on ultrasound is warranted.

## The state of mammographic image quality analysis in Australia

### Kelly Spuur^1^

#### ^1^Charles Sturt University, Wagga Wagga, Australia

Breast cancer affects 2.3 million women worldwide, with one in eight Australian women diagnosed annually.^1^ The effectiveness of mammography in both the diagnostic and screening settings is dependent on the production of high‐quality images, with image evaluation systems used to monitor positioning and radiographer performance.

Longstanding requirements in Australia for image quality analysis of the two routine breast views, the craniocaudal and mediolateral oblique, are described by BreastScreen Australia in their National Accreditation Standards, and in the Royal Australian and New Zealand College of Radiologists Mammography Quality Assurance Program manual.^2,3^ Radiographers undertake prospective image quality evaluation in both settings, with the key difference being that diagnostic mammograms are determined to be acceptable or repeatable; in contrast, the PGMI (Perfect, Good, Moderate, and Inadequate) image evaluation systems used by BreastScreen Australia requires the assignment of a grade.

The introduction of digital acquisition in mammography has enabled the development of benchmarked, automated image quality assessment software variably modelled on the PGMI image evaluation systems. Currently used in both the diagnostic and screening settings, this new approach to image quality evaluation has created a third tier of image quality analysis not currently captured in the standards of the accrediting bodies.

There is now an urgent need for a review of image quality evaluation in Australia and for standards to incorporate the evidence base enabled through digital analysis. Standardisation of image quality assessment is required so that all radiographers can be equally benchmarked, and all images assessed in the same way.

**References**

1. Australian Institute of Health and Welfare. BreastScreen Australia monitoring report 2022. Canberra: AIHW; 2022.
2. BreastScreen Australia. National accreditation standards. BreastScreen Quality Improvement Program. Canberra: BreastScreen Australia; 2019.
3. American College of Radiology. Mammography quality control manual. Reston, VA: American College of Radiology; 1999.

## Saturday 29 April, 11:00 AM–12:00 PM

## Building a Resilient and Responsive Workforce

## Workforce resilience: a manager's perspective

### Leighton Rogan^1^

#### ^1^I‐MED Radiology Network, Wagga Wagga, Australia

Resilience, the ability to tolerate or recover quickly from difficulties. For medical imaging technologists, the COVID‐19 pandemic has presented numerous challenges that tested the character of our workforce. In the two years prior to the pandemic the nuclear medicine profession had struggled through significant product supply disruption. Both these events have led to the prominence of resilience being used as a workforce descriptor. Survival of a series of stressful events is not resilience and caution must be exercised to ensure those at risk are identified and supported. Workforce resilience is a dynamic characteristic and needs to be nurtured. When managers support and assist their team to become resilient, organisations rebound and recover from the impacts of the difficult situation.

This presentation outlines what workforce resilience is, the rewards of workforce resilience, how workforce resilience benefits the individual worker and as a manager within a medical imaging environment what role we play in the resilience of our workforce.

## Scheduled medicines in medical radiation science

### Alan Malbon^1^

#### ^1^Australian Society for Medical Imaging and Radiation Therapy, Melbourne, Australia

This presentation follows the Victorian Coronial Inquiry into the death of Peta Hickey (F/43) following a routine CT scan with an injection of contrast media.^1^ The subsequent coronial recommendations will have profound ramifications for all medical radiation science practitioners.

The Australian Society for Medical Imaging and Radiation Therapy (ASMIRT) has held discussions with the Medical Radiation Practice Board of Australia to discuss these recommendations to ascertain the viability of a project which supports and endorses prescribing, the supply and administration of scheduled medicines pertaining directly to medical radiation science practitioners.

ASMIRT has called for a national working party to develop a body of work that encapsulates the potential prescription, supply and administration of scheduled medicines which directly pertain to medical radiation science. It should also include pharmaceutical agents to also aid in the event of an anaphylactic reaction. This will form the basis of a submission to the Australian Health Ministers Advisory Council seeking approval to endorse health practitioners under Section 94 of the National Law.

ASMIRT has engaged with overseas medical radiation societies in the United Kingdom, New Zealand and Canada to gain an insight into their workplace practices and protocols in this area.

This presentation will outline the coroner's report released in 2021, which recommended the Medical Radiation Practice Board of Australia review capabilities to ensure general inclusion of recognising and responding to an emergency and administering treatments as capabilities, as well as an understanding of international workplace practices and protocols.

This thought‐provoking presentation will engage the profession to consider key practices in their own everyday workplace.

**Reference**

1. McGregor S, Hickey R, Ellis F, Tseng G. In: The Coroners Court of Victoria at Melbourne [Internet]. 2021. Available at https://www.coronerscourt.vic.gov.au/sites/default/files/2021–11/2019%202,336%20Hickey%20-%20Form%2037.pdf.

## Saturday 29 April, 11:00 AM–12:00 PM

## PET/CT – NM/MI

## 18F‐fluoroestradiol PET imaging in estrogen receptor positive breast cancer

### Tina M Buehner^1,2^

#### ^1^GE Health Care, Chicago, United States ^2^Rush University, Chicago, United States

In the United States, one in eight women will be diagnosed with breast cancer in their lifetime. In Australia, one in seven women will be diagnosed with breast cancer, being the second most commonly diagnosed malignancy in both the United States and Australia.^1,2^

Molecular imaging in advanced breast cancer is commonly performed using 18F‐fluorodeoxyglucose positron emission tomography (PET) to evaluate patients for metastatic disease. Metabolic tumour uptake of 18F‐fluorodeoxyglucose is based on the demand for increased glucose metabolism by malignant tumour cells but may have low cellular uptake predominantly in indolent, or low‐glycolytic malignancies, such as invasive lobular carcinomas.

18F‐fluoroestradiol is a synthetic oestrogen analogue and the first receptor‐targeted radiotracer developed in the United States and approved by the Food and Drug Administration in May 2020 for the detection of oestrogen receptor positive lesions with PET imaging as an adjunct to biopsy in recurrent/metastatic breast cancer. Approximately 80% of all breast cancers in women are oestrogen receptor positive, as such these patients are often treated with endocrine therapy. Hormone receptors may, however, change over time and with treatment, therefore understanding the receptor expression particularly in the metastatic setting is vital for management of this patient population.

Immunohistochemistry via biopsy is the gold standard for hormone receptor determination. However, in May 2022 the largest prospective clinical trial to date established 18F‐fluoroestradiol validity compared to immunohistochemistry in detecting oestrogen receptor expression. The study found 18F‐fluoroestradiol PET to be a viable alternative when biopsy is unable to be performed.^3^

**References**

1. American Cancer Society. Breast cancer key statistics. 2022. Available at https://www.cancer.org/research/cancer‐facts‐statistics/all‐cancer‐facts‐figures/cancer‐facts‐figures‐2022.html.
2. National Breast Cancer Foundation. Breast cancer statistics. 2022. Available at https://nbcf.org.au/about‐breast‐cancer/breast‐cancer‐stats.
3. van Geel JJL, Boers J, Elias SG, et al on behalf of the IMPACT‐Metastatic Breast Consortium. Clinical validity of 16α‐[18F]fluoro‐17β‐estradiol positron emission tomography/computed tomography to assess estrogen receptor status in newly diagnosed metastatic breast cancer. J Clin Oncol 2022;40(31):3642–52.

## Saturday 29 April, 11:00 AM–12:00 PM

## Evidence Based Practice and Research

## Qualitative research: interpreting quality

### Amanda Bolderston^1^

#### ^1^University of Alberta, Edmonton, Canada

Quality criteria for qualitative research are an important, but debated, topic. As in quantitative work, broad concepts of validity and relevance can be used but the concepts and processes vary according to the different underpinning goals of qualitative research. The term ‘trustworthiness’ has been widely recognised as a way to discuss quality since first being conceptualised by Lincoln and Guba.^1^

This presentation will focus on how to design and carry out trustworthy qualitative research using an eight‐point framework developed by Tracy that consists of: a worthy topic, rich rigor, sincerity, credibility, resonance, significant contribution, ethics and meaningful coherence.^2^

**References**

1. Lincoln YS, Guba EG. Naturalistic inquiry. Newbury Park, CA: Sage; 1985.
2. Tracy SJ. Qualitative quality: eight “big‐tent” criteria for excellent qualitative research. Qualitative Inquiry 2021;16(10):837–51.

## Bridging the gap between haphazard care and evidence‐based care in cancer

### Geoff Delaney^1^

#### ^1^Liverpool Hospital, Liverpool, Australia

**Introduction**: Deviation from evidence‐based care can occur for many reasons. Some valid reasons are where patient co‐morbidities preclude the planned therapy or patients decline advice about evidence‐based care after being adequately informed by their clinicians. We also know that there are situations where treatment is not delivered with a valid reason – this is referred to as unwarranted variation in care.

**Results**: We have shown that although an evidence‐based model suggests that 48% of the Australian population should receive radiotherapy at least once, only 28% currently do so.^1^ We have also shown that reductions in local control and survival for cancer patients will occur if evidence‐based care is not received.^2,3^

Factors that impact on radiotherapy utilisation include lower socio‐economic status and geographic distance from a treatment facility.^4^ This has led to initiatives to improve access to radiotherapy including reducing waiting times, reducing fractionation schedules and funding of smaller radiotherapy facilities in rural centres to reduce travel time. However, using retrospective data to reduce variations in care based on individual clinician decision‐making has been largely unsuccessful due to slowness of the analytical process; so, when data are reported back to clinicians the response is usually that the data are old and not in keeping with changes in practice.

This presentation will review the evidence to date on unwarranted variation in cancer care and propose a near‐real time decision support system as a possible contribution to improving the link between evidence‐based care and patient decision making.^5^

**References**

1. Barton MB, Jacob S, Shafiq J, et al. Estimating the demand for radiotherapy from the evidence: a review of changes from 2003 to 2012. Radiother Oncol 2014;112(1):140–4.
2. Hanna T, Shafiq J, Delaney GP, Barton MB. The population benefit of evidence‐based radiotherapy programs: 5‐year local control and overall survival benefits. Radiother Oncol 2018;126:191–7.
3. Batumalai V, Shafiq J, Gabriel G, et al. Impact of radiotherapy underutilization measured by survival shortfall, years of potential life lost and disability adjusted life years lost. Asia Pac J Clin Oncol 2017;13:169–70.
4. Gabriel G, Barton M, Delaney GP. The effect of travel distance on radiotherapy utilization in NSW and ACT. Radiother Oncol 2015;117(2):386–9.
5. Delaney GP, Barton MB. Great expectations or waiting for Godot? Time for development of a near real‐time national reporting system of radiotherapy utilisation. J Med Imaging Radiat Oncol 2022;66(6):826–9.

## Saturday 29 April, 1:30 PM–3:00 PM

## Adaptive RT

## Surveying radiation oncologists and radiation therapists to shape our future in online adaptive radiation therapy

### Meegan Shepherd,^1,2^ Minkyo (Christina) Suh,^1^ Alexander Podreka,^1^ Carlito Coronel,^1^ Claire King,^1^ Sarah Bergamin,^1^ Brian Porter,^1^ John Atyeo^1,2^

#### ^1^Northern Sydney Cancer Centre, St Leonards, Australia ^2^Monash University, Clayton, Australia

**Introduction:** There has been increasing interest in online adaptive radiation therapy (oART), whereby the patient contours and plan can be reshaped at the time of daily treatment, providing increased accuracy and superior treatment plans to patients.^1^ It is widely accepted that oART delivery is limited due to radiation oncologist (RO) availability.^2^ This study sought to explore perceptions of confidence, accuracy and attitudes of Northern Sydney Cancer Centre ROs and radiation therapists (RTs) on pelvic organ contouring and training that will ultimately up‐skill RTs to lead oART.

**Method:** All ROs sub‐specialising in pelvis radiotherapy and all RTs at the Northern Sydney Cancer Centre completed pre‐ and post‐training surveys. RO and RT surveys sought perceptions of accuracy and confidence in RT contouring, rating of importance for contouring pelvis organs for training, preferred training methods, RO willingness to train RTs, demographic data and previous contouring training.

**Results:** Six ROs and 33 RTs completed the initial survey. A high degree of confidence exists among ROs for RTs contouring most pelvis organs such as bladder, sigmoid and rectum. Moderate confidence for uterus, prostate, seminal vesicles and urethra, which mirrored RT results. ROs unanimously expressed their keenness to train and assess RTs.

**Conclusion:** The agreeance for further training and willingness of ROs to contribute to up‐skilling RTs, provides a platform for our institution to continue designing RT‐led oART. Having confidence that stake‐holder groups understand existing limitations, and the value of collaboration in delivering an effective training plan, allows us to synthesise a unified approach to championing this significant change for our patients benefit.

**References**

1. Sonke JJ, Aznar M, Rasch C. Adaptive radiotherapy for anatomical changes. Semin Radiat Oncol 2019;29(3):245–57.
2. Shepherd M, Graham S, Ward A, et al. Pathway for radiation therapists online advanced adapter training and credentialing. Tech Innov Patient Support Radiat Oncol 2021;20:54–60.

## Virtual training for radiation therapists in pelvis contouring: shaping accuracy capabilities for online adaptive RT

### Meegan Shepherd,^1,2^ Alexander Podreka,^1^ Carlito Coronel,^1^ Minkyo (Christina) Suh,^1^ Brian Porter,^1^ Sarah Bergamin,^1^ Claire King,^1^ John Atyeo^1,2^

#### ^1^Northern Sydney Cancer Centre, St Leonards, Australia ^2^Monash University, Clayton, Australia

**Objectives:** Traditional treatment planning workflows allow for radiation therapists (RTs) and radiation oncologists (ROs) to contour pelvic organs under minimal time pressure.^1^ Recent implementation of online adaptive radiation therapy (oART) at our facility requires RTs to contour organs normally contoured by ROs, decreasing the likely need for ROs at the treatment console,^2^ while helping facilitate increased delivery, efficiency and oART benefits to patients.^3^ We investigate if a RO virtual workshop improves RT contouring accuracy in pelvis organs.

**Method:** Baseline assessments of contouring accuracy on five pelvis organs were undertaken by RTs using Proknow contour accuracy. RTs initial contours were assessed against RO gold standard contours using Dice similarity coefficient (DSC). RTs then completed virtual RO workshops for each organ, supplemented with organ anatomy guidelines. RTs then completed second round contours on new image datasets. Assessments between initial DSC and post‐training DSC were evaluated.

**Results:** 33 RTs participated in baseline assessments of bladder, rectum, bowel bag, sigmoid and uterus contouring. Initial results indicate excellent DSC scores for bladder, rectum and sigmoid. Bowel bag contours showed most variation and worst overlap DSC. Uterus contours were excluded from initial analysis due to incorrect gold standard. Second phase contour analysis is currently in progress and results available 2023.

**Conclusion:** Presently, RT initial contouring of some pelvic organs shows excellent agreement against a single gold standard contour on CT images, relative to the DSC less than 0.7 recommended in the literature for RT/RO contour agreement. The RO virtual training intervention and supplementary information is currently under evaluation.

**References**

1. Arculeo S, Miglietta E, Nava F, et al. The emerging role of radiation therapists in the contouring of organs at risk in radiotherapy: analysis of inter‐observer variability with radiation oncologists for the chest and upper abdomen. Ecancermedicalscience 2020;14:996.
2. McNair HA, Joyce E, O'Gara G, et al. Radiographer‐led online image guided adaptive radiotherapy: a qualitative investigation of the therapeutic radiographer role. Radiography 2021;27(4):1085–93.
3. Sibolt P, Andersson LM, Calmels L, et al. Clinical implementation of artificial intelligence‐driven cone‐beam computed tomography‐guided online adaptive radiotherapy in the pelvic region. Phys Imaging Radiat Oncol 2021;17:1–7.

## The impact of patient positioning lasers on setup accuracy for MR‐linac radiotherapy

### Louise Hogan,^1^ Michael Jameson,^2^ David Crawford,^2^ Katrina Biggerstaff,^1^ Maddison Picton,^2^ Charles Tran,^2^ Laura McKenzie,^2^ Tommy Liang,^1^ Madeline Carr,^2^ Jeremiah de Leon,^2^ Hendrick Tan,^1^ Jasmine Thum,^1^ Vikneswary Batumalai^2^

#### ^1^GenesisCare Murdoch, Perth, Australia ^2^GenesisCare St Vincent's, Sydney, Australia

**Objective:** Traditionally, the first step in patient alignment has relied on lasers to reduce set‐up errors. The introduction of online adaptive treatment on the MR‐linac may see the lasers as redundant. This study aimed to compare differences in set‐up errors measured with and without the use of lasers in patients treated on the MR‐linac.

**Methods:** 20 patients received stereotactic ablative body radiotherapy in five fractions to the abdominal region on the MR‐linac at two different centres were retrospectively selected. 10 patients were positioned using laser and 10 patients without lasers. Daily MR‐linac scans were registered to the reference CT plan or previous adapted MR plan, with the 3D isocentre shifts recorded for analysis. Differences in daily positioning errors were examined using paired student's t‐tests. The mean (M) and standard deviation (SD) of the set‐up errors were calculated. The SD of population M expressed the systematic error (Σ), and the root mean square of the SD describes the random error (σ).

**Results:** Differences in daily errors between the two groups were statistically significant in the superior–inferior direction (P = 0.0001) but not in the left–right (P = 0.8) or anterior–posterior (P = 0.08) directions. In the superior–inferior direction, the systematic and random errors were higher in the group without laser by 0.74 cm and 0.41 cm, respectively (see Table 1).

**Conclusion:** Significant large variation was noted in the superior–inferior direction for patients positioned without laser. While these offsets can be corrected using the MR‐linac adaptive workflow, minimising the errors with laser for patient positioning may reduce the overall treatment time.


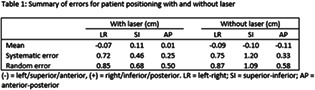


## A collaborative approach to developing an advanced practice curriculum in adaptive radiation therapy

### Kristie Matthews,^1,2^ Caroline Wright,^1,2^ John Atyeo,^1,3^ Nigel Anderson^1,4^

#### ^1^Monash University, Melbourne, Australia ^2^Peter MacCallum Cancer Centre, Melbourne, Australia ^3^Northern Sydney Cancer Centre, Sydney, Australia ^4^Austin Health, Melbourne, Australia

The implementation of adaptive radiation therapy (ART) technologies is changing the paradigm of treatment delivery, whereby it is possible to modify the intended radiation therapy plan to best suit the patient anatomy of the day.^1^ It is essential that the radiation therapist has the advanced skills, knowledge and aptitude to be an active and safe contributor to ART treatment delivery, where responsibility for timely and accurate clinical decision making and daily plan approval adds complexity to usual procedures.^2^ A flexible and responsive advanced education program is required to enable the radiation therapist to lead ART procedures, in the context of continually evolving ART technology and practice applications.

Subsequently, a collaboration was established between Monash University, Northern Sydney Cancer Centre, and Olivia Newton‐John Cancer Wellness and Research Centre in late 2020 to develop and implement an advanced practice curriculum for ART. The key objectives of the collaboration were to:

- Identify the advanced capabilities required of an ART‐radiation therapist beyond current practice expectations.
- Map content delivery and assessment strategies to allow demonstration of the advanced capabilities.
- Establish a postgraduate course pathway and flexible clinical training framework to enable achievement of the advanced capabilities.

The Master of Advanced Radiation Therapy Practice ART pathway was implemented in March 2022. The curriculum was designed to meet the needs of academic and inter‐professional clinical stakeholders and includes advanced learning content and a clinical school. This outcome demonstrates that sustained active collaboration between universities and clinical centres can enable a proactive educative response to new technology implementation.

**References**

1. Lim‐Reinders S, Keller BM, Al‐Ward S, Sahgal A, Kim A. Online adaptive radiation therapy. Int J Radiat Oncol Biol Phys 2017;99(4):994–1003.
2. Green OL, Henke LE, Hugo GD. Practical clinical workflows for online and offline adaptive radiation therapy. Semin Radiat Oncol 2019;29(3):219–227.

## Dose escalated stereotactic ablative radiotherapy of pancreas on MR‐linac

### David Crawford,^1^ Vikneswary Batumalai,^1,2^ Maddison Picton,^1^ Claire Pagulayan,^1^ Charles Tran,^1^ Laura Mckenzie,^1^ Urszula Jelen,^1^ Nicole Dunkerley,^1^ Tania Twentyman,^1^ Michael Jemeson,^1,2^ Jeremy deLeon^1^

#### ^1^GenesisCare, Darlinghurst, Australia ^2^The University of New South Wales, Sydney, Australia

**Objective:** Standard radiotherapy delivering 40–60 Gy (1.8–2 Gy per fraction) add minimal to no survival benefit for patients with unresectable locally advanced pancreatic cancer.^1^ Magnetic resonance‐guided adaptive radiotherapy (MRgART) allows delivery of stereotactic ablative body radiotherapy (SABR) effectively with improved overall survival.^2^ We present our early experience of dose escalated SABR of pancreas treated with MRgART.

**Methods:** 10 patients with locally advanced pancreatic cancer were treated with MRgART on the Unity MR‐Linac (December 2021 to August 2022). The clinical target volume was defined as gross tumour volume and para‐aortic nodal regions covering coeliac axis to superior mesenteric artery and was uniformly expanded by 3 mm to create the planning target volume. Planning risk volume involved a 3–5 mm expansion of organs at risk. Any overlapping portion of the planning target volume by the planning risk volumes was constrained to 33 Gy to meet organs at risk constraints, and the remainder (planning target volume high) was dose‐escalated to 50 Gy.

**Results:** The median age was 65 years (range = 50–80 years), and six patients were male. The clinical target volume D99 coverage was ideal/acceptable in 88% of total fractions, while the planning target volume D99 coverage was ideal/acceptable in 72% fractions. Ablative dose was delivered to most target volumes as demonstrated by the median planning target volume high D90 dose of 43.4 Gy (see Table 1). The median time from patient set‐up to treatment delivery completion was 50 minutes (range = 29–67 minutes).

**Conclusion:** Dose escalated SABR treated with MRgART is dosimetrically feasible and tolerable. Analysis of larger patient cohort with follow‐up including toxicity and patient reported outcomes is planned.


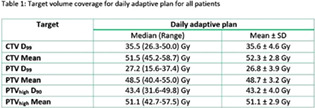


**References**

1. Hammel P, et al. Effect of chemoradiotherapy vs chemotherapy on survival in patients with locally advanced pancreatic cancer controlled after 4 months of gemcitabine with or with‐out erlotinib: the LAP07 randomised clinical trial. JAMA 2016;315(17):1844–53.
2. Hassanzadeh C, et al. Ablative five‐fraction stereotactic body radiation therapy for inoperable pancreatic cancer using online MR‐guided adaptation. Adv Radiat Oncol 2021;6(1):100506.

## MR‐guided adaptive stereotactic ablative body radiotherapy for liver cancers

### Michael Velec^1^

#### ^1^Princess Margaret Cancer Centre, Toronto, Canada

**Aim**: Liver stereotactic ablative body radiotherapy (SABR) is challenging given the motion and deformation of critical anatomy that is poorly visualised on standard cone‐beam CT. To overcome this, the aim is to describe a liver SABR workflow using online adaptive SABR with an MR‐linac.

**Methods**: Patients were imaged under 2D fluoroscopy on a C‐arm linac to quantify motion under active breath‐hold or an abdominal compression belt. Those with motion less than 15 mm using compression underwent 1.5T MR‐linac simulation to determine tolerance and suitability of non‐contrast 3D MR and 2D cine‐MR. Triphasic CT‐sim and 3T MR‐sim aided in gross tumour volume delineation. MR‐based planning applied 5 mm planning target volume around internal target volume and used 8–12 IMRT fields. Daily online treatment involved plan re‐contouring and re‐optimisation (i.e. ‘adapt‐to‐shape’ workflow) by a team of two therapists (one dual‐certified in MR), one physicist and one oncologist.

**Results**: Thirty‐five patients underwent MR‐linac simulation, and 12 did not proceed due to motion (five), imaging (two), patient tolerance (one), technical fault (one) or clinical reasons (three). Twenty‐three patients (eight primary liver cancer, 15 metastases) with 1–4 gross tumour volumes were treated with 16–24 Gy/1 fraction, 24–39 Gy/3 fractions, or 27.5–45 Gy/5 fractions. Optimal MR sequences were patient specific, either respiratory‐triggered at exhale or time‐averaged T2w 3D MR for planning, and T1w, T2w or balanced contrast 2D cine‐MR for motion monitoring. All 83 fractions were delivered ‘adapt‐to‐shape’ and a mean patient on‐couch time of 66.6 minutes.

**Conclusion**: Liver cancer SABR with MR‐linac‐based online adaptation was implemented with a patient‐specific workflow based on tumour motion and optimal imaging.

## Saturday 29 April, 1:30 PM–3:00 PM

## Championing Change in MI

## Radiographer response to medical emergencies in the radiology department: a scoping review

### Caitlin Tu^1,2^

#### ^1^Sydney Adventist Hospital, Wahroonga, Australia ^2^Flinders University, Bedford Park, Australia

**Introduction:** The radiology department is a dynamic multi‐disciplinary area of the hospital with high patient throughput and various patient presentations and acuity.^1^ A patient's clinical condition can deteriorate while in the radiology department requiring appropriate, timely response from radiographers, who are often the first to respond to these events.^2^ This scoping review aims to explore the educational approaches/interventions of radiographers responding to emergencies, the outcomes and associated methods used to assess outcomes.

**Method:** The search strategy aimed to locate published evidence on Medline, Scopus and Proquest databases using participant, context, concept framework. Three independent reviewers undertook title and abstract screening, full text screening and data extraction of selected sources in Covidence.

**Results:** 4573 studies underwent title and abstract screening with 4556 deemed irrelevant; 17 studies underwent full text screening with six excluded; 11 studies were included for data extraction.

**Discussion:** Simulation‐based education, educational modules and questionnaires were identified as the main educational approaches/interventions using pre‐test, post‐test and cross‐sectional research design. These educational approaches have improved radiographer knowledge and confidence in CPR and anaphylaxis response. Many gaps within the literature were found including cohort study research design or radiographers as the sole participant group, lack of qualitative data or studies undertaken in Australia.

**Conclusion:** In conclusion, radiographer knowledge and confidence in certain aspects of emergency response are improved with simulation‐based education, educational modules and questionnaires. Identified gaps in the literature present an opportunity for further research shaping the future of radiographers responding to medical emergencies.

**References**

1. Wallin A, Gustafsson M, Carlsson A, Lunden M. Radiographers' experience of risk for patient safety incidents in the radiology department. J Clin Nurs 2019;28(7–8):1125–34.
2. O'Neill JM, McBride KD. Cardiopulmonary resuscitation and contrast media reactions in a radiology department. Clin Radiol 2001;56(4):321–5.

## Championing change: exploring the prevalence of debriefs in radiography departments following a critical incident

### Simran Sampath Kumar,^1^ Michael Neep^1^

#### ^1^Logan Hospital, Meadowbrook, Australia

**Objectives:** There is an urgent need for more focus on mental health and wellbeing among healthcare workers in allied health; particularly radiography departments involved in trauma and intensive care unit imaging. The challenges of the pandemic have highlighted the inadequacies in mental health support and training in hospitals and the lack of literature regarding this topic, particularly in radiography departments, suggests there is a gap. This review aimed to explore the prevalence and necessity of debriefing tools in radiography departments associated with trauma and intensive care unit imaging.

**Methods:** A search of terms associated with radiography, critical incidents, trauma and debriefs was conducted using Google Scholar and PubMed.

**Results:** There were no articles pertaining specifically to radiography departments. Five articles were deemed eligible for inclusion as they targeted debriefing tools in emergency and intensive care unit settings. The literature suggests debriefing tools are an asset to healthcare workers as it benefits quality improvement and improves staff morale and support, which directly impacts the delivery of quality patient care.^1–4^ Differences between the types of debriefing were analysed.

**Discussion/Conclusion:** This review of the literature highlighted a gap in evidence pertaining to debriefs in radiography departments following a critical incident and suggests that early intervention tools such as hot debriefs and mental health training have the potential to provide greater support in radiography departments and thus, improve secondary outcomes to deliver imaging services safely and efficiently. Future research is warranted to explore the misconception of the impact of trauma on radiographers.

**References**

1. Bateman ME, Hammer R, Byrne A, et al. Death cafés for prevention of burnout in intensive care unit employees: study protocol for a randomised controlled trial (STOPTHEBURN). Trials 2020;21(1):1–9.
2. Walker CA, McGregor L, Taylor C, Robinson S. STOP5: a hot debrief model for resuscitation cases in the emergency department. Clin Exper Emerg Med 2020;7(4):259.
3. Brazil V, Williams J. How to lead a hot debrief in the emergency department. Emergency Medicine Australasia 2021;33(5):925–7.
4. Gilmartin S, Martin L, Kenny S, Callanan I, Salter N. Promoting hot debriefing in an emergency department. BMJ Open Quality 2020;9(3):e000913.

## The value‐adding radiographer of the future

### Tom Steffens,^1^ Adam Steward^2^

#### ^1^Princess Alexandra Hospital, Brisbane, Australia ^2^Western Health, Footscray, Australia

Value‐based health care was first proposed by Porter in 2010.^1^ The concept involves optimising all health inputs to maximise quality, long term, sustainable outcomes for patients and enhance their experiences. Value‐based health care maximises value within the healthcare system by analysing each element of the value chain to increase efficiency, minimise waste and eliminate activity that does not improve patient outcomes or enhance their experience.^2^ Several Australian health jurisdictions, such as NSW Health, have begun focussing more on value‐based models of care rather than more traditional activity‐based models.^3^

Due to their unique position in the health care value chain, radiographers are ideally placed to influence patient outcomes. Through receiving communication from referrers and patients, caring for and communicating directly with patients, optimising imaging and passing vital information on to radiologists, radiographers who adopt a value‐adding mindset can significantly improve outcomes for their patients. Radiographic practice in Australia is regulated by the Medical Radiation Practice Board of Australia (MRPBA). The MRPBA professional practice capabilities guide radiographers in various domains of their practice, including professionalism, interprofessional collaboration, the use of best‐practice evidence and patient care. The overall goal of these capabilities is to ensure radiographers practise safely and competently.^4^

This presentation will:

1. Outline the principles of value‐based health care as it pertains to medical imaging
2. Explain the medical imaging value chain and radiographers' role as a key component
3. Describe specifically how radiographers can add value to their patients and shape the future of the healthcare system, with respect to various MRPBA professional practice domains.

**References**

1. Porter ME. What is value in health care? N Engl J Med 2010;363(26):2477–81.
2. Porter ME, Teisberg EO. Redefining health care: creating value‐based competition on results. Harvard Business School Press; 2006.
3. New South Wales Government. 2020. Leading better value care. Available at https://www.health.nsw.gov.au/Value/lbvc/Pages/default.aspx.
4. Medical Radiation Practice Board of Australia. 2020. Professional capabilities for medical radiation practitioners. Available at https://www.medicalradiationpracticeboard.gov.au/Registration-Standards/Professional-Capabilities.aspx.

## Seeing the bigger picture: embracing modern pain science to enhance care in the imaging department

### Sophie Shephard,^1,2^ Kate Dahlenburg,^3^ Michael Neep^3,4^

#### ^1^Vive Pain Rehabilitation, Wagga Wagga, Australia ^2^Pain Revolution (University of South Australia), Adelaide, Australia ^3^Logan Hospital, Meadowbrook, Australia ^4^Queensland University of Technology, Brisbane, Australia

Scientific understandings of pain have changed dramatically in recent years,^1,2^ yet it seems radiographers are being left out of these conversations, despite frequently encountering patients experiencing pain.^3^ Embracing modern pain science offers an opportunity to enhance current and future practice, and facilitate optimal patient care.

Pain is a complex phenomenon arising from the interplay of biological, psychological and social factors.^2^ It is now known that pain can occur in the absence of tissue pathology,^1^ the degree of tissue pathology cannot reliably predict pain,^1,4^ and that ‘pathological’ imaging findings are highly prevalent in pain‐free cohorts.^5,6^ Clearly, pain is more complicated than once thought. This is important knowledge for radiographers, as patients may experience severe acute or chronic pain even in cases with no evidence of injury. Patients with chronic pain, particularly those without a clear pathoanatomical cause, have reported feeling stigmatised by health professionals, even accused of ‘faking’ or ‘drug‐seeking’.^7^ Clinical encounters themselves have potential to increase distress, or promote trust and safety, both of which may directly influence pain.^1,8^ Indeed, pain severity experienced during mammograms has been linked to radiographer attitudes, opinions and communication.^9^

This presentation will provide a summary of modern pain science, before discussing potential implications and applications within radiography practice to achieve optimal person‐centred care. Avenues for future research will be highlighted, including exploration of radiographers' current understanding of pain, and the impact of enhanced pain knowledge on clinical outcomes and patient experiences in the imaging department.

**References**

1. Moseley GL. Reconceptualising pain according to modern pain science. Phys Ther Rev 20,071;12(3):169–78.
2. Melzack R, Katz J. Pain. Interdiscip Rev Cogn Sci 2013;4(1):1–15.
3. Cordell WH, Keene KK, Giles BK, et al. The high prevalence of pain in emergency medical care. Am J Emerg Med 2002;20(3):165–9.
4. Kasch R, Truthmann J, Hancock MJ, et al. Association of lumbar MRI findings with current and future back pain in a population‐based cohort study. Spine 2022;47(3):201–11.
5. Brinjikji W, Luetmer PH, Comstock B, et al. Systematic literature review of imaging features of spinal degeneration in asymptomatic populations. AJNR Am J Neuroradiol 2015;36(4):811–6.
6. Horga LM, Hirschmann AC, Henckel J, et al. Prevalence of abnormal findings in 230 knees of asymptomatic adults using 3.0 T MRI. Skeletal Radiol 2020;49(7):1099–107.
7. Nicola M, Correia H, Ditchburn G, Drummond P. Invalidation of chronic pain: a thematic analysis of pain narratives. Disabil Rehabil 2021;43(6):861–9.
8. Mistiaen P, van Osch M, van Vliet L, et al. The effect of patient–practitioner communication on pain: a systematic review. Eur J Pain 2016;20(5):675–88.
9. Goethem MV, Mortelmans D, Bruyninckx E, et al. Influence of the radiographer on the pain felt during mammography. Eur Radiol 2003;13(10):2384–9.

## You're going to do what? Bridging the gap between wards and medical imaging

### Stephen Lacey^1,2^

#### ^1^The Royal Children's Hospital, Parkville, Australia ^2^The University of Melbourne, Parkville, Australia

**Introduction:** In the delivery of patient care, we each have roles to play. Understanding other's roles is vital to minimising communication failures and ensuring positive patient care is delivered.^1^ Procedures across imaging modalities within one department can be complex and diverse. Communicating our requirements of these procedures to ward staff is essential to delivering universal care.

**Method:** We surveyed ward nursing and medical staff to determine their level of understanding of medical imaging procedures, allowing them to express any concerns that they had with the associated modalities. We simultaneously surveyed imaging staff to identify communication and patient preparation issues of ward patients. Following the surveys, the intent was to provide education to ward staff specific to each modality addressing the concerns and providing a consistent, communicative approach.

**Results:** In line with literature, a lack of understanding of patient requirements for imaging procedures, who to contact in imaging and nursing handover were among the major issues identified by both ward and imaging staff.^1,2^ A series of educational modules for each modality was created, including clinical information specific to the modality, contact details and operational workflow. This was made available to all staff involved in patient clinical care via the internal learning management system.

**Conclusion:** Module availability is currently in its infancy with future plans to evaluate their effectiveness. It is hypothesised that these modules will improve the understanding of imaging procedures and workflow, thereby bridging the communication gap across the multiple specialties involved in patient care and improving workflow efficiency.

**References**

1. Marlow C. Communication is key. Aust Nurs Midwifery J 2017;24(8):26.
2. Thomas JWM, Schultz TJ, Hannaford N, Runciman WB. Failures in transition: learning from incidents relating to clinical handover in acute care. J Healthcare Qual 2013;35(3):49–56.

## Saturday 29 April, 1:30 PM–3:00 PM

## Student Award – RT

## An evaluation of ankle and foot bolus in paediatric modulated arc total body irradiation

### Hannah Hering,^1^ Beth Effeney,^2^ Carole Brady,^2^ Catriona Hargrave^1,2^

#### ^1^Queensland University of Technology, Brisbane, Australia ^2^Princess Alexandra Hospital, Brisbane, Australia

**Objectives:** To evaluate the role of ankle and foot bolus in achieving dose uniformity in paediatric patients undergoing modulated arc total body irradiation treatment and to identify patient factors that may negate or warrant its use for future practice.

**Methods:** This ethics‐approved retrospective study included 20 paediatric patients across a range of ages who received modulated arc total body irradiation treatment utilising ankle and foot bolus. Dose metrics were obtained from the original bolus plan, a second plan without bolus, and a third adapted plan without bolus attempting to achieve equal dosimetry to the original plan. Data collected included maximum dose to a defined volume (1 cm^3^), the volume receiving 85%, 90%, 100%, 110% and 115% of the reference dose for each sub‐region of the ankle and foot, and patient height, weight and age at time of treatment. Data were analysed using descriptive statistics, box plots and scatter plots and Spearman's correlation test.

**Results:** All three plans demonstrated clinically insignificant variation in maximum dose. There was clinically significant variation in coverage of each subregion receiving 90% of the prescribed dose (V90). For the ankle and foot regions in the bolus plans, V90 was on average more than 92%, while in plans without bolus, an average reduction of 23% coverage was seen in the toes. Spearman's correlation test and scatter plots suggest height has the strongest correlation to ankle and foot dose.

**Discussion/Conclusion:** This study validated the continued use of ankle and foot bolus to achieve primary dosimetric goals for paediatric modulated arc total body irradiation treatments, particularly V90 coverage across all ages.

## Breathing ‘right’: a novel comparison of coached respiratory motion

### Bethany McCullough,^1,2^ David Willis,^2^ Julie Burbery,^1^ Catriona Hargrave^1,3^

#### ^1^Queensland University of Technology, Brisbane, Australia ^2^Sunshine Coast University Hospital, Birtinya, Australia ^3^Princess Alexandra Hospital, Brisbane, Australia

**Objectives:** Currently the breath hold is used in modulated radiation therapy for right‐sided breast cancer to mitigate the interplay effect. This study aimed to assess the novel use of a 3D surface scanner to measure respiratory motion during coaching with the view of reducing reliance on breath hold technologies for right‐sided breast radiation therapy.

**Methods:** Institutional ethics was obtained to recruit 10 healthy volunteers who were coached through different breathing techniques including free breathing, breath hold, deep breathing and abdominal breathing. During coaching, 3D motion capture of their chest and abdomen was acquired with a surface scanner. Vendor software was used to measure the range of chest and abdomen motion for each of the breathing techniques.

**Results:** Quantifying the chest wall displacement using these breathing techniques demonstrated that breath hold resulted in the least amount of observed chest wall motion (0 to 5 mm) while deep breathing resulted in motion ranging up to 35 mm. Coached abdominal breathing techniques resulted in relatively less motion in the chest than the abdomen but introducing coaching increased motion when compared to the initial free breathing baseline.

**Discussion/Conclusion:** The novel 3D surface scanning method was used to capture and evaluate respiratory motion during a range of simple breathing techniques. This preliminary study found further investigation of motion management techniques is warranted for modulated radiation therapy for right‐sided breast cancer patients given the resource and patient experience implications of breath hold.

## Providing culturally responsive cancer care for First Nations peoples in Australia

### Nicholas Davis^1^

#### ^1^University of South Australia, Adelaide, Australia

**Introduction:** Advancements in evidence‐based cancer care has resulted in Australia maintaining some of the highest cancer survival rates in the world. Aboriginal and Torres Strait Islander peoples however, experience significant disparities in cancer outcomes compared with non‐indigenous counterparts.^1^ First Nations peoples recognise health to be a complex balance of physical, social, spiritual and environmental wellbeing.^2^ This intrinsic link between health, connection to land, identity, culture and community is not well reflected within the Australian healthcare system.^1^

**Learning objectives:**

- Outline barriers preventing equitable access of health care for First Nations peoples in Australia
- Identify practices that ensure the provision of culturally responsive care for First Nations peoples in Australia
- Highlight educational training modules available to medical radiation professionals to develop cultural competency.

**Discussion:** Factors preventing Aboriginal and Torres Strait Islander peoples from receiving equitable access to cancer treatment services include low health literacy, remote living locations, distrust of health services and language barriers.^3^ Health providers should endeavour to better understand the needs and concerns of Aboriginal and Torres Strait Islander peoples, communicate in a culturally respectful manner, and provide a more holistic approach to treatment to improve outcomes of health care.^2^

**Conclusion:** Aboriginal and Torres Strait Islander peoples experience poorer access to, and outcomes of care as their concept of health transcends beyond physical wellbeing. Cultural incompetence in health care correlates with compromise in quality of health outcomes.^2^ The education of medical radiation professionals to deliver culturally responsive care is imperative to providing equitable care in Australia.

**References**

1. Anderson K, Diaz A, Parikh DR, Garvey G. Accessibility of cancer treatment services for indigenous Australians in the Northern Territory: perspectives of patients and care providers. BMC Health Serv Res 2021;21(1):95.
2. Lyford M, Haigh MM, Baxi S, et al. An exploration of underrepresentation of Aboriginal cancer patients attending a regional radiotherapy service in Western Australia. Int J Environ Res Public Health 2018;15(2):337.
3. Carruthers S, Pennefather M, Ward L, Giam K, Penniment M. Measuring (and narrowing) the gap: the experience with attendance of indigenous cancer patients for radiation therapy in the Northern Territory. J Med Imaging Radiat Oncol 2019;63(4):510–16.

## Is radiomics a promising direction for the future of radiation oncology?

### Ellen McEvey,^1^ Riya Gupta,^1^ Tatiana Griffiths^1^

#### ^1^RMIT University, Bundoora, Australia

Given the rapid rate of change in radiation oncology, it is important for practitioners, specifically radiation therapists, to understand future directions of practice to ensure that they can adopt the latest developments in their own routine clinical practice.

This presentation aims to holistically evaluate radiomics, a future direction of radiation oncology practice, identifying the implications, benefits and limitations to both patients, the radiation oncology workforce and the community at large, that the implementation of this technology would have on the industry.

Medical images serve an integral role in the clinical management of radiation oncology patients. Despite this crucial role, the analysis of an image is based on a visual interpretation that is subject to intra‐ and inter‐physician variability. An advanced medical image processing paradigm, radiomics is an evolving medical field attempting to overcome this limitation to current practice.

Through extracting quantitative image features, radiomics facilitates the identification of an infinite supply of imaging biomarkers that can be used for cancer detection, diagnosis, prognosis and predicting response to treatment, ultimately improving the accuracy with which radiotherapy treatment is prescribed and delivered.

The implications of this technology on practice are significant, with the potential to prescribe personalised treatment to identified areas of high‐risk disease, predict the onset of toxicities, in addition to treatment outcomes, radiomics offers a clear benefit to patients.

However, there are also challenges to this technology, notably the limited prospective, multi‐centric research within this field, and the additional technical considerations associated with implementing a novel technique into an established clinical workflow.

## An evaluation of treatment time and intrafraction motion in stereotactic body radiation therapy

### Leila Rough,^1^ Julie Burbery,^1^ Peta Rutledge,^1^ Catriona Hargrave,^1,2^ Elizabeth Brown^1,2^

#### ^1^Queensland University of Technology, Brisbane, Australia ^2^Princess Alexandra Hospital, Brisbane, Australia

**Objectives:** This study aimed to establish treatment times for patients receiving stereotactic body radiation therapy for liver, lung or spine cancer. The magnitude of intrafraction motion exhibited by these sites as time elapses during the treatment session was also determined.

**Methods:** Retrospective image‐guided radiation therapy data for 20 patients was collected. This included imaging times and shifts made from each pre‐, during and post‐treatment cone beam computed tomography (CBCT) scan. Total treatment time, time between online image matching completion and 3D vector displacements were calculated. Descriptive statistical analysis was performed.

**Results:** The image‐guided radiation therapy data associated with 332 CBCT images was evaluated. All liver patients and 6/7 lung patients utilised breath hold techniques, while all spine patients were free breathing. The average treatment time was highest in the liver (19.3 minutes), followed by lung (14.9 minutes) and spine (14.2 minutes). Liver patients had the largest mean 3D vector displacement (0.1 cm), with 7.8% of shifts more than 3 mm. Lung patients had a mean 3D vector of 0.1 cm with 3.8% more than 3 mm, and spine patients had a mean vector of 0.02 cm with 0% more than 3 mm. Clinical tolerances were exceeded at multiple imaging timepoints (range = 4.9 to 24.4 minutes).

**Conclusion:** The results demonstrated that stereotactic body radiation therapy liver patients exhibited the greatest treatment times and 3D vector displacements, followed by lung and spine. Intrafraction imaging is required in liver and lung stereotactic body radiation therapy treatments to identify instances where clinical tolerances are exceeded.

## An evaluation of the utilisation of a six degree of freedom couch in radiation therapy

### Jessica Smith,^1^ Amy Brown,^2^ Natalie Pollard,^1^ Ashley Shackelford^2^

#### ^1^Queensland University of Technology, Brisbane, Australia ^2^Townsville Cancer Centre, Douglas, Australia

**Objectives:** This study evaluated the use of a six degree of freedom (6DoF) couch at a single radiation therapy centre to determine which patient sites required shift corrections and to analyse the patient demographic trends.

**Methods:** Patients who had received treatment with cone beam computed tomography within a period of four years were retrospectively analysed, categorised by treatment site. The sample was stratified to include patients with rotational shift applied. Descriptive and comparative statistics were used to analyse rotational and translational trends.

**Results:** 1379 patients were included. The mean absolute values for total rotations for the sites of head and neck, pelvis, abdomen, chest and other were 0.6°, 0.2°, 0.9°, 0.2° and 0.9°, respectively. Positive correlation was found with all sites requiring shifts in the sagittal rotation. Statistically significant values were found for correlations between head and neck coronal and transverse rotations with the couch lateral values (P = < 0.001), pelvis with coronal rotations and couch lateral values (P = 0.05), and for the ‘other’ site coronal and transverse rotations were correlated with couch lateral values. The adjusted R2 values were 0.3, 0.1, 0.26, 0.32 and 0.3, respectively.

**Discussion/Conclusion:** The results showed that all axes of a 6DoF couch were used for patient set‐ups. The sagittal rotation confirmed that couch sag can be corrected for with the use of a 6DoF couch for all treatment sites. The head and neck category remained the most stable set‐up site throughout all applied rotations.

## Saturday 29 April, 1:30 PM–3:00 PM

## Student Award – MI

## AI‐assisted automated detection: the future tool of detecting missed osteoporotic vertebral fractures on chest radiographs

### Cleo Wee,^1^ Zhonghua Sun^1^

#### ^1^Curtin University, Perth, Australia

**Objectives:** In this pilot study, we tested a newly developed AI software for detecting osteoporotic vertebra fractures (OVFs) on chest X‐ray not indicated for spinal disorders based on a single centre experience. Our purpose was to determine the feasibility of the AI tool for automated detection of OVFs on lateral chest X‐ray to lay the foundation for robust studies.

**Methods:** Lateral chest X‐ray of 94 elderly women (mean age = 80 years, range = 55–97 years) were retrieved from a tertiary hospital database in Perth, Western Australia, over 12 months. All chest X‐ray images were processed with the AI tool, Ofeye 1.0 which allows automated recognition of suspected fractures on lateral chest radiographs. Output metrics were analysed to determine the consistency of detecting OVFs by AI in comparison with radiological reports (by radiologists). It takes about three minutes to process 100 images with the AI software, with abnormalities such as OVFs highlighted on the chest X‐ray images.

**Results:** Of the 94 cases, AI detected seven cases of OVF that radiologists did not, thus representing 10.4% of cases that should be reported with presence of OVFs. This is clinically significant as these fractures may progress or result in significant adverse outcomes.

**Discussion/Conclusion:** For women with osteoporosis referred for chest radiographs not for spinal disorders, around 10% of vertebral fractures were missed by the radiologists. AI serves as a valuable tool to detect these missed fractures with high efficiency. Further research with inclusion of more cases is warranted to validate the clinical value of this newly developed algorithm.

## An investigation into using eye‐gaze metrics to assist chest radiograph interpretation performance: a pilot study

### Elizabeth Arthur^1^

#### ^1^Curtin University, Perth, Australia

**Aim:** To investigate the performance of health professionals in interpreting chest radiographs using eye‐tracking technology.

**Methods:** Health professionals were invited to participate in the study by interpreting five chest radiographs comprising common lung pathologies. Thirty‐two participants varied in background, including medical and radiography students (n = 7, respectively), radiographers (n = 6), radiologists (n = 5) and other specialist physicians (n = 7). Total data set collection totalled 160 interpretations with a variation in chest pathologies.

Outcome measures: The primary aim of this study investigated the relationships between expertise level and outcome factors. The secondary aim investigated the relationships between diagnostic accuracy and outcome factors. Accuracy and confidence measurements were collected via audio recording, with speed and eye‐metric data collected through the Tobii Pro Spectrum system.

**Results:** Results showed that expert groups were more diagnostically accurate than student populations (P = 0.015) irrespective of the radiograph presentation (P = 0.919). There was, however, no significant correlation between participant experience and fixation duration (P = 0.987), with image presentation significantly influencing the result (P = 0.008).

Additional analysis found significant relationships between accuracy and image presentation (P = 0.002), accuracy and confidence (P = 0.001), and accuracy and fixation duration metrics (P = 0.006). Non‐significant findings were established for relationships between diagnostic accuracy with time to first fixation and interpretation speed, respectively.

**Conclusion:** This study is one of the first studies of its kind to utilise eye tracking to explore radiological impacts with objective, quantitative data and though analysis of eye gaze during chest radiograph interpretation. This study provides additional context to the discussions surrounding expertise in radiographic interpretation.

## The cost of perfection: an investigation into unnecessary rejection of clinically acceptable imaging (lateral wrist)

### Kathryn Currie,^1,2^ Sarah Semsem,^1,3^ Adam Steward^1^

#### ^1^Western Health, Footscray, Australia ^2^Deakin University, Waurn Ponds, Australia ^3^RMIT University, Bundoora, Australia

**Introduction:** The transition from film screen to digital radiography should have brought with it a reduction in repeat rates due to a reduction in exposure‐related error. Literature suggests that this is not necessarily the case with increased positioning‐related repeats. This study aimed to assess the unnecessary reject rate of digital imaging for the five most commonly repeated projections described in literature: the lateral wrist, the lateral horizontal beam knee, shoot across horizontal beam hip, lateral lumbar spine, and antero‐posterior pelvis. This presentation describes the outcome of the first study completed in the project, the lateral wrist projection.

**Methods:** The investigation involves a reject analysis of all lateral wrist projections performed over a three‐month period at a single site. All de‐identified rejected images plus 50% of non‐rejected images were blind reviewed by a radiologist, used as the gold standard, two expert radiographers who were trained on plain film, and six junior radiographers with less than two years of digital radiography experience. The rejected images were assessed for the clinical appropriateness of the repeat to ascertain the rate of unnecessarily rejected images.

**Results:** The reject rate for adult lateral wrists over the three‐month period was 38.66%. The blinded survey results indicated junior radiographers would repeat projections almost twice as often as senior radiographers and the gold standard of the radiologist.

**Conclusion:** Our results found reject rates alarmingly higher than averages accepted in literature and a concerning trend among junior radiographers to over repeat imaging.

## Optimising horizontal beam lateral hip radiographs: analysis of radiation dose, image quality and efficiency

### Ashlyn McBurnie,^1^ Vicki Braithwaite^1^

#### ^1^Redcliffe Hospital, Brisbane, Australia

**Objectives**: There is a disparity existing within medical imaging departments regarding the performance of the horizontal beam lateral hip radiograph, a prevalent X‐ray contributing to a large proportion of departmental workflow.^1,2^ This study aims to assess current methods of obtaining the horizontal beam lateral hip projection in an experimental setting; to find the optimal technique that considers radiation dose, image quality and efficiency holistically.

**Methods**: An experimental study took place within the Queensland University of Technology Medical Imaging Simulation Laboratory using Shimadzu RADspeed pro digital radiography equipment and a full body RS‐501 anthropomorphic phantom. Eight methods of projection were tested with varying parameters of erect bucky and free detector, with grid and airgap and the use of an aluminium wedge filter. Radiation dose assessment involved dose area product in μGy.m² and absorbed dose in mGy. Timing of each method in seconds assessed efficiency. Image quality was quantified by range and standard deviation of grey values to assess image contrast and noise over a specified region of interest.

**Results**: Airgap techniques produced the least radiation dose with the use of a filter also significantly reducing radiation dose. Erect bucky techniques were most efficient. There was no clinical difference between techniques in terms of image quality, thus image quality was maintained. Overall, optimal parameters align with the erect bucky airgap with filter technique.

**Conclusion**: The recommended optimal method is the erect bucky airgap technique with filter. This finding can provide direction to help create a standardised approach and implement evidence‐based practice, improving workflow and patient outcomes. Assessment of patient data with a variety of radiographers and correlation with a subjective image analysis tool are future recommendations.

**References**

1. Australian Institute of Health and Welfare. 2018. Hip fracture incidence and hospitalisations in Australia 2015‐16. AIHW Cat. no. PHE 226. Available at https://www.aihw.gov.au/reports/injury/hip‐fracture‐incidence‐in‐australia‐2015‐16/summary.
2. Australian and New Zealand Hip Fracture Registry. ANZFHR annual report of hip fracture care. 2021. Available at https://anzhfr.org/wp422content/uploads/sites/1164/2021/12/ANZHFR_eReport2021‐FA.pdf.

## Radiological bone age assessments: should they determine whether individuals are prosecuted as children or adults?

### Josephine Awwad^1^

#### ^1^University of South Australia, Adelaide, Australia

**Background:** A posterior–anterior X‐ray of the non‐dominant hand and wrist are used in radiographic bone age assessments. The plain‐film image traditionally can be assessed under one of two standardised tests: the Greulich and Pyle method or Tanner‐Whitehouse method. Between 2008 and 2012, the Australian Government performed radiographic bone age assessments to determine whether Indonesian immigrants were children as they said they were, or people smugglers. The images were assessed under the GP method, and if proven to be adults, individuals were sent to jail for five years.

**Aim:** To investigate the limitations of radiological bone age assessments in non‐clinical settings and ensure that radiographers are participating in ethical practices.

**Discussion:** When assessing the age threshold of 18 years using the Greulich and Pyle method, radiologists look for the epiphyseal fusion of the radius. This has an accuracy of only 54.44%, as the complete fusion of the radius may not occur in males until 19 years of age. Normal variance can occur as various factors influence skeletal development. Diagnostic radiographers should limit the radiation dose, and obtain informed consent for all individuals, in keeping with practices from the Medical Radiation Practice Board of Australia.^1^ Using ionising radiation for immigration purposes, rather than for diagnostic or therapeutic reasons is a blatant violation of the ‘as low as reasonably achievable’ (ALARA) principle.

**Conclusion:** Subjecting children to ionising radiation is hard to justify when the biological or ethical risks do not outweigh the benefit of conducting this examination within a non‐clinical setting.

**Reference**

1. Medical Radiation Practice Board of Australia. Professional capabilities for medical radiation practitioners. 2020. Available at https://www.medicalradiationpracticeboard.gov.au/Registration-Standards/Professional-Capabilities.aspx.

## Radiographers' experiences of ageing population medical imaging encounters: a person‐centred approach

### Kevin Ding,^1^ Chandra Makanjee^1^

#### ^1^University of Canberra, Bruce, Australia

**Objectives:** Of the nursing home residents visiting an emergency department, approximately 85% will need a general radiographic examination and 35% will require a CT scan.^1^ Patient‐centred care is an integral component that cannot be treated in isolation from the overall medical imaging encounter. Few studies have explored a patient‐centred approach during ageing population medical imaging encounters. The aim of this study was to explore radiographers' experiences and perspectives of ageing population medical imaging encounters within a person‐centred context.

**Methods:** A qualitative explorative approach with a purposive sampling technique was used. Data collection using open‐ended interviews with radiographers practising in Australian public and private clinical settings occurred from July 2022 to October 2022. Data interpretation and analysis will be concurrent to establish preliminary categories, followed by themes and sub‐themes.

**Results:** This is a work in progress, so preliminary analysis of the data will be discussed drawing on the relevant literature. Prominent categories related to the ageing population that have emerged so far include the following: adapting to unique circumstances, communication styles, and the technicalities involved in producing a diagnostic radiograph.

**Discussion/Conclusion:** This study could inform on the development of best practice principles for person‐centred medical imaging for the ageing population. It could provide a baseline study for similar research of this nature.

**Reference**

1. Wang HE, Shah MN, Allman RM, Kilgore M. Emergency department visits by nursing home residents in the United States. J Am Geriatr Soc 2011;59(10):1864–72.

## Saturday 29 April, 3:30 PM–5:30 PM

## Champions of Change

## Person‐centred care global perspective and international community of practice

### Emma Hyde^1^

#### ^1^University of Derby, Derbyshire, United Kingdom

**Objectives**: Globally, there is increasing emphasis on ensuring health care is personalised, coordinated and enabling, and that individuals are treated with dignity and respect. Key to this is putting the person at the centre of the episode of care (rather than the clinician/process/system) to deliver person‐centred care. However, there is currently limited research that considers the different perspectives on what constitutes ‘good’ person‐centred care between the Global North and the Global South. The aim of this research was to identify the effect of socio‐economic status, different healthcare models, rural communities and cultural beliefs on both patient and professionals’ perspectives of what 'good' person‐centred care looks like in medical imaging.

**Methods**: A two‐stage mixed methods study to define informed measures of patient‐centred care in diagnostic radiography, previously carried out in the United Kingdom, was replicated in South Africa, New Zealand and Australia. Data collection was carried out using an online survey and focus groups/semi‐structured interviews. Quantitative data was analysed statistically and quantitative data was analysed using thematic analysis.

**Results**: Data collection is currently ongoing. Early results from the project will be shared at the ASMIRT 2023 Conference. Similarities and differences between research participants’ perspectives from each country will be compared.

**Conclusion**: This is the first study to compare perspectives on ‘good’ person‐centred care in medical imaging between the Global North and Global South. We hope to be able to share recommendations for ‘good’ person‐centred care which are applicable in all settings, as well as highlighting considerations specific to each country.

## Person‐centred radiation therapy models of care and services

### Michael Velec^1^

#### ^1^Princess Margaret Cancer Centre, Toronto, Canada

The radiation therapy pathway is at risk of delivering care that is impersonal, fragmented and non‐responsive to individual patient needs. Examples of efforts to re‐design radiation therapist (RT) practice using a person‐centred care approach are highlighted. For standard practice RTs, a new model of care is being developed and evaluated to re‐organise RT staff around patients in need of more personal care. Here they are partnered with one RT who provides support in addition to all technical procedures (imaging, planning, delivery) to maximise continuity of care, resulting in better preparation for treatment and overall satisfaction. More recently, in an effort to provide earlier access to RTs and tailored education, the use of virtual care was piloted. Patients opted to remotely meet their treating RT (one‐on‐one using video‐conferencing) in advance of on‐site appointments. Patients overwhelmingly endorsed this modality over both tele‐ and in‐person consultations. Using advanced practice models, RTs have even been embedded in medical consultations in an effort to offer same day breast irradiation and one‐step sim‐plan‐treat for palliation. These expansions rapid access clinics have been coupled with technology advances in (e.g. automated planning) to balance resources and maximise efficiency. Finally, the recent clinical implementation of online adaptive radiation therapy has required the daily presence of a multi‐disciplinary team for technical delivery. This has offered new patient access to their care team for support.

In summary, re‐designing services and models of care around patients, supported through a range of technologies from low‐tech to advanced, can enable RT practice to be more person‐centred.

## Dementia patient care in the medical imaging department

### Rachel Challen^1^

#### ^1^St Vincent's Hospital, Sydney, Australia

**Introduction**: People with dementia have difficulties with memory, executive functions and behaviour, which pose a challenge during diagnostic imaging. There is abundant literature on the radiographic diagnosis of dementia; however, there is little research on how to best to care for people with dementia during imaging procedures. The aim of this study is to explore the experiences of dementia care in imaging departments through the perspectives of people with dementia, carers, radiographers and student radiographers.

**Methods**: This was a cross‐sectional qualitative study. Four people with dementia and six carers participated in individual semi‐structured interviews; eight academic radiographers and 19 student radiographers participated in focus groups. Interviews and focus groups were transcribed and thematically analysed.

**Results**: Participants described positive and negative experiences during imaging procedures. Common themes existed among people with dementia, carers and radiographers. Findings were:

1. People with dementia and carers had negative experiences such as distress and pain; radiographers experienced stigma and violence
2. Negative experiences during imaging were associated with disrespected personhood, poor communication, insufficient knowledge of dementia, inappropriate time management, overly stimulating physical environments and exclusion of carers
3. Departmental protocols that contributed to negative experiences included lack of preparation, lack of dementia protocols and the use of restraints.

**Conclusions**: People with dementia and their carers can experience poor care in imaging departments and radiographers can find it difficult working with people with dementia. Radiographers need training about dementia, imaging services can improve their procedures and environment, and work in greater partnership with carers.

## Global Centre for Research and Training in Radiation Oncology – from collaboration to advocacy

### Yolanda Surjan,^1^ Laura Feighan,^1^ Debra Lee,^1^ Leah Cramp,^1^ Christine Hudson,^1^ Lauren Andreou,^1^ Jessica Cantwell^1^

#### ^1^The University of Newcastle – Global Centre for Research and Training in Radiation Oncology, Newcastle, Australia

The Global Centre for Research and Training in Radiation Oncology (GC‐RTRO), supported by the University of Newcastle, Australia, commenced operations in January of 2022 and rang the bell for the first time to mark the opening on 2 November. The Centre is the true definition of collaboration with support from leading global and national radiation oncology cancer care companies, philanthropic contributors and community advocacy groups. The GC‐RTRO is the first of its kind to bring radiation oncology research and training activities together to support medical technology industry and clinician needs; and train the next generation of radiation therapists. The Centre is focussed on elevating the standard of cancer care globally through research, training and collaboration, and developing curriculum for radiation therapy.

This presentation is an opportunity to discuss the very complex and yet interconnected world in which we live, and that the idea that engagement and collaboration, the interdependency on one another is really what ensures progress. This Centre is a part of that interdependent ecosystem with an ultimate focus on patient care. Working with industry and community drives progress, to answer difficult clinical questions and ultimately, to respond to the commitment that we, as clinicians have made which is to serve our patients' needs. This presentation is an opportunity to discuss collaboration and advocacy for radiation oncology.

## Sunday 30 April, 9:00 AM–10:30 AM

## CT – MI/NM

## Evaluation of factors influencing radiation dose: future of abdomino‐pelvic CT protocols

### Sabrine Mouslemani,^1^ Peter Kench,^1^ Ernest Ekpo^1^

#### ^1^The University of Sydney, Camperdown, Australia

**Objectives:** The concerns regarding radiation safety from CT examinations underscore the need to identify factors associated with high doses to inform optimisation strategies. Although the relationship between various CT factors and dose is well known and there are current well established dose optimisation strategies implemented in CT practice, there is a lack of understanding of the CT factors that influence dose data gathered from abdomino‐pelvic examinations. The purpose of this study is to investigate the CT factors influencing radiation dose.

**Methods:** The study utilised a secondary dataset gathered from Australian facilities, with information about the scanning parameters, patient factors and dose metrics, volume CT dose index (CTDIvol) and dose‐length product for abdomino‐pelvic examinations. Univariate analysis was performed to identify which factors were associated with CTDIvol and dose‐length product. Multiple linear regression analysis was then performed to assess the contribution of these statistically significant factors on CTDIvol and dose‐length product.

**Results:** The univariate analysis showed that patient gender, iterative reconstruction, contrast media, phases, tube voltage, starting or reference current‐time product, pitch, detector width and number of detectors were associated with both CTDIvol and dose‐length product. The multiple linear regression model showed that iterative reconstruction was the most significant determinant of CTDIvol and dose‐length product followed by whether the scan was single or multiple phases.

**Discussion/Conclusion:** Several system, acquisition and patient factors influence radiation dose in abdomino‐pelvic CT scans. Medical imaging providers should consider reviewing their iterative reconstruction algorithms, reduce the application of multi‐phase scanning by utilising dual‐energy CT and update CT scanners according to government legislations.

## Evaluation of two modified clinical prediction models for optimising the use of CT pulmonary angiography

### Jennah Turner,^1^ Rebekah Onsley,^1^ Karen Doheny,^1^ Karen Dobeli^1^

#### ^1^Royal Brisbane and Women's Hospital & STARS, Herston, Australia

**Objectives:** The purpose of this retrospective study was to determine the overall discriminatory ability of two clinical prediction model scores for the diagnosis of pulmonary embolus on CT pulmonary angiography (CTPA). The interim analysis results are presented here.

**Methods:** Institutional ethics board approval was obtained. The imaging request forms and radiology reports for all CTPAs performed at a major city hospital over a four‐year period were reviewed. Revised Geneva (rGeneva) score and modified Wells' (mWells') score (in which clinical gestalt score was excluded) were calculated from the clinical notes for the first 250 participants who met the inclusion criteria for interim analysis. Receiver operator curve analysis was performed for the two clinical prediction models. Optimal cut‐offs for each model were established, both prioritising specificity at levels of 90% and 95%, and using the Youden index which maximises both sensitivity and specificity.

**Results:** Pulmonary embolus was diagnosed on 24 of the 250 CTPAs. The areas under the curves were 0.65 and 0.69 for the rGeneva and mWells' scores, respectively. The Youden, 90% specificity and 95% specificity scores for the two clinical prediction models are presented in Table 1.

**Discussion/Conclusion:** This interim analysis suggests the two clinical predication models tested are not useful for optimising the use of CTPA for patients presenting to our hospital with any type of pulmonary embolus. Sub‐group analysis of the full dataset for lobar, segmental and subsegmental pulmonary embolus is warranted.


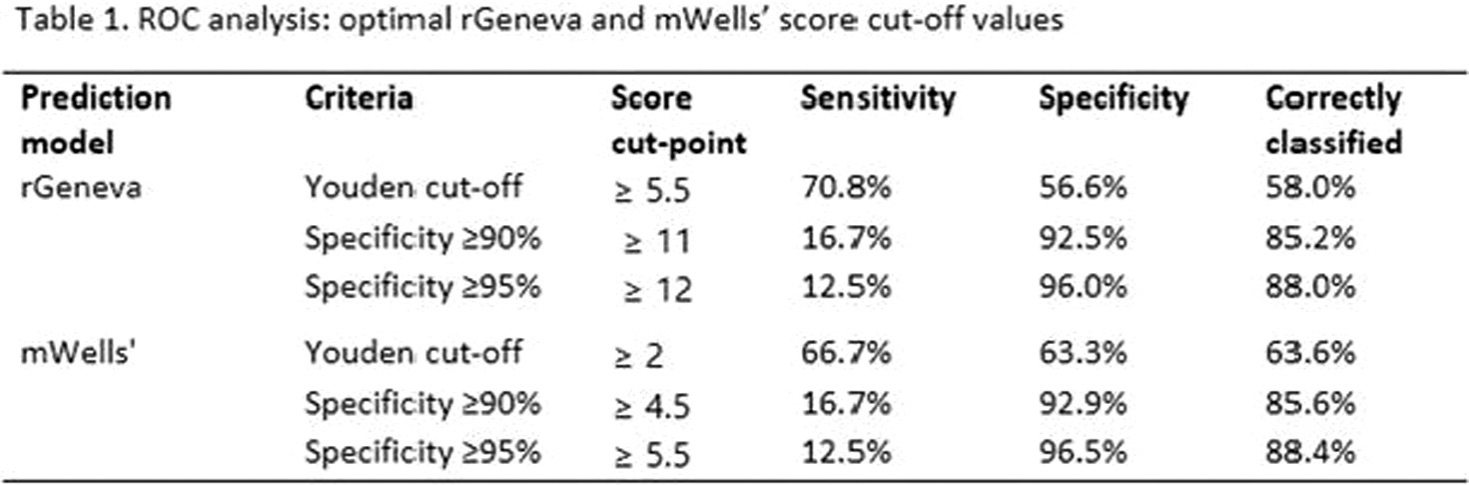


## The effect of phantom off‐centring, tube voltage and localiser direction on dose and CT number

### Yazan Al‐Hayek^1,2^

#### ^1^Charles Sturt University, Wagga Wagga, Australia ^2^The Hashemite University, Zarqa, Jordan

**Objectives:** To investigate the impact of patient off‐centring, tube voltage and localiser direction on dose and CT number accuracy. Correlations between CT number and dose change were also explored.

**Methods:** A discovery CT750 HD‐128 slice (GE Healthcare) scanner was used to scan the trunk of a PBU‐60 anthropomorphic phantom. Using the system's automatic tube current modulation, images were acquired by employing 0° and 180° localisers for different combinations of tube voltage (80, 120 and 140 kVp) and vertical off‐centring (± 30, ± 60 and ± 100 mm). The displayed volume CT dose index (CTDIvol) and CT number were recorded.

**Results:** The maximum change in dose (191% to 196%) was observed when there was closer proximity of the phantom to the X‐ray tube (± 100 mm) above and below the gantry iso‐centre. The maximum change in CT number (180° localiser and 80 kVp) as a function of vertical off‐centring was 43 HU. A strong positive correlation was reported between the variation in dose and CT number (r = 0.969, P < 0.001, 95% CI 0.93–0.99).

**Conclusion:** As the literature reports that a considerably large percentage of CT patients are off‐centred vertically, and more likely below the gantry iso‐centre, this study recommends employing the 0° localiser and applying lower tube voltage where applicable. It is also crucial for those involved in CT imaging to have an in‐depth understanding of the limitation of CT systems to avoid potential pitfalls in clinical decision‐making when using CT numbers as an absolute value for tissue lesion characterisation.


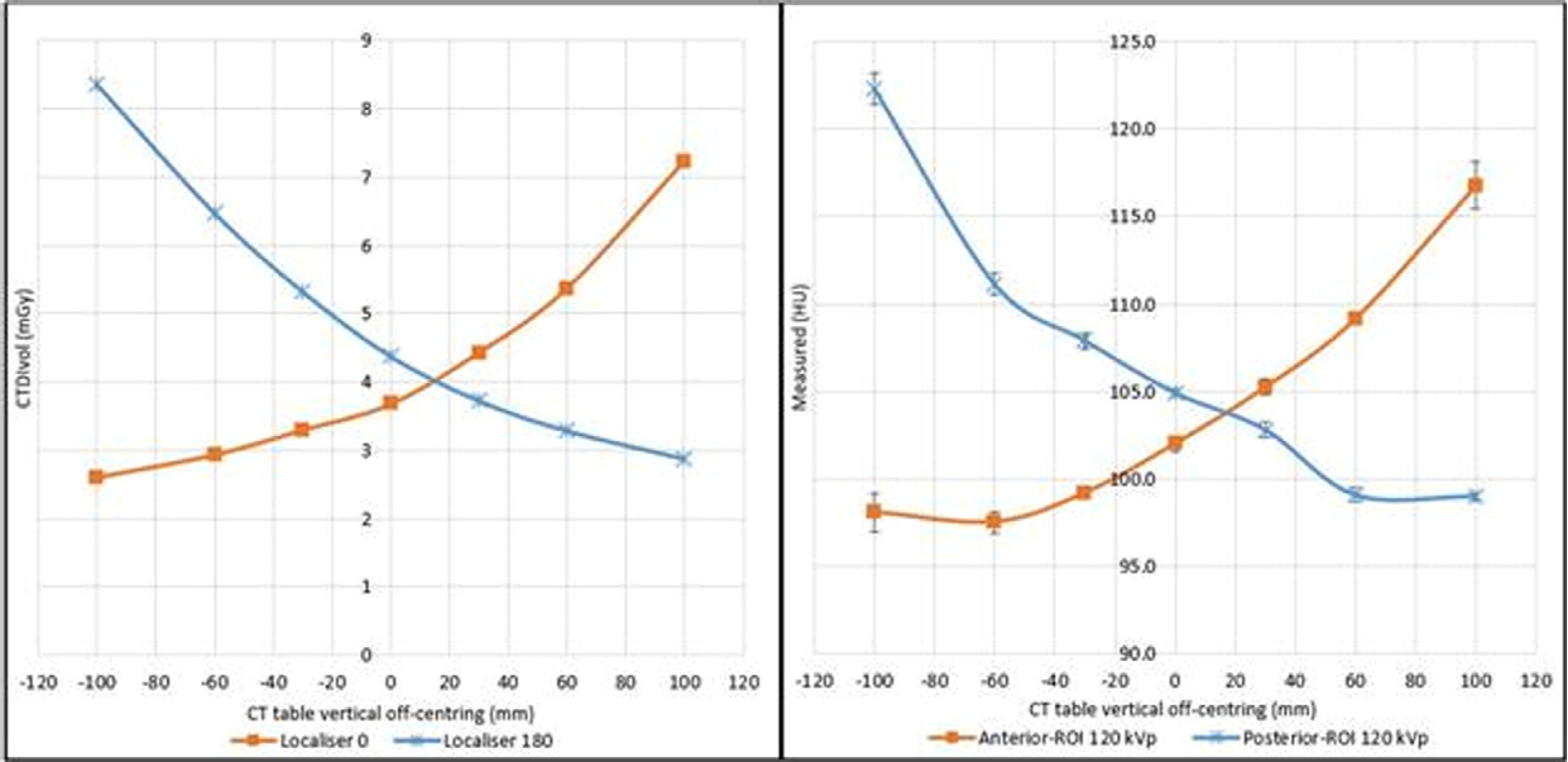


## CT venography for cerebral venous thrombosis: are we imaging wisely?

### Emma Thornton,^1^ Mykaela Gordon,^1^ Karen Doheny,^1^ Karen Dobeli^1^

#### ^1^Royal Brisbane and Women's Hospital & STARS, Herston, Australia

**Objectives:** This audit was performed to determine if optimisation of the requesting practices and/or imaging protocol for CT venography to exclude cerebral venous thrombosis (CVT) is warranted to reduce the radiation and healthcare costs of this examination.

**Methods:** Institutional ethics board approval was obtained. The referral indications, radiological diagnosis and CT protocol (number of phases and dose length protocol) for all CT venograms performed to diagnose/exclude CVT at a major city hospital between 24 August 2018 and 17 June 2022 were documented. Descriptive statistics were calculated for CVT diagnoses, alternate diagnoses, normal studies, scan phases performed and radiation dose.

**Results:** 530 relevant CT venography scans were performed during the audit period. Of these, CVT was diagnosed on five (1.0%), excluded on 523 (98.6%) and unable to be confirmed or excluded on two (0.4%). A radiologist report of ‘normal study’ was made on 376 (70.9%) studies in which CVT was excluded. 446 (84.0%) patients underwent two scan phases (non‐contrast and venogram), four (0.8%) had three phases (non‐contrast, venogram, post‐contrast). The remaining 81 (15.2%) had a single venogram phase scan. Of these, 30 (37.0%) had undergone a non‐contrast scan within the prior 24 hours. The average dose length product was 1716 mGy.cm, which equates to an approximate effective dose of 3.6 mSv.

**Discussion/Conclusion:** The prevalence of CVT on CT venogram at the study site is extremely low, and most examinations are normal. In the interests of reducing unnecessary CVTs, investigation into the use of virtual non‐contrast reconstructions +/− a clinical assessment tool is warranted.

## Diagnostic CBCT: creating novel 3D imaging capacity or a technology looking for a home?

### Beverly Snaith^1,2^

#### ^1^University of Bradford, Bradford, United Kingdom ^2^Mid Yorkshire Hospitals NHS Trust, Wakefield, United Kingdom

Cone beam computed tomography (CBCT) has an accepted role in image‐guided radiotherapy. However, in diagnostic imaging the hybrid technology is yet to become a frontline imaging resource beyond the dental sector. Based on digital technology, new applications in musculoskeletal practice as a result of different manufacturer's approaches are enabling 3D imaging with lower dose and cost. These now include weightbearing peripheral and spinopelvic imaging. As a relatively low dose application with some of the positioning challenges of general radiography there remains a question as to how, and where, this becomes integrated into mainstream practice.

This presentation will consider the emerging application of cone beam CBCT in diagnostic imaging and review the role medical radiation practitioners must take to develop the evidence base to effectively implement such technology.

## Sunday 30 April, 9:00 AM–10:30 AM

## Educating the Future – Combined

## Theoretical frameworks for radiography podcast development

### Yobelli Jimenez,^1^ Amanda Punch,^1^ John Atyeo,^2^ Tooba Zaidi,^3^ Nareuchaya Karoonuthaisiri,^1^ Emily Girard^1^

#### ^1^The University of Sydney, Sydney, Australia ^2^Northern Sydney Local Health District, Sydney, Australia ^3^Government of Western Australia, Australia

**Objectives:** The scope of podcasts in health and medicine has grown rapidly over the past few years. Podcast content and delivery style can be versatile, and hence podcasts offer a unique learning environment for university students. The aim of this presentation is to describe the use of cognitive theory of multi‐media learning and adult learning theory as frameworks for podcast development.^1,2^

**Methods:** Following a pilot study, insights into radiography students' perceptions and learning needs that could be supported by podcast content were identified. The cognitive theory of multi‐media learning and adult learning theory were used to provide structure to 10 newly developed podcasts. Each podcast focussed on one topic within the field of radiography.

**Results:** Five academic‐led and five student‐led podcasts were developed on a range of topics including non‐accidental injury, patients who self‐harm, and students' personal experiences in the radiography course. Three main considerations applied from cognitive theory of multi‐media learning were to reduce extraneous stimuli, segmenting podcasts into key points, and using social cues to motivate the listener to understand the content. From the adult learning theory, key assumptions were made about intended podcast audience, namely that radiography students have self‐concept, personal and professional experiences, readiness to learn, orientation to learning and motivation to learn.

**Discussion/Conclusion:** The cognitive theory of multi‐media learning and adult learning theory were practical frameworks for podcast development, in that they provided a structure for presentation of content within the podcasts, as well as a theoretical foundation underlining podcasts for future evaluation.

**References**

1. Mayer RE. Applying the science of learning: evidence‐based principles for the design of multimedia instruction. Am Psychol 2008;63(8):760–9.
2. Knowles MS. Self‐directed learning: a guide for learners and teachers. New York: Association Press; 1975.

## Getting to know you: interprofessional learning workshops with radiation therapy students and radiation oncology registrars

### Lisa Cunningham^1^

#### ^1^University of South Australia, Adelaide, Australia

**Background:** Radiation therapy is a multi‐disciplinary field, and graduates must be able to collaborate with other healthcare practitioners.^1^ Skills in teamwork and interprofessional communication are key to these collaborations and must be incorporated into radiation therapy training.^2,3^ Interprofessional learning workshops between radiation therapy students and radiation oncology registrars can improve participants' teamwork and technical skills, as well as improving their understanding of each other's professional roles,^3^ and were piloted in the training programs for these groups in South Australia for the first time in 2022.

**Content:** An interprofessional learning workshop series for final‐year radiation therapy students and radiation oncology registrars was held. Three sessions were held over one year, with a focus on radiation therapy dosimetry. Sessions increased in complexity, with time initially for participants to get to know each other and talk through their roles. In the final session, participants worked in small groups to complete a palliative planning case from volume delineation to plan evaluation.

**Conclusion:** The pilot interprofessional learning workshop series between radiation therapy students and radiation oncology registrars was successful, based on informal evaluation feedback from both cohorts. Future series will involve formal research into the immediate benefits for the trainees, as well as how improved collaboration skills may carry on to our future professionals.

**References**

1. Medical Radiation Practice Board. Professional capabilities for medical radiation practitioners. 2020. Available at https://www.medicalradiationpracticeboard.gov.au/Registration-Standards/Professional-Capabilities.aspx.
2. Dubois N, Diep AN, Ghuysen A, et al. Training of radiotherapy professionals: status, content, satisfaction and improvement suggestions in the greater region. BMC Med Educ 2022;22(485).
3. Ball B, Kirby M, Ketterer SJ, et al. Radiotherapy‐specific interprofessional learning through simulation. Radiography 2020;27(1):187–92.

## MRI in radiation therapy: educational opportunities shaping the future for current and future practitioners

### Julie Burbery,^1,2^ Crispen Chamunyonga^1,2^

#### ^1^Queensland University of Technology, Brisbane, Australia ^2^Centre for Biomedical Technologies, Brisbane, Australia

The utilisation of magnetic resonance (MR) technology in radiation therapy through dedicated MR simulation and MR‐linac technology is increasing. The greatest challenge is the training and education of radiation therapists to ensure the safe and effective operation of MR technology.^1–3^

In 2019, professionals including radiation therapists, radiographers, medical physicists and a radiologist were involved in the development of a comprehensive continuing professional education program that commenced in June 2020. The successful implementation of the continuing professional education program provides an avenue for upskilling radiation therapy practitioners nationally and internationally. Under phase 1, eight modules were developed to address MR physics, safety, quality assurance, imaging protocols, image interpretation, multi‐modality image registration and the use of contrast in MR imaging with an opportunity to undertake a practical module. Developments in the undergraduate radiation therapy program include the incorporation of both didactic elements with practical consolidation. This is aimed at ensuring that graduates have ample opportunities to meet the needs of a changing clinical environment, practice standards and professional requirements. The program is providing opportunities for radiation therapy students to develop knowledge which can be applied in many areas such as multi‐modality image registration for planning, adaptive therapy and image‐guided radiation therapy.

The provision of educational opportunities for the current and future radiation therapy workforce in the use of MR technology is paramount. However, ongoing development is necessary, requiring collaborative initiatives and support from industry, clinical departments, vendors, MR experts and professional bodies.

**References**

1. Eccles CL, Campbell M. Keeping up with the hybrid magnetic resonance linear accelerators: how do radiation therapists stay current in the era of hybrid technologies? J Med Imaging Radiat Sci 2019;50(2):195–8.
2. Chin S, Eccles CL, McWilliam A, et al. Magnetic resonance‐guided radiation therapy: a review. J Med Imaging Radiat Oncol 2020;64:163–77.
3. Glide‐Hurst CK, Paulson ES, McGee K, et al. Task group 284 report: magnetic resonance imaging simulation in radiotherapy: considerations for clinical implementation, optimization, and quality assurance. Med Phys 2021;48:e636–e70.

## Becoming champions of change: organising and delivering CPD in a COVID and post‐COVID world

### Therese Gunn,^2,3^ Alex Hollingsworth^1,2,3^

#### ^1^Princess Alexandra Hospital, Brisbane, Australia ^2^Queensland University of Technology, Brisbane, Australia ^3^Queensland Continuing Education Committee (ASMIRT), Brisbane, Australia

Delivering continuing professional development events has been the role of our committee for several decades. Our usual event schedule has been three seminars throughout each year, held at centrally located venue in Brisbane city. Like all face‐to‐face events, the COVID‐19 pandemic presented significant challenges, and as a committee we needed to band together with the support of our head office to become ‘champions of change’.

After a tumultuous 2020, 2021 saw us return to some semblance of our previous annual schedule, with our committee offering a virtual seminar experience. This saw us reach a larger audience, not just within eastern Queensland, but also inclusive of rural Queensland, other states and territories and even a small overseas attendance.

Behind the scenes however, there was significant anxiety and challenges to overcome as well as the fear of the unknown. Many issues we experience as a committee did not present themselves until the day of the seminar, and the main challenge for us was for the show to go on. Many abilities and skill sets were utilised, and this only served to highlight that you do not know what skill sets you have until you need them (often under pressure!).

With 2023 seeing a more reliable return to face‐to‐face seminars, our presentation will take you through our hurdles, reflections, and the importance of strong communication between stakeholders to improve infrastructure and capacity for the future.

## Translating research in medical radiation practice: shaping the future of patient care

### Christopher Hicks^1^

#### ^1^West Moreton Health, Ipswich, Australia

**Introduction:** This paper explores the concept of research translation and its application in the medical radiation practice professions. A concept of research translation that extends beyond the familiar ‘bench to bedside’ or translation of pure scientific research into clinical practice, to a consideration of processes that ensure clinicians practise in an evidence‐informed manner and that health systems are organised and co‐ordinated for improved access and outcomes for patients.^1^

**Methods:** A range of research translation frameworks are explored, including Glasziou and Haynes' work, which focusses on the individual practitioner's attempt to bridge the divide between the volumes of research literature produced and the clinician's uptake of evidence into practice; Matus et al's systematic review of research pertaining to allied health professions research capability building; and Harvey and Kitson's iPARIHS framework, which takes account of the clinical practice environment where research is adopted into practice.^2–4^

**Discussion:** The merits of the various research translation frameworks applied to a medical radiation practice context is explored. The extension of the professional responsibility to undertake research to include research translation provides opportunities for medical radiation practitioners to practise in an evidence‐informed way, implement improvements in practice and thereby shape a future characterised by improved patient experience and outcomes.

**References**

1. Woolf S. The meaning of translation research and why it matters. JAMA 2008;299(2):211–3.
2. Glasziou P, Haynes B. The paths from research to improved health outcomes. Evid Based Nurs 2005;8(2):36–8.
3. Matus J, et al. Research capacity building frameworks for allied health professionals – a systematic review. BMC Health Services Research 2018;18:716.
4. Harvey G, Kitson A. PARIHS revisited: from heuristic to integrated framework for the successful implementation of knowledge into practice. Implement Sci 2016;11(33):1–13.

## Student radiographers' observations of evidence‐based practice during placements: implications for radiography education, culture and practice

### Laura Di Michele,^1^ Amani Bell,^1^ Kate Thomson,^1^ Mark McEntee,^2^ Belinda Kenny,^3^ Warren Reed^1^

#### ^1^The University of Sydney, Sydney, Australia ^2^University College Cork, Cork, Ireland ^3^University of Western Sydney, Sydney, Australia

Evidence‐based practice and, more specifically, evidence‐based radiography, is widely acknowledged as fundamental to good practice.^1–4^ Students in Australian universities undertake a range of clinical placements to qualify for national registration as radiographers, this places them in the unique position of observing varied practice at a wide range of clinical centres. The purpose of this study was to explore students' experiences of evidence‐based practice during clinical placements, the impact of these experiences on their education, and their perspective of evidence‐based practice tendencies in the profession.

Students who had completed a minimum of three clinical placements at different sites were invited to participate in focus group discussions that explored their experiences of evidence‐based practice in clinical settings. The data were then analysed utilising reflexive thematic analysis.

Three themes were identified: education; culture and responsibility; and hopes, fears and barriers. The theme, culture and responsibility was further broken down into three sub‐themes: professional, organisational and individual. A wide variety of students' experiences of evidence‐based practice on placement contributed to varied perceptions of the concept. Although participants had a clear and strong desire to implement evidence‐based practice while on clinical placement, they felt limited by poor educational preparation and disempowered by workplace cultures and barriers. These findings have implications for radiography educators, managers and leaders, and those interested in advancing the profession.

**References**

1. Smith T. Evidence based medical imaging (EBMI). Radiol Technol 2009;80(3):270–5.
2. Hafslund B, Clare J, Graverholt B, Wammen Nortvedt M. Evidence‐based radiography. Radiography 2008;14(4):343–8.
3. Gambling T, Brown P, Hogg P. Research in our practice—a requirement not an option: discussion paper. Radiography 2003;9(1):71–6.
4. Sackett DL, Rosenberg WMC, Grey JAM, Haynes RB, Richardson WS. Evidence based medicine: what it is and what it is not. BMJ 1996;312(7023):71–2.

## Sunday 30 April, 9:00 AM–10:30 AM

## Closing the Gaps – RT

## Translating the treatment: educating and instructing non‐English speaking patients for DIBH/EEBH radiation therapy

### Andrew Puffett,^1^ Ming Zhao^1^

#### ^1^Princess Alexandra Hospital, Gold Coast, Australia

The ability to convey complex information about radiation therapy in a clear, concise and consistent manner to non‐English speaking patients often requires the use of dedicated education resources and/or interpreter services that at times can be difficult to obtain.

For patients who require interactive radiation therapy techniques such as deep inspiration breath hold (DIBH) or end expiration breath hold (EEBH) using the Elekta Active Breathing Coordination System, the capacity to communicate in a common language is a factor that may influence whether these treatments can be successfully applied. To ensure equity in the provision of these radiation therapy techniques for non‐English speaking patients, the Princess Alexandra Hospital Radiation Oncology Department has developed several app‐based resources that assist staff to educate, set‐up and deliver DIBH and EEBH treatments to patients in their native language.

In this presentation we will discuss the cultural considerations made in developing the Mandarin language module for our DIBH and EEBH voice command application and the thought process behind its linguistic architecture.

We will also showcase how our app‐based resources are utilised in a clinical environment and examine the advantages and disadvantages in providing education and instruction to non‐English speaking patients via this software platform.

## The experience of CALD patients in radiation therapy – how can we improve patient‐centred care?

### Freshta Mohammad^1^

#### ^1^University of South Australia, Adelaide, Australia

**Introduction:** This presentation will consider the complexities of working with patients of culturally and linguistically diverse (CALD) backgrounds and highlight factors that impact the care they receive through a unique patient case. Studies show CALD patients tend to have increased complications and difficulties with the management of side‐effects ensuing a greater level of dissatisfaction with healthcare services.^1^ The challenges for clinicians when interacting with CALD patients will also be addressed.

**Case Presentation:** The patient was a 57‐year‐old female refugee, originally diagnosed with early‐stage breast cancer in 2019 and received treatment for it overseas. Due to her refugee status and lack of health insurance she received no further follow up and then presented in Australia with a more advanced recurrence.

**Management/Outcome:** Given her advanced staging, her initial treatment was hormone therapy followed by a course of palliative radiation therapy, which she unfortunately did not complete. Due to the language barrier present, the patient found it difficult to adhere to the recommended skincare regimen, resulting in her experiencing a severe skin reaction and difficulty maintaining the treatment position.

**Discussion:** Clinicians adopt a range of strategies to reduce communication barriers, however there are concerns about the quality and extent of information provided. Language barriers also affect the level of support and the treatment techniques available to CALD patients, creating a disparity of care.^2^ This case illustrates that further investigation into supporting CALD patients receiving radiation therapy is necessary for the best patient‐centred care to be delivered.

**References**

1. Qureshi MM, Romesser PB, Jalisi S, et al. The influence of limited English proficiency on outcome in patients treated with radiotherapy for head and neck cancer. Patient Educ Couns 2014;97(2):276–82.
2. Shukla U, Sueyoshi M, Diamond B, et al. Disparities in radiation therapy: practice patterns analysis of deep inspiratory breath hold use in non‐English speakers. Int J Radiat Oncol Biol Phys 2022;113(1):21–5.

## Increasing recruitment of CALD populations to MRI clinical trials in radiation oncology

### Doaa Elwadia,^1,2^ Kylie Dundas,^1,3,4^ Ben ‘Allan’ Smith,^3,4^ Penny Phan,^1,3^ Vivian Nguyen,^1,3^ Trang Pham,^1,3,4^ Robba Rai^1,3,4^

#### ^1^Liverpool and Macarthur Cancer Therapy Centres, Liverpool, Australia ^2^The University of Sydney, Sydney, Australia ^3^Ingham Institute for Applied Medical Research, Liverpool, Australia ^4^The University of New South Wales, Sydney, Australia

**Introduction:** In southwest Sydney, 45% of the population speak a language other than English.^1^ Clinical trials participation from culturally and linguistically diverse (CALD) populations is low, with communication and health literacy concerns identified as factors affecting participation.^2^ The objective of this study is to test the effect of an educational intervention that aims to increase CALD participation rates in already recruiting MRI‐based clinical trials.

**Method:** A multi‐modal educational intervention was developed that highlighted the value of clinical trial participation and introduced MRI and radiotherapy concepts. A cross‐sectional pre‐ and post‐intervention survey was developed to assess the impact of the educational intervention. Survey questions assess participants' inclination to participate in MRI‐based clinical trials pre‐ and post‐educational intervention. The educational materials and survey were translated into the two most common languages (other than English). Anticipated sample size will be 100 participants.

**Results:** In this interim analysis, 23 participants have been recruited. Most participants (64%) indicated they had not seen any information regarding clinical trial participation in their language, and that their English proficiency impacts their willingness to participate in a clinical trial (57%), with 76% of responders indicating that having information pertaining to MRI and clinical trials in their preferred language would increase their willingness to participate in these trials. The number of responders unlikely to participate in an MRI clinical trial decreased after the educational intervention (24% vs. 5%).

**Conclusion:** Interim results indicate that the educational resource could positively impact CALD recruitment rates.

**References**

1. South West Sydney: Our Health ‐ Ministry of Health. An in‐depth study of the health of the population now and into the future. 2019. Available at https://www.swslhd.health.nsw.gov.au/pdfs/SWS%20Our%20Health%20in%20depth.pdf.
2. Smith AB, Agar M, Delaney G, et al. Lower trial participation by culturally and linguistically diverse (CALD) cancer patients is largely due to language barriers. Asia Pac J Clin Oncol 2018;14(1):52–60.

## A modern approach to treating stereotactic ablative body radiotherapy/stereotactic radiosurgery in regional communities

### Marissa Morey^1^

#### ^1^Dubbo Cancer Care Centre, Dubbo, Australia

Statistics show that people living in rural and remote Australia have reduced access to health services and treatment while also experiencing poorer health outcomes than those living in metropolitan areas.^1^ These communities experience lower life expectancy and are more likely to die younger, especially from potentially avoidable deaths, than their counterparts living in major cities.^1^

Traditionally, a radiation oncologist would be present during stereotactic ablative body radiotherapy and stereotactic radiosurgery treatments. Unfortunately, in regional Australia many facilities do not have the luxury of having an oncologist on site. To overcome this, a relationship was built with our metropolitan radiation oncologists to allow us to utilise stereotactic body radiation therapy and stereotactic radiosurgery techniques. Radiation therapists video call the oncologist during the imaging scan and use this technology to communicate thoughts around image matching.

Providing these treatment options means patients do not have to travel to a major city to receive high quality care. This is especially beneficial for patients that travel long distances for treatment, often leaving their work, country and families behind. Providing patients with high quality treatment course options enables them to return home much sooner.

We are forging a path to a new, modern way of treating and helping to reduce the gap in health outcomes for people dealing with cancer in rural and remote communities.

**Reference**

1. Australian Institution of Health and Wellness. Australian Government: rural and remote Australians. 2022. Available at https://www.aihw.gov.au/reports/rural-remote-australians/rural-and-remote-health.

## Persisting gaps in optimal care of stage III non‐small‐cell lung cancer: an Australian patterns of care analysis

### Katrina Woodford,^1,2,3^ Kendrick Koo,^1,2,4^ John Reynolds,^4^ Robert Stirling,^5,6^ Susan Harden,^1,4^ Margaret Brand,^4^ Sasha Senthi^2,3^

#### ^1^Peter MacCallum Cancer Centre, Melbourne, Australia ^2^Alfred Health Radiation Oncology, Melbourne, Australia ^3^Monash University Central Clinical School, Melbourne, Australia ^4^Monash University, Melbourne, Australia ^5^Monash University, Clayton, Australia ^6^Alfred Health, Melbourne, Australia

**Objectives:** Wide variation exists globally in the treatment and outcomes of stage III non‐small cell lung cancer (NSCLC) patients. We conducted an up‐to‐date patterns of care analysis in the state of Victoria, Australia, with a particular focus on the proportion of patients receiving treatment with radical intent, treatment trends over time and survival.

**Methods:** Stage III NSCLC patients were identified in the Victorian Lung Cancer Registry and categorised by treatment received and treatment intent. Logistic regression was used to explore factors predictive of receipt of radical treatment and the treatment trends over time. Cox regression was used to explore variables associated with overall survival. Co‐variates evaluated included age, gender, ECOG performance status, smoking status, year of diagnosis, Australian born, Aboriginal or Torres Strait Islander status, socioeconomic status, rurality, public/private status of notifying institution and multi‐disciplinary meeting discussion.

**Results:** A total of 1396 patients were diagnosed between 2012 and 2019 and received treatment with radical intent 67%, palliative intent 23%, unknown intent 5% and no treatment 5% (see Table). Radical intent treatment was less likely if patients were more than 75 years of age, ECOG ≥1, had T3–4 or N3 disease or resided rurally. Surgery use decreased over time, while concurrent chemoradiotherapy and immunotherapy use increased. Median overall survival was 38.0 months, 11.1 months and 4.4 months following radical treatment, palliative treatment or no treatment, respectively (P < 0.001).

**Conclusion:** Almost a third of stage III NSCLC patients still do not receive radical treatment. Strategies to facilitate radical treatment and better support decision‐making between increasing multi‐modality options are required.


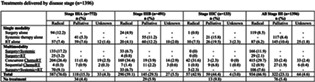


## A preliminary review of the issues associated with treating patients with a larger body habitus

### Amanda Bolderston,^1^ Megan Brydon,^1^ Jenna Maclaine^1^

#### ^1^University of Alberta, Edmonton, Canada

**Objectives**: Obesity is generally reported as having a significant impact on health care practice and systems, although there is also growing recognition that pervasive weight stigma can affect access to health care and/or cause negative health effects.^1,2^ This scoping review sought to answer the question: How has imaging and treating patients with a larger body habitus been defined, classified, and understood in the medical radiation sciences literature?

**Methods**: A scoping review of English language peer‐reviewed papers published from 2011 to 2021 was performed using the PRISMA framework. Three independent reviewers selected papers with the inclusion criteria: English language, human subjects, obese, bariatric, fat, medical imaging, medical radiation technology, radiation therapy and radiography.

**Results**: The initial search identified 8809 articles; 8463 studies were screened and 347 full text studies assessed for eligibility. 80 studies were included for final data extraction. Of the articles included, preliminary qualitative content analysis revealed three major themes. The majority of papers looked at imaging and/or dose issues as well as equipment and environment. Fewer papers discussed patient care. There were specific radiation therapy considerations related to technique, dosimetry and acute side effects. Many of the radiography and radiation therapy specific papers were reviews and commentaries rather than research studies. Analysis is ongoing.

**Conclusion**: A growing number of studies in this area have been published with both practical and patient care implications. Preliminary qualitative analysis revealed that major themes in the medical radiation sciences literature consist of equipment and technical considerations.

**References**

1. Rubino F, Puhl RM, Cummings DE, et al. Joint international consensus statement for ending stigma of obesity. Nat Med 2020;26(4):485–97.
2. The Lancet Public Health. Addressing weight stigma. Lancet Public Health 2019;4(4):e168.

## Sunday 30 April, 9:00 AM–10:30 AM

## You Matter – Combined

## Moral distress during COVID‐19: honouring our experiences and growing our resilience for the future

### Tom Steffens^1^

#### ^1^Princess Alexandra Hospital, Brisbane, Australia

Health care is described as inherently moral and ethical, aimed at improving the welfare of individuals and protecting the health of society.^1^ Australian radiographers are expected to discharge their duties with high ethical standards, in accordance with relevant legislation and regulations.^2–4^

The field of ethics is rooted in the study of morals. Essentially, professional ethics are concerned with encouraging practitioners to do the right thing. While there are myriad moral philosophies, most fall into one of two categories: deontology (concerned with the morality of each act) and utilitarianism/consequentialism, which places priority on the morality of outcomes.^5^ Australian society adopts a pluralistic moral approach, incorporating both deontological and consequentialist philosophies according to situational and individual perspectives.^6^

Moral distress occurs when individuals are prevented from acting according to their personal values due to legislation, organisational policies or prevailing conditions.^7^ In Australia's morally pluralistic society, such distress is considered inevitable, especially in health care.^6^ Before the COVID‐19 pandemic, moral distress experienced by healthcare workers was already prevalent, with reported events ranging from moral dilemmas (low amplitude, high frequency) to morally injurious (high amplitude, low frequency).^8^ The pandemic's unique challenges have caused significant increases in moral distress and fatigue, with associated deleterious impacts on healthcare workers.^9^

This presentation will:

- Review moral principles and how moral differences between individuals leads to moral distress and fatigue
- Discuss morally injurious events encountered by medical radiations practitioners during the COVID‐19 pandemic
- Describe sequelae of moral distress and outline practical mitigation tips at individual, team and organisational levels.

**References**

1. Kerridge I, Lowe M, Stewart C. Ethics and law for the health professions (5th edn). Leichardt, Australia: The Federation Press; 2013.
2. Health Practitioner Regulation National Law (Queensland). 2018. Available at https://www.legislation.qld.gov.au/view/pdf/inforce/2018-03-01/act-2009-hprnlq.
3. Medical Radiation Practice Board of Australia. Code of conduct. 2014. Available at http://www.medicalradiationpracticeboard.gov.au/Codes-Guidelines/Codes-and-Guidelines/Code-of-conduct.aspx.
4. Australian Society of Medical Imaging and Radiation Therapy. Code of ethics. 2017. Available at https://www.asmirt.org/media/124/124.pdf.
5. Garbutt G, Davies P. Should the practice of medicine be a deontological or utilitarian enterprise? J Med Ethics 2011;37(5):267–70.
6. Tigard DW. Rethinking moral distress: conceptual demands for a troubling phenomenon affecting health care professionals. Med Health Care Philos 2018;21(4):479–88.
7. Riedel PL, Kreh A, Kulcar V, Lieber A, Juen B. A scoping review of moral stressors, moral distress and moral injury in healthcare workers during COVID‐19. Int J Environ Res Public Health 2022;19(3):1666.
8. Kherbache A, Mertens E, Denier Y. Moral distress in medicine: an ethical analysis. J Health Psychol 2022;27(8):1971–90.
9. Smallwood N, Pascoe A, Karimi L, Willis K. Moral distress and perceived community views are associated with mental health symptoms in frontline health workers during the COVID‐19 pandemic. Int J Environ Res Public Health 2021;18(16):8723.

## ePortfolios: a new educational approach to stop, think and say something!

### Magdalena Dolic,^1^ Yaxuan (Lisa) Peng,^2^ Keshav Dhingra^3^

#### ^1^Western Health, Footscray, Australia ^2^Monash Health, Clayton, Australia ^3^Capital Radiology, Epping, Australia

**Objectives:** In 2020, the Medical Radiation Practice Board of Australia made several revisions to its professional capabilities.^1^ This included ‘See something, say something’ which requires medical radiation practitioners to escalate urgent findings.^1^ Despite this, radiographers' confidence articulating descriptions of radiographic findings varies.^2^ This mixed‐methods study explores how the implementation of ePortfolio affects student confidence in identifying and describing radiographic findings in both an academic and a clinical setting.

**Methods:** A Qualtrics survey comprised of both quantitative and qualitative questions was distributed to second‐year Radiography students who had used ePortfolios. The survey comprised of four quantitative questions using a Likert‐scale and one qualitative question. Quantitative data was analysed using the Wilcoxon signed ranked test and qualitative data was thematically assessed.

**Results:** 55 out of 65 participants (85%) completed the survey. Positive responses (strongly agree and agree) decreased from 89% to 74% between academic and clinical environments when identifying abnormalities, and 89% to 73% when describing findings. This highlights the challenges students face when in the clinical environment; also identified in the qualitative data where three recurring themes were identified among responses. Wilcoxon signed ranked test analysed a statistically significant relation between the two environments (P < 0.05). However, the relationship between identifying and describing skills was not statistically significant (P > 0.05).

**Discussion/Conclusion:** ePortfolios assist in improving confidence in the identification and description of abnormalities, particularly in an academic setting. The clinical environment presents unique challenges which may limit student clinical performance however, this requires further investigation.

**References**

1. Medical Radiation Practice Board of Australia. Professional capabilities for medical radiation practice. 2020. Available at http://www.medicalradiationpracticeboard.gov.au/Registration/Professional-Capabilities.aspx.
2. Neep MJ, Steffens T, Owen R, McPhail SM. A survey of radiographers' confidence and self‐perceived accuracy in frontline image interpretation and their continuing educational preferences. J Med Radiat Sci 2014;61(2);69–77.

## e‐learning created by radiographers – shaping the future of medical imaging education

### Daniel Ho,^1,2^ Karen Thomas^1,2^

#### ^1^Health Support Services, Perth, Australia ^2^Fiona Stanley Hospital, Murdoch, Australia

**Background:** Western Australia's Medical Imaging Replacement Program is replacing its existing AGFA picture archiving and communication system and radiation information system with the Enterprise Medical Imaging Platform (EMIP) solution. The EMIP is a consortium of more than 15 applications. Considering the impact of COVID‐19 on face‐to‐face training delivery, e‐learning was the primary method of staff training for the EMIP applications. A team of radiographers, who were internal staff, were responsible for the design and development of the e‐learning courses, using Articulate Rise and Articulate Storyline software.

**Discussion:** The creation and management of e‐learning by internal radiographers resulted in numerous benefits, compared to outsourcing this task to an external learning and development team. The insider expertise from radiographers ensured that e‐learning content was targeted to the audience and responsive to changes in working practices. As the EMIP applications frequently evolved, the e‐learning could be regularly and directly updated by radiographers to maintain content accuracy. These updates were efficiently deployed, by eliminating the need for repeated engagement with an external developer. Considering the time investment required in creating bespoke, continually updated courses, radiographer‐designed learning was more cost‐effective than procuring e‐learning from an external team.

**Conclusion:** Radiographer‐designed e‐learning was effective and responsive to successfully train staff in using the EMIP applications. Opportunities for radiographer‐designed e‐learning could be further explored for ongoing medical imaging staff education, including professional development and modality training.

## Burnout or lean in – is professional supervision the answer?

### Sarah Thomson^1^

#### ^1^Christchurch Oncology, Christchurch, New Zealand

In a career crisis? Would you choose contacting workplace support and taking stress leave or would you proactively invest in your career with professional supervision?

I believe professional supervision will ensure radiation therapists have sustainable fulfilling careers and are confident and reflective in their practice. This is our goal at Christchurch Oncology with a professional supervision pilot study, where we are conducting monthly sessions with nine radiation therapists over a 12‐month period.

The aims of our sessions are for the radiation therapists to walk away seeing them as a learning experience and having a richer capacity on how to handle the same situation in the future.

The other key emphasis is the basis of the relationship. If the relationship is structured well, this will reflect in the radiation therapist's professional relationships with patients and other professionals. Supervision can also provide opportunity to support the radiation therapist's self‐care, build resilience and develop strategies that improve wellbeing.

Unfortunately, after returning from maternity leave, I experienced rock bottom in my career and workplace support and taking leave were my only options. I was fortunate enough to bounce back and now feel passionate that professional supervision could prevent other radiation therapists going through what I went through.

I aim to share our professional supervision experience at Christchurch Oncology, plans we have for the future, while sharing a little of my wellbeing journey along the way.

## The ongoing impact of COVID‐19 on the clinical education of Australian medical radiation science students

### Adam Steward,^1^ Steven Lacey,^2^ Nigel Anderson,^3^ Kenton Thompson,^4^ Christopher Parsons,^5^ Amy Gray^2,6^

#### ^1^Western Health, Footscray, Australia ^2^Royal Children's Hospital, Parkville, Australia ^3^Olivia Newton‐John Cancer Wellness and Research Centre, Melbourne, Australia ^4^Peter MacCallum Cancer Centre, Melbourne, Australia ^5^Barwon Medical Imaging, Geelong, Australia ^6^The University of Melbourne, Parkville, Australia

**Introduction:** Clinical education is crucial to develop the skills of future medical radiation science (MRS) practitioners. Thus, the impact of COVID‐19 on clinical education warrants further investigation to assess the extent of any underdeveloped skills of students.^1–3^ The perspective of academics and students has been well documented globally.^3^ This paper reports on MRS clinical educators' perspectives within Australia.

**Methods:** MRS clinical educators across Australia were invited to complete an ethics approved survey between 17 June to 17 July 2022. Survey questions related to students' competency, wellbeing and safety using the Medical Radiation Practice Board of Australia's professional capability standards.

**Results:** 55 clinical educators replied to the survey with 26 from medical imaging and 29 from radiation therapy. Respondents reported that first‐ and second‐year students had the greatest lost placement time, with 65% and 61% of students losing more than a quarter of their clinical placements, respectively. Domain 1 (Medical radiation practitioner) and Domain 3 (Communicator and collaborator) of the Medical Radiation Practice Board of Australia's standards demonstrated the greatest negative impact from reduced placement time.

Prior to the pandemic, students were receiving adequate opportunities according to respondents (95%) but did not gain the required experience during the pandemic (14%); 58% reported that students were underprepared to enter the workforce.

**Conclusion:** Ongoing concerns remain among MRS clinical educators around the quality and preparedness of students due to pandemic related loss of placement time and learning opportunities. A greater understanding of this impact allows educators to better support the transition to practice of MRS practitioners.

**References**

1. McNulty JP, England A, Shanahan MC. International perspectives on radiography practice education. Radiography 2021;27(4):1044–51.
2. Sreedharan S, Mian M, McArdle DJ, Rhodes A. The impact of the COVID‐19 pandemic on diagnostic imaging services in Australia. J Med Imaging Radiat Oncol 2022;66(3):377–84.
3. Elshami W, Abuzaid MM, McConnell J, et al. The impact of COVID‐19 on the clinical experience and training of undergraduate student radiographers internationally: the clinical tutors' perspective. Radiography 2022;28:S59–67.

## Sunday 30 April, 9:00 AM–10:30 AM

## Forensic Radiography – MI

## Beyond the skeletal survey: how modern neuroimaging is revealing the effects of childhood maltreatment

### Gary Denham^1^

#### ^1^Hunter New England Health, Taree, Australia

It has been well established that childhood maltreatment or abuse, has detrimental effects on mental health and is a major risk factor for most psychiatric disorders. Maltreatment can also lead to a range of behavioural problems and can alter the structure and function of the developing brain. It is also associated with impairments to IQ, academic achievement, working memory, emotional regulation and inhibitory control.^1^

Modern neuroimaging techniques, such as functional MRI, positron emission tomography and diffusion tensor imaging have the ability to visualise the structural changes that occur in the brain due to child maltreatment. Examples include the ability of functional MRI to visualise the altered facial perception of adults in people with a history childhood emotional maltreatment and diffusion tensor imaging to detect abnormalities of white matter microstructure in the brains of maltreated children.^2^

This presentation reviews the chronic stress response to childhood maltreatment and the subsequent effects on the developing brain. This presentation also explores current neuroimaging techniques for child abuse and how medical imaging is changing the way child abuse and its resultant behaviours is understood by researchers.

**References**

1. Hart H, Rubia K. Neuroimaging of child abuse: a critical review. Front Hum Neurosci 2012;6.
2. Cassiers LLM, Sabbe BGC, Schmaal L, et al. Structural and functional brain abnormalities associated with exposure to different childhood trauma subtypes: a systematic review of neuroimaging findings. Front Psychiatr 2018;9.

## Medico‐legal aspects of non‐accidental injury imaging

### Edel Doyle^1,2^

#### ^1^Lumus Imaging, Melbourne, Australia ^2^International Association of Forensic Radiographers, ANZ Branch, Australia

Forensic imaging is ‘the application of the science of diagnostic imaging to questions of law’.^1^ A skeletal survey X‐ray series in the investigation of non‐accidental injury is performed for medicolegal purposes, rather than clinical reasons. Therefore, the medicolegal aspects of providing an imaging service should be considered by the radiographers involved in the skeletal survey X‐ray series. These include continuity of evidence, appropriate witness and documentation of the examination.

This presentation will review the recently published Royal Australian and New Zealand College of Radiologists Guideline for Imaging of Suspected Non‐Accidental Injury, in conjunction with the International Association of Forensic Radiographers best practice guidelines for the provision of forensic imaging.^1,2^

**References**

1. Doyle E, Hunter P, Viner MD, et al. IAFR Guidelines for best practice: principles for radiographers and imaging practitioners providing forensic imaging services. Forensic Imaging 2020;22:200400.
2. The Royal Australian and New Zealand College of Radiologists. Guideline for imaging of suspected non‐accidental injury. 2022. Available at https://www.ranzcr.com/documents-download/professional-documents/guidelines.

## Sunday 30 April, 11:00 AM–12:30 PM

## Professional Pathways – Combined

## Medical radiation practitioner regulation in New South Wales: key learning points and case studies

### Nadine Thompson^1^

#### ^1^Medical Radiation Practice Council of New South Wales, Sydney, Australia

The Medical Radiation Practice Council (MRPC) of New South Wales manages complaints about the conduct, performance and health of registered medical radiation practitioners and students in New South Wales with the key objective of protecting the public.^1^ The MRPC is made up of six council members (four practitioner, one community and one legal member) and is supported by staff at the Health Professional Councils Authority.

The MRPC receive complaints from the Health Care Complaints Commission and the Australian Health Practitioner Regulation Agency (Ahpra) for practitioners and students based in New South Wales. Complaints are managed by the MRPC across three key pathways: performance, health and conduct.

This presentation will outline the responsibilities of the MRPC and provide insight into the types of complaints managed over the past few years, with some anonymised case study examples from all three key pathways, including examples of complaints that require immediate action under Section 150 of the National Law (NSW).^1^ The presentation will also include what happens to a practitioner when conditions on their registration are required to ensure safe practice.

This presentation will be helpful for all practitioners and students as they will be able to learn from the experience of others and understand the present complaints management process in New South Wales to better shape their future. This presentation will provide attendees with a firm understanding about what kinds of complaints are received and how the MRPC manages these complaints to champion change: supporting practitioners to improve public safety.

**Reference**

1. Medical Radiation Practice Council of New South Wales. Available at https://www.medicalradiationpracticecouncil.nsw.gov.au.

## Maximising all that hot air: DIBH vs non‐DIBH in adolescent young adult patients

### Danielle Duff^1^

#### ^1^Te Whatu Ora Waitaha: Canterbury Regional Cancer & Haematology Service, New Zealand

**Introduction:** Should deep inspiration breath hold (DIBH) be used in adolescent young adult patients? This case study reviews the use of DIBH verses free breathing to improve patient outcomes for a 13‐year‐old patient with a chest wall sarcoma.

**Case Presentation:** This presentation will provide a brief review of the literature, including international recommendations, and how we were able to adapt clinical protocols to achieve the best outcome for a young person during their treatment. It will assess the educational requirements needed to achieve DIBH compliance in a 13‐year‐old as well as the additional dosimetric considerations required to facilitate treatment in breath hold.

**Discussion:** Discussions will include a plan comparison between DIBH and free breathing and the clinical considerations made to determine the best plan for this young person.

## Assessment of workplace learning in MRS: Why? What? How?

### Andrew Kilgour^1^

#### ^1^RMIT University, Bundoora, Australia

**Objectives:** Proving capability in workplace learning requires an assessment approach which considers the contextual variability of professional practice. The aim of this research was to develop an approach to assess undergraduate radiography students' workplace learning capability.^1^

**Methods:** This study is located in the interpretive paradigm, within which a deconstruction‐reconstruction method was employed. The overarching research question was: What is an optimal method for assessing the clinical capability of radiography students? To answer this question three research sub‐questions were posed:

1. Why do we asses?
2. What do we assess?
3. How do we assess it?

**Results:** This study clearly pointed to different assessment strategies being necessary for assessing professional practice. Because professional practice does not occur in isolation, but rather forms part of a complex whole, it was determined that the different assessment strategies should occur in parallel, producing a multi‐dimensional assessment approach.^2^ This approach assesses student capability in professional practice utilising professional judgement of workplace assessors, structured reflective journals assessed by university academics, and assessment of technical competence by workplace assessors.

**Discussion:** The deconstruction process examined the nature of professional practice in the literature; it was deemed to be interactional, dynamic and contextual in nature. These aspects of professional practice were found to be integral to assessment of radiography workplace learning capability, but not to the neglect of the technical aspects of radiography practice currently assessed. Therefore, the assessment approach developed needed to adequately assess all these aspects of practice.^3^

**References**

1. Kilgour A. Radiography assessment for practice: a critical practice enquiry. 2018.
2. Yorke M. Work‐engaged learning: towards a paradigm shift in assessment. Qual High Educ 2011;17(1):117–30.
3. Sadler DR. Indeterminacy in the use of preset criteria for assessment and grading. Assess Eval High Educ 2009;34(2):159–79.

## Professional certification – embrace the present to shape your future

### Min Ku^1^

#### ^1^Australian Society for Medical Imaging and Radiation Therapy, Melbourne, Australia

The Australian Society of Medical Imaging and Radiation Therapy (ASMIRT) is the peak body representing medical radiation practitioners in Australia. Currently ASMIRT offers a broad range of certification options available to all medical radiation practitioners in Australia and internationally. These certifications have been available for at least two decades. The certification pathway varies between the certification types, however, includes a theoretical component and a practical component. Medical radiation practitioners are required to meet a minimum regulatory standard to practise their specific modality.^1^

For practitioners to demonstrate additional skills, knowledge and expertise in their chosen modality, there is a choice of further postgraduate education, or alternatively continuing professional development through an industry recognised professional certification.

The certification program is designed to assist medical radiation practitioners with their career pathways. It enables a smaller manageable form of study in a specific modality to support that scope of practice. Investing time, energy and resources demonstrates to employers a commitment to self‐improvement and excellence in your profession.^2^

This presentation will detail the benefits of the ASMIRT professional certification pathway, the opportunities available for medical radiation practitioners and the benefits of obtaining and retaining this formal professional recognition.

**References**

1. Medical Radiation Practice Board of Australia. Professional capabilities for medical radiation practice. 2022. Available at www.medicalradiationpracticeboard.gov.au/Registration-Standards/Professional-Capabilities.aspx.
2. Lockhart L. Nursing certification: “a mark of excellence”. Nursing Made Incredibly Easy! 2019;17(3):56. Available at doi: 10.1097/01.NME.0000554606.21726.05.

## Sunday 30 April, 11:00 AM–12:30 PM

## Advanced Radiotherapy Practice – RT

## Chemo‐radiotherapy induced haematological toxicity: the implementation catalyst for bone marrow sparing gynaecological radiotherapy

### Gemma Busuttil,^1^ Niluja Thiruthaneeswaran,^1,2^ Jennifer Chard,^1^ Alison Salkeld^1,2^

#### ^1^Sydney West Radiation Oncology Network, Westmead, Australia ^2^The University of Sydney, Westmead, Australia

Acute haematological toxicity is a known side effect of chemo‐radiotherapy with an incidence of 30–45% when fields encompass active bone marrow (ABM). However, bone marrow is not routinely defined as an organ at risk during planning.

In 2019, one cervical cancer patient receiving extended field chemo‐radiotherapy (EFC‐RT) experienced grade 3 anaemia and thrombocytopaenia, alongside grade 4 leukopaenia, neutropaenia and lymphopaenia. This required more than 20 blood product transfusions, a month‐long hospital admission and multiple treatment breaks. This highlighted a quality improvement need to reduce haematological toxicity for future EFC‐RT patients.

Bone marrow contouring definitions and dose constraints were determined from a literature review. A planning study utilised three previous patient datasets, with CT‐based bone marrow surrogate and PET‐based ABM contoured. Plans were optimised with bone marrow and ABM constraints then compared with the original. Bone marrow optimised plans reduced bone marrow V10, V20, V30 and V40 by 13%, 10%, 3% and 1%, respectively, and ABM by 10%, 11%, 5% and 3%, respectively, while maintaining coverage of target volumes. ABM optimised plans reduced ABM doses by 13%, 17%, 18% and 15%, respectively, however target coverage was compromised.

CT‐based bone marrow sparing was selected for expedited implementation. Gynaecological planning guidelines were updated to include the bone marrow objectives, and a progressive implementation followed over a 24‐month period as follows: (i) EFC‐RT patients, (ii) concurrent chemotherapy patients, (iii) all gynaecological patients. Retrospective data collection of full blood count and blood product transfusion is ongoing. Future quality improvement includes reassessing PET‐based ABM sparing, investigating MRI‐based ABM delineation, and identifying biological predictors for haematological toxicity.

## Predicting normal brain dose in hypofractionated stereotactic radiotherapy for single brain metastases using VMAT

### Erin Johns,^1^ Cathy Hargrave,^1,2^ Lisa Nissen,^2^ Mark Pinkham,^1^ Anne Bernard,^3^ Tamara Barry^1^

#### ^1^Princess Alexandra Hospital, Brisbane, Australia ^2^Queensland University of Technology, Brisbane, Australia ^3^Queensland Cyber Infrastructure Foundation, Brisbane, Australia

**Objectives:** Hypofractionated stereotactic radiotherapy (HF‐SRT) is used to treat brain metastases.^1^ Metrics for constraining intermediate/high dose are well established, however, there are no standardised goals for lower doses.^2^ It is difficult to determine whether greater sparing of normal brain can be achieved during optimisation, which can be time‐consuming. The aim of this research was to develop predictive models of optimal low‐dose to normal brain to aid generating single brain metastases HF‐SRT plans.

**Methods:** A retrospective review of 90 single brain metastases HF‐SRT plans was conducted to assess dose to normal brain and determine technical (modifiable) and patient‐related (fixed) components of the planning process. Exploratory analyses were performed to determine significant variables for inclusion in model development. Multiple linear regression was used to model healthy brain dose.

**Results:** The average planning target volume (PTV) was 30.26 cm^3^ (1.41 cm^3^–173.98 cm). The average normal brain mean dose was 2.52 Gy (range = 0.62 Gy – 5.12 Gy). The average volume of normal brain receiving 50% and 25% of the prescribed dose was 40.65 cm^3^ (5.86 cm^3^–111.59 cm^3^) and 127.53 cm^3^ (15.96 cm^3^–365.63 cm^3^), respectively.

Models for predicting mean brain dose and the volume of the brain receiving 50% and 25% of the prescribed dose were produced containing five statistically significant variables: PTV, PTV location, PTV shape, treatment arc combinations and floor angles. Each model explains between 71.2% to 78.2% of normal brain dose and overall significance of the models was P < 0.001.

**Discussion/Conclusion:** This is the first study to model low‐dose for patients with single brain metastases HF‐SRT plans. These models are currently being evaluated for clinical implementation.

**References**

1. Ruschin M, Lee Y, Beachey D, et al. Investigation of dose falloff for intact brain metastases and surgical cavities using hypofractionated volumetric modulated arc radiotherapy. Technol Cancer Res Treat 2016;15(1):130–8.
2. Shaw E, Scott C, Souhami L, et al. Single dose radiosurgical treatment of recurrent previously irradiated primary brain tumours and brain metastases: final report of RTOG protocol 90–05. Int J Radiat Oncol Biol Phys 2000;47(2):291–8.

## Deep learning‐based metal artefact reduction in CT scans for ultrasound‐guided cardiac radioablation

### Sathyathas Puvanasunthararajah,^1,2^ Saskia Camps,^3^ ML Wille,^2,4,5^ Davide Fontanarosa^1,2^

#### ^1^School of Clinical Sciences, Queensland University of Technology, Brisbane, Australia ^2^Centre for Biomedical Technologies, Queensland University of Technology, Brisbane, Australia ^3^EBAMed SA, Geneva, Switzerland ^4^School of Mechanical, Medical & Process Engineering, Queensland University of Technology, Brisbane, Australia ^5^ARC Training Centre for Multiscale 3D Imaging, Modelling and Manufacturing, Queensland University of Technology, Brisbane, Australia

**Objective:** Cardiac radioablation is a promising non‐invasive modality for the treatment of cardiac arrhythmias, but accurate dose delivery can be affected by heart motion. For this reason, real‐time ultrasound guidance can be a solution. A typical ultrasound‐guided cardiac radioablation workflow includes simultaneous ultrasound and planning CT acquisitions, which can result in ultrasound transducer‐induced metal artefacts on the planning CT scans. To reduce the impact of these artefacts, a metal artefact reduction algorithm has been developed based on a deep learning Generative Adversarial Network (CycleGAN), and it has been compared with metal deletion technique (MDT) and combined clustered scan‐based metal artefact reduction (CCS‐MAR) algorithms.^1,2^

**Methods:** COVID‐19‐CT dataset was used in this study.^3^ The CycleGAN network was trained using CT scans from 70 patients consisting of paired CT slices with and without simulated ultrasound transducer‐induced metal artefacts. Then, CT scans with simulated artefacts from 14 patients were used to create the artefact‐corrected CT scans after MDT, CCS‐MAR and CycleGAN applications. The HU value improvement percentage for the heart, lungs and bone regions in the artefact‐corrected CT scans was calculated.

**Results:** In Figure 1 an example of the effect of the application of these algorithms is shown. The proposed CycleGAN network effectively reduces the negative impact of metal artefacts. For example, the calculated HU value improvement percentage for the heart in the artefact‐corrected CT scans was 59.58%, 62.22% and 72.84% after MDT, CCS‐MAR and CycleGAN applications, respectively.

**Discussion/Conclusion:** In comparison to MDT and CCS‐MAR, the developed CycleGAN application on CT scans performs better or comparably in reducing these artefacts.


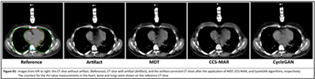


**References**

1. Boas FE, Fleischmann D. Evaluation of two iterative techniques for reducing metal artefacts in computed tomography. Radiology 2011;259(3):894–902.
2. Puvanasunthararajah S, et al. Combined clustered scan‐based metal artefact reduction algorithm (CCS‐MAR) for ultrasound‐ guided cardiac radioablation. Phys Eng Sci Med 2022;1273–87.
3. Mostafavi SM. COVID19‐CT‐Dataset: an open‐access chest CT image repository of 1000+ patients with confirmed COVID‐19 diagnosis. Harvard Dataverse. Available at doi.org/10.7910/DVN/6ACUZJ.

## Points in the matrix: can plan metrics inform automated planning models and enhance planning pathways?

### Laura Baker,^1^ Andrew Le,^1^ Regina Bromley,^1^ Susan Carroll,^1,2^ Thomas Eade,^1,2^ Brian Porter,^1^ John Atyeo^1^

#### ^1^Northern Sydney Cancer Centre, Royal North Shore Hospital, St Leonards, Australia ^2^The University of Sydney, Camperdown, Australia

**Objectives:** In this study, scoring based metric systems via ProKnow^1^ were used to inform and develop current RapidPlan^2^ knowledge‐based planning models to provide a simple and robust comprehensive planning tool.

**Methods:** A dosimetric scoring system was developed for breast and post‐mastectomy comprehensive lymph node radiotherapy patients for 50 Gy in 25 fractions^3^ with the addition of a 57 Gy simultaneous integrated boost as required. A knowledge‐based planning model was then updated with the influence of the scoring metric to further decrease the dose to surrounding organs at risk while maintaining target coverage focussing on a diminishing returns points scheme. This was applied and validated on 20 patients in comparison to the current clinical RapidPlan model used.

**Results:** Using the newly informed model, higher quality treatment plans were achieved via one single optimisation reflected in a plan score increase of 4.6% across 20 patients. The newly updated RapidPlan model showed a decrease in organs at risk dose while maintaining target coverage and the elimination of hotspots.

**Discussion/Conclusion:** A dosimetric scorecard can be used as an objective measure for plan quality resulting in higher quality plans. In conjunction with RapidPlan modelling, the scorecard metric can be used to enhance the automation workflow currently used within departments to further improve their own plans, reduce the need for replans and increase the speed of planning workflows. In future this could also be used as a comparison metric to look at the need for automated planning implementation in other centres.

**References**

1. Elekta ProKnow. Available at https://proknowsystems.com/about?content = mission.
2. Varian Medical Systems. Varian RapidPlan. Available at https://www.varian.com/en-au/products/radiosurgery/treatment-planning/rapidplan.
3. Stanton C, Bell LJ, Le A, et al. Comprehensive nodal breast VMAT: solving the low‐dose wash dilemma using an iterative knowledge‐based radiotherapy planning solution. J Med Radiat Sci 2022;69(1):85–97.

## Histology classification based on radiomics for non‐small cell lung cancer patients using an artificial neural network

### Eva YW Cheung,^1^ Wing Yan Wong,^1^ Ricky Wu,^2^ Ellie Chu^1^

#### ^1^Tung Wah College, Hong Kong ^2^Glasgow Caledonian University, Glasgow, Scotland

**Background:** Lung cancer is the most common cancer in Hong Kong, with 85% of lung cancers being non‐small cell lung cancer (NSCLC). Within NSCLC, there are large cell carcinoma, squamous cell carcinoma, adenocarcinoma and other types, which can be confirmed through pathological analysis only. In this study, we aimed to classify the above histology types based on radiomics retrieved from CT images.

**Method:** Planning CT images and RT structures of 293 NSCLC patients were downloaded from the cancer imaging archive database. 107 radiomics were retrieved from the planning CT using slicer version 4.11.20210226, based on gross tumour volume delineated by clinical oncologists. An artificial neural network model was built on Matlab neural network toolbox to classify large cell carcinoma, squamous cell carcinoma and adenocarcinoma from others for NSCLC patients. The model performance was analysed by its sensitivity, specificity, area under the ROC curve and overall accuracy.

**Results:** The proposed neural network model showed good performance in predicting different types of histology, with overall accuracy of 74.2% and area under the ROC curve of 78%. The sensitivity and specificity of large cell carcinoma are 67% and 83.1%, squamous cell carcinoma 79% and 79.8%, adenocarcinoma 72.4% and 67.7%, and others 78.7% and 587%, respectively.

**Conclusion:** CT radiomics had good performance in classifying different types of histology in NSCLC patients using a neural network model. External cohort of patients can be included to validate the model for further studies.


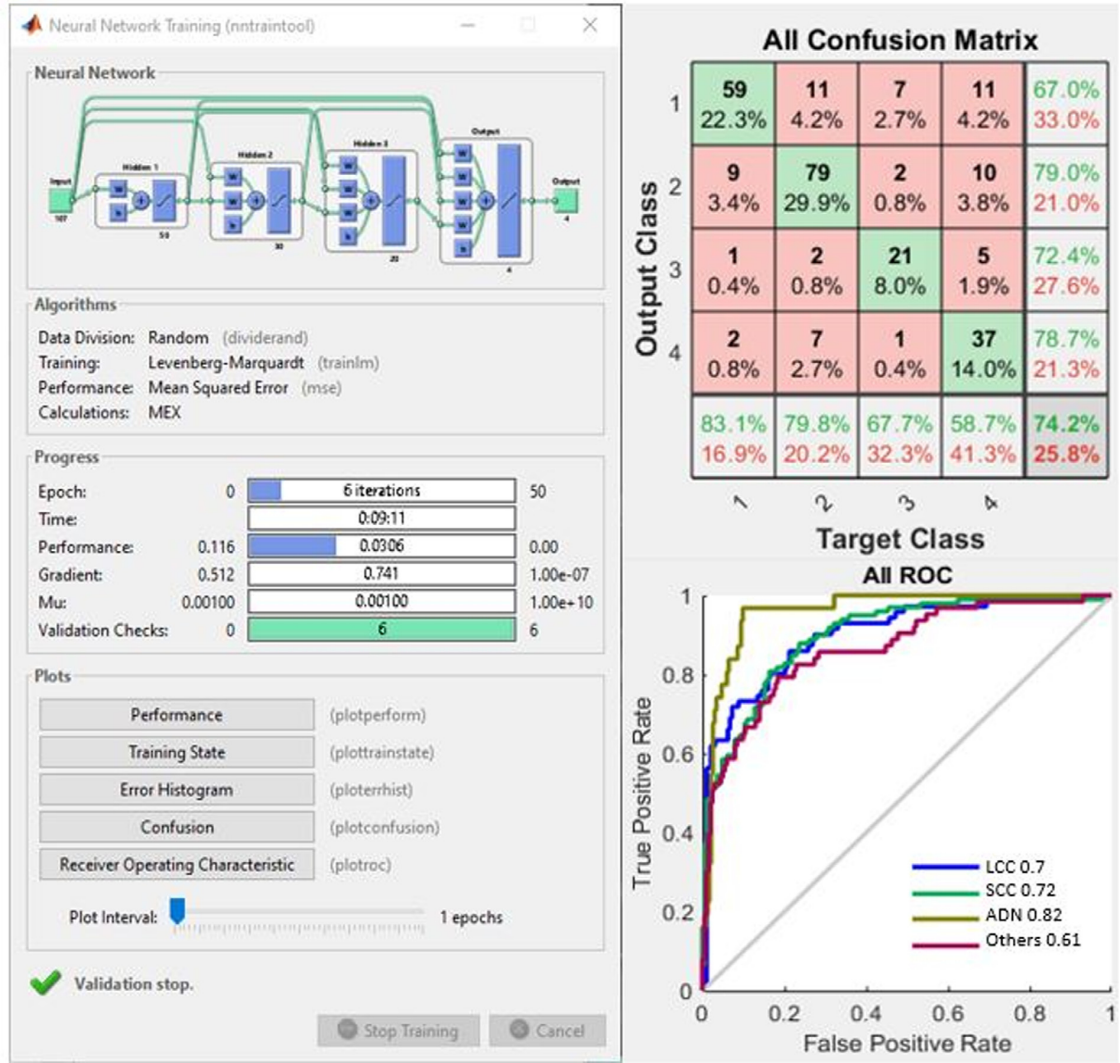


## Sunday 30 April, 11:00 AM–12:30 PM

## General Radiography 2 – MI

## Are we following the evidence? An audit of paediatric forearm protocols

### Annabel Keir^1^

#### ^1^The Prince Charles Hospital, Brisbane, Australia

**Objectives:** At a Queensland tertiary hospital, a child aged less than 12 years who presents with an acute wrist injury will receive a full forearm X‐ray as per paediatric X‐ray protocols. With no known origin of this protocol, the evidence has revealed that doctors fear missing concurrent injuries at the elbow, however, existing literature states it is an unnecessary use of radiation.^1^ This audit aimed to find the prevalence of proximal forearm fractures in children with wrist pain.

**Method:** A retrospective study of 536 forearm X‐rays over a six‐month period (January to June 2022) were reviewed and included if a child was aged less than 12 years, with an acute wrist injury, presenting to emergency. Data was collected from radiology reports and clinical notes to identify prevalence.

**Results:** Out of 278 eligible participants, 154 children had confirmed distal forearm fractures and six (2.16%) had mid‐proximal forearm fractures. Out of those six children, four had obvious visual deformities of the forearm, that likely would not have been missed by a clinician. Which lowers the prevalence of proximal fractures (without visual deformity) to 0.7%.

**Conclusion:** The prevalence of proximal forearm fractures in children aged less than 12 years with wrist pain presenting to the emergency department is very low. Undertaking forearm radiographs for wrist pain is an unnecessary additional use of radiation on our most radiosensitive population. It is recommended the protocol is abolished and suggested if a visual deformity is present, then a forearm X‐ray is appropriate.

**Reference**

1. Golding LP, Yasin Y, Singh J, et al. Imaging of the elbow in children with wrist fracture: an unnecessary source of radiation and use of resources? Pediatr Radiol 2015;45(8):1169–73.

## Championing change: improving emergency department efficiencies by determining unnecessary representations to the medical imaging department

### Hannah To,^1^ Michael Neep^1^

#### ^1^Logan Hospital, Meadowbrook, Australia

**Objectives:** Inefficiencies within emergency departments are widely recognised and have negative implications to patient outcomes.^1^ Modelling has shown that decreasing medical imaging turnaround times improves patient throughput and decreases patient waiting time.^2^ The purpose of this study was to identify the number of representations to the medical imaging department that could have been avoided.

**Methods:** Stage one examined a randomised sample of 400 patients who received imaging during their emergency department presentation in January 2021. This identified the number of patient representations to the medical imaging department. Stage 2 involved two medical officers independently reviewing each of the patient's electronic medical record (identified in stage 1 data) against two criteria to ascertain, whether their representation to the medical imaging department was justified.

**Results:** Of the 400 patients reviewed, 59 (14.75%) were identified as having more than one presentation to the medical imaging department. Only 21 (35.59%) of the 59 cases where a representation occurred was deemed necessary and on 38 (64.41%) occasions, the patient was deemed to have an avoidable representation to the medical imaging department.

**Discussion/Conclusion:** In a one‐month period, 64.41% of patients reviewed were identified to have unnecessary representations to the medical imaging department. Extrapolating this data to one year equates to approximately 4104 patient visits to the medical imaging department deemed avoidable. This finding suggests that if the initial patient assessment yielded the correct request for medical imaging, the overall efficiency of the medical imaging pathway could be improved. By championing change and recognising areas of improvement, the efficiency of the entire patient pathway within the emergency department can be improved.

**References**

1. Guittard JA, Wardi G, Castillo EM, et al. Grow the pie: interdepartmental cooperation as a method for achieving operational efficiency in an emergency department. J Emerg Med 2018;55(2):269–77.
2. Paul JA, Lin L. Models for improving patient throughput and waiting at hospital emergency departments. J Emerg Med 2012;43(6):1119–26.

## Anti‐scatter grid versus scatter correction software in digital radiography of the adult pelvis

### Greg Trypis,^1^ Lauren Elliott^1^

#### ^1^Sunshine Coast Hospital and Health Service, Birtinya, Australia

**Objectives:** To compare the image quality and any potential radiation exposure reduction of radiographic images of the adult pelvis acquired with the use of an anti‐scatter grid or use of contrast enhancement scatter correction software.

**Methods:** Radiographic images of the adult pelvis acquired with either the use of an anti‐scatter grid or contrast enhancement software were retrospectively evaluated for their image quality using two independent radiologists. For the evaluation of any potential radiation dose reduction, an adult anthropomorphic phantom pelvis was used to create radiographic images using both an anti‐scatter grid and contrast enhancement scatter correction software. Respective radiation doses were calculated using appropriate radiation dose monitoring equipment.

**Results:** Although the results of this activity are still in the process of being determined, it is our hypothesis that images acquired with the use of an anti‐scatter grid will demonstrate superior image quality compared to images acquired with contrast enhancement scatter reduction software. Similarly, our hypothesis is that the resultant radiation dose will be higher for the images acquired with the use of an anti‐scatter grid compared to the images acquired with contrast enhancement software.

**Conclusion:** This activity aims to shape our future in the use of either anti‐scatter grid or contrast enhancement software in imaging of the adult pelvis. The hypothesised potential loss of image quality with the use of contrast enhancement software may still be an acceptable compromise if the hypothesised radiation dose savings are significant.

## Making an impact as an advanced practitioner

### Don Nocum^1^

#### ^1^The University of Sydney/Sydney Adventist Hospital, Sydney, Australia

The advanced practice model for diagnostic radiographers and radiation therapists is an approach to progress the profession and to meet the current and future demands of the Australian healthcare system. It is both profession‐led and patient‐focussed.^1^ As defined by the Australian Society of Medical Imaging and Radiation Therapy (ASMIRT), the key characteristics of an advanced practitioner involve expert communication, internal and external collaboration, exhibiting a high level of professionalism, involvement in scholarship and teaching, and possessing advanced clinical expertise, professional judgement and clinical leadership.^2^ There is a need for medical imaging professionals to undertake extended roles to support patient care delivery and contribute to a multi‐disciplinary framework.

This presentation is intended to inform on the contributions of Advanced Practice in diagnostic radiography, using its application within the specialty of angiography and interventional radiology as the primary example. Synonymous to filling in the gaps in the literature for an original research article, the advanced practitioner has the capacity to fill and extend their role within a department to contribute their clinical expertise for the benefit of patients in their healthcare setting. The professional work and scientific contributions of an advanced practitioner has direct impact on the quality of care for patients and its services. Evidence of the advanced practice model in effect at a local hospital level will be explored.

**References**

1. Smith T. Advanced practice – profession‐led and patient‐focused. The Radiographer 2009;56(3):4–5.
2. Advanced Practice Advisory Panel (APAP). Pathway to advanced practice: advanced practice for the Australian medical radiation professions. Australian Society of Medical Imaging and Radiation Therapy. 2017. Available at https://www.asmirt.org/asmirt_core/wp‐content/uploads/131.pdf.

## Quality of X‐ray request form information for acute shoulder injuries at an Australian tertiary hospital

### Mark Metcalfe,^1^ Mark Wilcox,^1^ Karen Doheny,^1^ Karen Dobeli^1^

#### ^1^Royal Brisbane and Women's Hospital & STARS, Herston, Australia

**Objectives:** The aim of this audit was to evaluate the quality of clinical information provided by referrers on request forms for X‐ray imaging of the acute shoulder at a major city hospital.

**Methods:** Institutional ethics board approval was obtained. X‐ray request forms for 500 consecutive adult patients with acute shoulder pain/injury referred from the emergency unit were assessed for quality of clinical information provided by the referrer. Clinical information was assessed for two components (mechanism of injury, and signs/symptoms/location of pain), which were categorised as either ‘not provided’, ‘minimal’ or ‘detailed’ according to predetermined criteria.

**Results:** Clinical information regarding mechanism of injury was classed as ‘not provided’, ‘minimal’ and ‘detailed’ for 64 (13%), 64 (13%) and 372 (74%) request forms, respectively. Signs and symptoms were classed as ‘not provided’, ‘minimal’ and ‘detailed’ for 183 (37%), 162 (32%) and 155 (31%) X‐ray requests, respectively.

**Discussion/Conclusion:** Most acute shoulder X‐ray requests assessed provided a detailed history of injury mechanism. However, less than one‐third of requests provided a clear indication of the location and nature of signs and symptoms. Poor quality of clinical information provided by referrers can lead to inadequate or inappropriate selection of X‐ray views by the radiographer and, subsequently, reduced accuracy of the radiology report. Various methods for improving clinical information on imaging requests have been reported (e.g. referrer training, electronic requests with forced data entry). Our department is undertaking a trial of a clinical assessment tool for radiographers to obtain the required information directly from the patient.

## Providing patient‐centred care as medical radiation practitioners to children in out of home care

### Gary Denham,^1^ Sharon Denham^2^

#### ^1^Hunter New England Health, Taree, Australia ^2^Wesley Mission, Taree, Australia

Modern health care focusses on patient‐centred care where patients' needs, beliefs, choices and preferences are valued and lead to better health outcomes. Children and young persons in out of home care require more healthcare services compared with children from similar social and economic backgrounds.^1^

Complex trauma is the prolonged and uncontrolled exposure to traumatic events, such as that experienced by maltreated children. Complex trauma can create a toxic stress response that produces biological alteration to the developing brain and affects the lives of the child, other family members and their descendants.^2^

Children with complex trauma often do not have the ability to regulate their responses to stimuli, reacting to minor triggers with disproportionate reactions. Many of these children will present with challenging behaviours.

Trauma‐informed care is a method of service delivery that seeks to actively minimise re‐traumatisation. Creating a safe space is an essential element of trauma‐informed care. Children with a history of complex trauma have life experiences that may be re‐lived in a healthcare setting.^3^

This presentation will outline the ethical and legal considerations such as privacy, consent and mandatory reporting when dealing with children in out of home care. The concepts of trauma‐informed care will be highlighted to assist medical radiation practitioners minimise further trauma to one of the most vulnerable population groups in Australia.

**References**

1. Rathert C, Wyrwich MD, Boren SA. Patient‐centered care and outcomes: a systematic review of the literature. Med Care Res Rev 2013;70(4):351–79.
2. Bucci M, Marques SS, Oh D, Harris NB. Toxic stress in children and adolescents. Adv Pediatr 2016;63(1):403–28.
3. Bendall S, Eastwood O, Cox G, et al. A systematic review and synthesis of trauma‐informed care within outpatient and counselling health settings for young people. Child Maltreat 2021;26(3):313–24.

## Sunday 30 April, 11:00 AM–12:30 PM

## Pick and Mix – MI

## The diagnostic accuracy of computed tomography and ultrasound for acute appendicitis

### Elio Arruzza,^1^ S Milanese,^1^ LSK Li,^1^ J Dizon^2^

#### ^1^University of South Australia, Adelaide, Australia ^2^Flinders University, Adelaide, Australia

**Objectives:** Acute appendicitis is the most common cause of emergency surgery in Australia.^1^ The imaging diagnosis of acute appendicitis remains challenging, but its role is imperative in reducing unnecessary surgical rates, associated risks and expenses. A major issue experienced by clinicians is a deficiency of clear evidence‐based guidelines or consensus as to the indications for a particular modality or protocol.^2^ This review determined the current diagnostic accuracy of CT and ultrasound for suspected acute appendicitis in adults.

**Methods:** A systematic search was undertaken in appropriate databases. Screening of potential titles and abstracts, full‐text retrieval, methodological quality assessment and data extraction was performed. Meta‐analyses were performed for relevant subgroups. The performance of protocols such as non‐contrast, low‐dose, intravenous contrast and intravenous plus oral contrast CT, and ultrasound were statistically and visually compared.

**Results:** CT with intravenous plus oral contrast enhancement yields statistically significantly greater diagnostic accuracy than CT with intravenous contrast alone. Low‐dose CT yields comparable sensitivity and specificity to standard‐dose CT. Ultrasound studies which exclude equivocal results may overinflate sensitivity and specificity values.

**Discussion/Conclusion:** The updated diagnostic test accuracies of CT, ultrasound and relevant subgroups should be implemented in light of factors such as dose, cost and timing. While low‐dose CT is less accurate than higher‐radiation and more invasive contrast‐enhanced CT, its benefits may outweigh the low diagnostic accuracy of ultrasound. We believe the findings of this study will be invaluable in shaping future decisions for imaging technologists, emergency physicians and radiologists.

**References**

1. Australian Institute of Health Welfare. Admitted patient care 2014–15: Australian hospital statistics. Canberra: Australian Government; 2016.
2. Di Saverio S, Podda M, De Simone B, et al. Diagnosis and treatment of acute appendicitis: 2020 update of the WSES Jerusalem guidelines. World J Emerg Surg 2020;15(1).

## Development of an online clinical placement assessment for medical imaging students

### Susan Said,^1^ Frances Gray,^1^ Laura di Michele,^1^ Yobelli Jimenez,^1^ Amanda Punch^1^

#### ^1^The University of Sydney, Camperdown, Australia

**Objectives:** The professional capabilities of medical radiation practice are well established as the standard of practice that all practitioners must meet to be registered with the Medical Radiation Practice Board of Australia (MRPBA), part of The Australian Health Practitioner Regulation Agency (Ahpra).^1^ The aim of this presentation is to report on the development and initial evaluation of the new clinical assessment tool (NCAT) developed for students at an Australian university based on the MRPBA capabilities.

**Method:** The NCAT was developed with the intention to align students' clinical performance with the MRPBA capabilities, and to ensure straightforward access and ease of use on a digital platform. A first draft of the NCAT was developed by matching the criteria under each MRPBA domain with each work integrated learning unit of study's learning objectives. This was followed by an iterative process of trialling the tool in four clinical sites and making adjustments to wording and rating scale grading scales based on clinical educators' feedback.

Following implementation of the final version of the NCAT, an online survey was used to explore clinical educators' perceptions of using the NCAT to evaluate students' skills.

**Results:** The NCAT consists of five sections which align to the MRPBA domains. A manual was developed with observational descriptors on each rating. Data from online survey is currently being analysed and will be presented at the conference.

**Discussion/Conclusion:** The NCAT provided an opportunity to consult and engage with clinical supervisors as partners in student education. Development of the NCAT provides strong evidence for course accreditation purposes.

**Reference**

1. Medical Radiation Practice Board of Australia. Professional capabilities for medical radiation practice. 2020. Available at https://www.medicalradiationpracticeboard.gov.au/Registration-Standards/Professional-Capabilities.aspx.

## Shaping the future of occupational health and safety in medical radiation professions

### Tanya Barnes,^1^ Mary‐Anne Miller,^1^ Min Ku^2^

#### ^1^Metro South Health, Brisbane, Australia ^2^Australian Society for Medical Imaging and Radiation Therapy, Melbourne, Australia

**Objectives:** Across allied health, the exposure to both physical and psychosocial factors are contributors to the development of work‐related musculoskeletal disorders and injuries.^1^ Medical radiation practitioners engage with physically demanding work daily, with international studies highlighting 70% of staff reported experiencing symptoms of musculoskeletal pain and repetitive stress injury.^2^ The most common cause of these symptoms is performing patient transfers, wearing lead aprons and moving heavy equipment and chairs.^3^ This research investigates the attitudes and opinions of medical radiation practitioners towards occupational work health and safety issues within the workplace and prevalence of musculoskeletal disorders and injuries encompassing all medical radiation practitioner disciplines.

**Methods:** An ethics approved quantitative online survey with validated measurement tools was conducted during 2022. The survey was distributed to all registered Australian medical radiation practitioners. Following this, a series of targeted qualitative interviews were conducted to ascertain perceptions, feelings and experiences of medical radiation practitioners in the workplace.

**Results:** Quantitative data was collected from 257 respondents on musculoskeletal issues, occupational work health and safety and types of hazards encountered in the workplace including clinician experiences. The following demographics were also captured: age, gender, profession, qualifications, years working, hours of work, types of shifts, percentage of direct patient contact, and public or private organisations.

**Discussion/Conclusion:** The quantitative results of this study will be presented, with recommendations for improvements to occupational work health and safety practice in the medical radiation practitioner workplace.

**References**

1. Anderson SP, Oakman J. Allied health professionals and work‐related musculoskeletal disorders: a systematic review. Saf Health Work 2016;7(4):259–67.
2. Brusin JH. Ergonomics in radiology. Radiol Technol 2011;83(2):141–57.
3. Shubayr N, Alashban Y. Musculoskeletal symptoms among radiation technologists in Saudi Arabia: prevalence and causative factors. Acta Radiologica 2022;63(4):497–503.

## RESPite – reducing sonographer pain through ergonomics education

### Kristie Sweeney,^1,2^ Martin Mackey,^2^ Jacqueline Spurway,^3^ Jillian Clarke,^2^ Karen Ginn^2^

#### ^1^Bathurst Health Service, Bathurst, Australia ^2^The University of Sydney, Camperdown, Australia ^3^Orange Health Service, Orange, Australia

**Background:** Globally, multiple studies have demonstrated a high prevalence of work‐related musculoskeletal disease in sonographers. Rates of sonographers scanning with pain are reported to be between 65% to 98% and the shoulder is reported as one of the most frequently painful sites.^1^ It is estimated that one in five sonographers will suffer a career ending musculoskeletal injury.

Peak sonographer bodies advocate ergonomics approaches to reduce work‐related musculoskeletal disease with the maintenance of neutral posture during scanning central to the recommendations. However, there is a paucity of high‐quality studies evaluating interventions to educate sonographers in best practice ergonomics.^2,3^

**Methods:** RESPite (Reducing Sonographer Pain through ergonomics education) is an educational resource designed using established ergonomic principles recommended by peak sonographer bodies. It offers guidance on risk factor identification and simple ergonomic strategies a sonographer can apply during each examination to scan with optimal musculoskeletal postural safety. A grant from the Australasian Sonographers Association was used to develop this training resource for sonographers.

**Results:** The educational program, RESPite, was piloted to a small group of sonographers with predominately positive reviews, however, changes were suggested and implemented following the pilot review. The online ergonomics program is being used in a randomised control trial to ascertain the effectiveness of RESPite in preventing or decreasing sonographer work‐related musculoskeletal disease.

**Implications:** If targeted education in ergonomic best practice reduces work‐related musculoskeletal disease in sonographers, it would be an easy and cost‐effective intervention, that would improve sonographer health and workability and benefit employees and employers by reducing sick leave and improving productivity.

**References**

1. Feng Q, Liu S, Yang L, Xie M, Zhang Q. The prevalence of and risk factors associated with musculoskeletal disorders among sonographers in central China: a cross‐sectional study. PLoS ONE 2016;11(10):e0163903.
2. Schoenfeld A, Goverman J, Weiss DM, Meizner I. Transducer user syndrome: an occupational hazard of the ultrasonographer. Eur J Ultrasound 1999;10(1):41–5.
3. Fisher TF. Radiologic and sonography professionals' ergonomics: an occupational therapy intervention for preventing work injuries. J Diagn Med Sonography 2015;31(3):137–47.

## See something, say something: the coroner's perspective

### Kristal Lee,^1,2^ Kriscia Tapia,^1^ Mo'ayyad Suleiman,^1^ Catherine Jones,^1,2,3^ Patrick Brennan,^1^ Ernest Ekpo^1^

#### ^1^The University of Sydney, Sydney, Australia ^2^Monash University, Melbourne, Australia ^3^I‐MED Radiology, Brisbane, Australia

The Medical Radiation Practice Board of Australia's Communicating Safely Policy, also known as See Something, Say Something, outlines that if a medical radiation practitioner identifies urgent or unexpected findings, they must communicate this information in a timely manner to the appropriate healthcare practitioner.^1^

This policy was born from coronial inquiries involving the untimely death of patients due to errors or result delays in medical imaging. Recommendations from the coroner in 2013 were for skilled radiographers to alert medical staff to a ‘clear and significant issue relating to patient safety’.^2^ This was then highlighted again by another untimely and avoidable death – this time of a child – in 2015 where the coroner reiterated the need for early clinical notification of significant findings by radiographers. In 2017, radiographers were again reminded by the coroner that unexpected, urgent, or sinister radiological findings should be the subject of immediate communication to the referring medical practitioner.^3^ This was also in relation to an avoidable death.

The aim of this work is to present the historical coronial inquiries of three patients, and the coroners' subsequent recommendations that are reflected in the See Something, Say Something capabilities. As medical radiation practitioners gain a greater understanding of the origin of these professional capabilities, they will be able to better meet their present requirements. This will empower medical radiation science practitioners to shape the future of the profession and improve patient safety through the ongoing enactment of the See Something, Say Something framework in clinical practice.

**References**

1. Medical Radiation Practice Board of Australia. Policy: Communicating safely – if urgent or unexpected findings are identified. 2019. Available at https://www.medicalradiationpracticeboard.gov.au/Registration-Standards/Professional-Capabilities.aspx.
2. State Coroner's Court. Findings of Inquest: Verna Therese Hamilton. 2013.
3. State Coroner's Court. Findings of Inquest: MAYELL Edward John. 2017.

## AngioCT – the new normal for interventional radiology

### Carmel Mcerlean,^1^ Samantha McKay^1^

#### ^1^Sunshine Coast University Hospital, Birtinya, Australia

Since 2017, with the commissioning of a new hospital, interventional radiology has become a satellite department co‐located within the theatre complex, performing approximately 150 examinations per month including acute, emergency and outpatient presentations.

On moving to the new hospital site in 2017, the interventional radiology team have been fortunate to have access to and the ability to use the innovative technology, AngioCT. The first of its type in Australia, it combines a Canon 160 slice Aquillion Prime CT and an Infinix Angio System to form the ultimate interventional radiology suite.

Having a room in a unique environment has come with challenges regarding who governs this space and staff training. Now, interventional radiology radiographers must also be adaptable CT radiographers. Having this multi‐modality room has allowed management of routine patients, such as nephrostomies and gastrointestinal bleeds, with more procedural confidence. There has also been the opportunity to treat complex pathologies such as cryo‐ablation of lung tumours and acute trauma in a unique way. The AngioCT space in a hybrid theatre allows a one‐room approach to treatment techniques and practices. This ultimately contributes to improved patient outcomes and, at times, provides the ability to perform a combination of treatment options in one space to give better time efficient patient outcomes. Cases such as acute post‐partum bleeding and 24 hours post‐trauma patients requiring interventional radiology with surgical intervention.

The future for this space is huge in Australia. This is just the beginning of how this amazing technology might be used in the future.

## Sunday 30 April, 11:00 AM–12:30 PM

## Prostate – NM

## Establishing a theranostics program: a US perspective

### Dmitry Beyder^1,2^

#### ^1^SNMMI, St. Louis, United States ^2^Barnes‐Jewish Hospital, St. Louis, United States

Launching a theranostics clinical program in the United States can have its challenges, but none are insurmountable. Modern radiopharmaceutical therapeutic medicine is bringing many benefits to health care; starting, and most importantly providing patients with opportunities for improved quality and length of life while subsequently allowing health care to take the next step to advance the delivery of therapeutic medicine and improving the role of molecular imaging in the continuum of care.

The first essential step to successfully launching a new program is to understand and follow structured process improvement by selecting an effective improvement framework and then committing to the steps necessary over the course of the change. As process improvement steps are implemented, attention to understanding and establishing the stakeholders involved, receiving input from parties who can benefit from the work, and then collaborating with stakeholders towards the finish line all lead to a successful program launch. Building a project team, establishing an executive team for project support and then developing a vision and direction for the work are integral components for sustained success. Focussing on financials, program costs, budget planning, developing professional clinical support and expertise and securing the treatment space all need to be formalised to start treating patients. Finally, moving towards becoming a distinguished therapy centre of excellence becomes an incredible distinction for the institution and the patients served, ultimately becoming a model for other facilities who aim to launch their own theranostics clinical programs worldwide.

